# Identifying the location-dependent adipose tissue bacterial DNA signatures in obese patients that predict body weight loss

**DOI:** 10.1080/19490976.2024.2439105

**Published:** 2024-12-23

**Authors:** Matthieu Minty, Alberic Germain, Jiuwen Sun, Gracia Kaglan, Florence Servant, Benjamin Lelouvier, Emiri Misselis, Radu Mircea Neagoe, Menghini Rossella, Marina Cardellini, Rémy Burcelin, Massimo Federici, José Manuel Fernandez-Real, Vincent Blasco-Baque

**Affiliations:** a Institut National de la Santé et de la Recherche Médicale (INSERM), InCOMM Intestine ClinicOralOmics Metabolism & Microbiota UMR1297 Inserm / Université Toulouse III, Toulouse, France; b Université Paul Sabatier (UPS), Unité Mixte de Recherche (UMR) 1297, Institut des Maladies Métaboliques et Cardiovasculaires (I2MC), Toulouse, Cedex, France; c VAIOMER, Microbiome, Labège, France; d Science and Technology “George Emil Palade” Tîrgu Mures, Second Department of Surgery, Emergency Mureş County Hospital, University of Medicine Pharmacy, Târgu Mureș, Romania; e Department of Systems Medicine, University of Rome “Tor Vergata”, Rome, Italy; f Department of Diabetes, Endocrinology and Nutrition, University Hospital of Girona ‘Dr Josep Trueta’; g Institut d’Investigacio Biomedica de Girona IdibGi, CIBER Fisiopatologia de la Obesidad y Nutricion, Girona, Spain

**Keywords:** Bariatric surgery, tissue microbiota, functional metagenomic, adipose fat pads

## Abstract

Recent sets of evidence have described profiles of 16S rDNA sequences in host tissues, notably in fat pads that are significantly overrepresented and can serve as signatures of metabolic disease. However, these recent and original observations need to be further detailed and functionally defined. Here, using state-of-the-art targeted DNA sequencing and discriminant predictive approaches, we describe, from the longitudinal FLORINASH cohort of patients who underwent bariatric surgery, visceral, and subcutaneous fat pad-specific bacterial 16SrRNA signatures. The corresponding *Porphyromonadaceae*, *Campylobacteraceae*, *Prevotellaceae*, *Actimomycetaceae*, *Veillonellaceae*, *Anaerivoracaceae*, *Fusobacteriaceae*, and the *Clostridium family XI* 16SrRNA DNA segment profiles are signatures of the subcutaneous adipose depot while *Pseudomonadaceae* and *Micrococcacecae*, 16SrRNA DNA sequence profiles characterize the visceral adipose depot. In addition, we have further identified that a specific pre-bariatric surgery adipose tissue bacterial DNA signature predicts the efficacy of body weight loss in obese patients 5–10 years after the surgery. 16SrRNA signatures discriminate (ROC ~ 1) the patients who did not maintain bodyweight loss and those who did. Second, from the 16SrRNA sequences we infer potential pathways suggestive of catabolic biochemical activities that could be signatures of subcutaneous adipose depots that predict body weight loss.

## Introduction

Two decades ago, the original physiological role played by gut microbiota on the control of body weight gain was discovered in mice.^1–4^ Although causality cannot be demonstrated in humans, many observations have demonstrated that obese patients are characterized by a fecal microbiota dysbiosis.^5,6^ In rodent models of obesity, some molecular mechanisms have been proposed, where an increased energy harvesting^3^ or the role of proinflammatory molecules such as the lipopolysaccharides (LPS) from gram-negative bacteria, have major impacts on body weight gain.^4^ An increased blood concentration of LPS, as characterized by metabolic endotoxemia, and its corresponding biomarker LPS Binding Protein (LBP) characterize obese patients^7^ and even children with obesity.^8^ Metabolic endotoxemia could control preadipocyte proliferation,^9^ thereby dramatically changing the gene expression pattern.^10^ More recently, we discovered that intestinal leakiness characterizing the gut from obese patients could lead to the translocation of bacteria from the intestine to tissues, notably adipose, and liver.^11,12^ In addition, another recent study has revealed that visceral adipose tissue (VAT) in obese patients, collected under sterile conditions, contains a diverse bacterial community from over 13 phyla, likely of intestinal origin.^13^ This diversity is characterized by the enrichment of certain bacterial families, such as *Streptococcaceae* and an unnamed genus in the family *Ruminococcaceae*, in obese patients, while other taxa, such as Bacilliales and the genus *Marvinobryantia*, are more prevalent in the VAT of lean patients.^13^ The authors proposed that bacteria in adipose tissue could be a novel therapeutic target to treat metabolic disorders such as obesity. Since then, we and others have described the existence of a tissue microbiota in various human tissues including blood,^14,15^ liver,^16^ and adipose depots.^17,18^ Their role is yet unknown. We here hypothesize that the adipose tissue body fat pads, i.e. visceral, a risk factor of type 2 diabetes, and subcutaneous, rather associated with the protection against metabolic diseases, could be linked to different adipose tissue bacterial ecologies and potential functions. It is now common knowledge that different adipose depots are characterized by different metabolic functions, linked to different profiles of gene expression.^19^ Similarly, we here hypothesize that the adipose tissue body fat pads, i.e. visceral (a risk factor of type 2 diabetes), and subcutaneous (associated with the protection against metabolic diseases), could be linked to different adipose tissue bacterial ecologies and potential functions. While adipose tissue molecular signatures have been described in patients with type 2 diabetes^20^ and obese patients,^17,18^ it still remains to identify whether differences exist between visceral and subcutaneous adipose depots notably in obese non-diabetic patients. Furthermore, such signatures could be associated, in obese patients who have undergone bariatric surgery, to relapse from body weight loss within years after bariatric surgery.

We decided to explore a subset of obese non-diabetic patients from the FLORINASH cohort who had undergone bariatric surgery, as described.^21^ The 16S rDNA originating from live bacteria inside tissue as described by Massier et al. ^17^ were sequenced in visceral and subcutaneous adipose depots. Previous studies have demonstrated the presence of depot-specific bacterial signatures in adipose tissue. These studies have laid the groundwork for understanding the distinct microbial communities in different adipose depots. Building upon this foundation, our study employs discriminant statistical approaches to further characterize and validate these depot-specific signatures. This validation is crucial as it supports the reliability of our subsequent functional analyses based on amplicon sequencing data genes from visceral and subcutaneous adipose depots were sequenced. Prediction metagenomics was performed to raise hypotheses regarding the potential role of specific microbial molecular metabolic pathways which characterized each adipose depot. A subset of patients was followed over 5 years for their body weight loss. Long-term body weight loss is critical in such patients since between 30% and 70% of the obese patients who underwent bariatric surgery relapsed and regained weight.^22^ In our cohort, although most patients dramatically reduced their body weight over the first-year post-surgery, a significant number of patients relapsed. We here identified specific signatures of the adipose depot 16SrRNA bacterial taxa and inferred from their corresponding genomic sequences potential metabolic pathways that could be associated with body weight loss efficacy. Combined with recent discoveries of the adipose tissue microbiota,^(17,18)^ our finding of distinct bacterial DNA signatures in specific fat pad depots could suggest functional differences between subcutaneous and visceral adipose tissues. These differences could also play a role in determining the efficacy of body weight loss following bariatric surgery.

## Material and methods

### Human cohort

The study was performed in a subset of the Florinash cohort (EU-FP7 FLORINASH; Health-F2-2009 -241,913).^21^ Florinash is a cross-sectional study on severely obese patients, who underwent bariatric surgery, where inclusion criteria were age 20–70 years, body mass index (BMI) ≥35 kg/m^2^ and absence of any systemic disease and freedom from major inflammatory status or any infection 1 month before the study. The discovery cohort was formed by 63 Spanish and Italian patients (Table 1). All patients underwent subcutaneous and omental adipose tissue and liver biopsies during bariatric surgery. Informed consent in writing was obtained from each patient and the study protocol conformed to the ethical guidelines of the 1975 Declaration of Helsinki and was approved by the Ethics Committee of the Hospital Universitari Dr Josep Trueta (Comitè d’Ètica d’Investigació Clínica, approval number 2009 046) and Policlinico Tor Vergata University of Rome (Comitato Etico Indipendente, approval number 28-05-2009). The patients provided written informed consent for the adipose tissue biopsies, as described in the FLORINASH cohort.^21^

**Table 1.** Anthropometrical and clinical characteristics of study patients (*n* = 63).

Anthropometrical and clinical features	All patients n = 63
Spanish, n (%)	28 (44)
Women, n (%)	50 (79)
Age (Years)	42.8 ± 9.8
BMI (kg/m^2)^	46.4 ± 7.1
Fat mass (%)	48.6 ± 6.3
Waist Circumference (cm)	127.4 ± 15.3
Hip Cicumference (cm)	135.4 ± 19.0
Hip/Waist Ratio	1.07 ± 0.02
HbA1c (%)	5.8 ± 0.6
Glycemia (mg/dL)	100.1 ± 22.3
AUC OGTT (mmol/L/120 min)	16649 ± 3089
HOMA IR	6.75 ± 9.46
Insulin Resistance (mg/kg.min)	3.32 ± 2.08
Triglycerides (mg/dL)	118.6 ± 45.4
Total Cholesterol (mg/dL)	194.7 ± 34.0
HDL Cholesterol (mg/dL)	47.1 ± 10.5
LDL Cholesterol (mg/dL)	126.3 ± 30.1
Ultra sensitive CRP (mg/dL)	1.30 ± 1.83

### Clinical assessments

BMI was defined as body weight (kilograms) divided by the square of body height (meters). Fat mass was measured by DXA scan (Hologic DXA system) which provides detailed measurements of the body lean mass and fat mass. An index was calculated as the total mass of fat divided by the total body mass, multiplied by 100. Waist circumference was measured in the horizontal plane midway between the lowest rib and the iliac crest to the nearest 0.1 cm at the end of a normal expiration repeatedly in men and women by 3 trained nurses on 3 consecutive days, while hip circumference was measured at the largest circumference around the buttocks. Hip/Waist ratio was calculated by dividing the hip circumference by the waist circumference for everyone.

### Biochemical and molecular analyses

Serum, plasma, urine, and stool samples from all subjects were obtained on the first study day, the week prior to elective bariatric surgery. All samples were stored at −80°C. HbA1c, glycemia, and cholesterol measurements required the collection of a blood tube after applying a tourniquet. The AUC OGTT test was realized thanks to the following method, for 8 h before the test, the patient was not able to eat or drink. Then a nurse took a sample of blood from a vein in the patient’s arm and the patient then drank about 8 ounces (237 ml) of a syrupy glucose solution containing 2.6 ounces (75 g) of sugar. Two hours later, blood glucose levels were measured again. HOMA IR was measured after an injection of insulin for a period of two to three hours and the amount of glucose to infuse to maintain a constant blood glucose level was calculated.

### Adipose tissue biopsies

Patients provided written informed consent for the adipose biopsies, as described in the FLORINASH cohort.^21^ The tissue biopsies were collected during bariatric surgery using a standardized surgical technique to ensure consistency and minimize contamination as previously described by our team (Sun J et al. in 2023^23^ and Champion C).^16^ Both visceral and subcutaneous adipose tissues and the liver were excised using sterile surgical instruments. We have addressed specific qPCR analyses to control the potential contamination. Subcutaneous and omental adipose tissues were collected during surgery in RNAlater and immediately flash frozen in liquid nitrogen before storage at − 80°C.

#### 16SrDNA metagenomic sequencing of tissue samples

To ensure the accuracy of the analysis, it is important to differentiate between bacterial DNA sequences specifically present in the adipose depots and those that may be potential contaminants. It should be noted that starting in 2015 results from independent researchers were published^24–28^ sequencing the 16S rDNA from tissues such as the adipose depots and the liver, in the field of metabolic diseases, notably in human tissues. In such reports, as in the present study, the risk of bacterial contaminants from reagents was thoroughly investigated, as shown in recent publications.^29,30^ Furthermore, negative controls were systematically run in each experiment, including extraction procedure negative controls (water at DNA extraction step) and PCR negative controls (water at first PCR step). The 16S rDNA amplified by PCR from the negative controls have been sequenced and showed a very low abundance (1000-fold lower) of the contaminant when compared to the tissue 16S rDNA concentration (to be provided upon request). The bacterial gene richness of the samples was found to be several folds higher than in the control, further supporting the conclusion that the identified bacterial DNA sequences are not contaminants. It should be noted that the presence of 16S rDNA sequences does not necessarily indicate viable bacteria. Therefore, our data cannot be used to draw conclusions about live bacteria.

To ensure a low background signal from potential exogenous bacterial contaminations from reagents, experimenters, or consumables, different negative controls are systematically performed. They consist of adding molecular grade water to an empty tube, separately at the DNA extraction and PCR steps. Then, the amplification product is sequenced and performed simultaneously to the analysis of the samples. We further run two different batches of sequencing to identify potential experimental contaminants. We therefore validate that both the beta diversity analysis and the qPCR analyses show a clear separation between negative controls and liver samples (graphs showing controls are available upon request).

Our controls suggest that any exogenous bacterial contamination is at least 10 times lower than the tissue signal, meaning that it is likely to be negligible and to have only a limited impact on the taxonomic profiles of the samples, as previously reported.^24–26^

#### DNA extraction

Adipose tissue samples were transferred on dry ice to the Vaiomer Lab in Labège, France. Genomic DNA (gDNA) was extracted using an optimized tissue-specific technique as previously described.^25^ This technique was carefully designed to maximize the recovery of bacterial DNA and to minimize any risk of contamination from the environment and the reagents. The quality and quantity of extracted gDNA were monitored by gel electrophoresis (1% w/w agarose in 0.5× TBE buffer) and NanoDrop 2000 UV spectrophotometer (ThermoFisher Scientific). All gDNA samples were stored at −20°C until further processing.

#### Mouse models and qPCR methodology for bacterial DNA detection

Twelve-week-old male C57BL/6J mice were housed in a controlled environment with an inverted 12-hour daylight cycle (lights off at 10:00 a.m.), with free access to food and water. The mice were divided into two groups: one group was fed by a normal chow diet (NCD) as a control diet (*n* = 13) (A04, Villemoisson sur Orge, France), while the other group was fed by a high-fat diet (HFD) known to induce diabetes,^1^ carbohydrate-free diet (*n* = 17) for 4 weeks. The high-fat diet used in this study contained 72% fat (corn oil and lard), 28% protein, and less than 1% carbohydrate as a percentage of energy content.^31^ This diet composition is commonly used in research to induce metabolic disturbances such as impaired glucose tolerance, and it effectively mimics the dietary conditions that can lead to metabolic syndrome in humans. We evaluated the kinetic of colonization in metabolic tissue (liver/adipose tissue) and spleen by quantifying by qPCR (V3_V4 16S rDNA primers) at regular intervals after the starter of diet. DNA was extracted from the liver, spleen, and visceral adipose tissue using a classical phenol/chloroform extraction method, followed by precipitation with ice-cold 70% alcohol, then resuspended in Tris EDTA buffer and stored at −80°C. The efficiency of bacterial DNA extraction was assessed by comparing extractions with and without microbeads; using microbeads improved extraction efficiency by a factor of 10, though relative proportions between samples remained comparable. Bacterial DNA quantification was performed by qPCR targeting the V3_V4 region of 16S rDNA. This method detects bacterial signatures but does not differentiate between live and dead bacteria. We note that 16S rRNA quantification cannot be linked to bacterial viability; additional techniques, such as PMA-qPCR or bacterial culture, are required to assess viability, which are being explored in parallel studies.

### Bacterial 16S rDNA sequencing

The V3–V4 hypervariable regions of the 16S rDNA were amplified by PCR using universal primer Vaiomer 1F and Vaiomer 1 R. The first PCR reaction was carried out on a Veriti Thermal Cycler (Life Technologies) as follows: an initial denaturation step (94°C for 10 min), 35 cycles of amplification (94°C for 1 min, 68°C for 1 min and 72°C for 1 min) and a final elongation step at 72°C for 10 min. Amplicons (467 bp on the Escherichia coli reference genome) were then purified using the magnetic beads CleanNGS for DNA clean-up (CleanNA). A second PCR reaction for sample multiplexing was performed using tailor-made 6-bp unique index sequences. This second PCR step was run as follows: an initial denaturation step (94°C for 10 min), 12 cycles of amplification (94°C for 1 min, 65°C for 1 min and 72°C for 1 min), and a final elongation step at 72°C for 10 min. Amplicons were purified as described for the first PCR round. All libraries were pooled in the same quantity to generate an equivalent number of raw reads and were sequenced on a MiSeq Illumina platform (2 × 300 bp paired-end MiSeq kit v3, Illumina).

### 16S rDNA sequence analysis

The targeted metagenomic sequences were analyzed using a bioinformatics pipeline based on ‘find, rapidly, OTUs with Galaxy solution’ (FROGS v1.4.0) guidelines.^32^ In brief, after demultiplexing of barcoded Illumina paired reads, single-read sequences were cleaned and paired into longer fragments. OTUs were produced with single-linkage clustering (swarm algorithm v2.1.6). The taxonomic assignment was performed by BLAST (Blast+ v2.2.30+) against the SILVA 138.1 Parc database to determine bacterial profiles from phylum to genus, and when reachable, to species level. The following specific filters were applied for this analysis to obtain the best results: (1) the last 30 bases of reads R1 were removed; (2) the last 40 bases of reads R2 were removed; (3) The following filters were applied: amplicons with a length of <350 nt or a length of >490 nt were removed and (4) OTUs with abundance lower than 0.005% and that appear less than twice in the entire dataset were removed. Alpha and beta diversity analyses were conducted on the OTU table (Scikit-Bio v0.4.2). An 8% cut-off for OTUs was empirically determined based on the distribution of OTU abundances in our dataset. This threshold was chosen to balance sensitivity and specificity, capturing the most relevant OTUs while minimizing noise and potential false positives. Preliminary analyses indicated that this cut-off effectively includes biologically relevant OTUs with sufficient abundance while excluding low-abundance OTUs likely representing noise. A 5% threshold increased noise by including more low-abundance OTUs, whereas a 10% threshold excluded potentially relevant OTUs with intermediate abundance. Our choice aligns with methodologies in previous studies.^33^

### In silico analytical procedures and strategy

To analyze the distribution of the patients according to their anthropomorphic and biochemical features we generated a database and imported the.csv-file into the R software.

#### Normalization procedure

The ‘missMDA’ (version 1.18) R package returns an imputed dataset for clinical data and then automatically standardizes the imputed data using z-scores during PCA with FactoMineR. Visualization is facilitated using the factoextra (version 1.0.7) R package.

Our data analyses focus on the differences between samples, such as bacterial community composition (beta-diversity), discriminant analysis (sPLS-DA), and the correlation between clinical data and OTUs (rCCA). 16S rRNA sequencing data (targeted microbiota) are very sparse with significant zero-inflation. It is now commonly accepted that observed microbiome data represent relative abundances, which sum to a constant. These are compositional data influenced by sequencing artifacts, contaminant removal, and sequencing errors. To address these issues, we employed the following strategy: (1) Taxa Filtering: We removed taxa not observed more than three times in at least 5% of the samples. This protects against OTUs with small means and trivially large coefficients of variation (C.V.). (2) Compositional Data Transformation: The ALDEx2 R package was used to generate many instances of CLR-transformed data through Monte Carlo sampling from the Dirichlet distribution. This models the compositional nature of the data, ensuring comparability across samples. The preprocessed data were then used for downstream statistical analysis.

#### Taxonomic composition

The corresponding OTUs were assigned to taxa using the Blast+ v2.2.30+ software and Silvia 138.1 Parc database. Once assigned to phylum and family taxon levels, we graphically described the bacterial ecologies associated with different tissues by performing Stacked Bar Chart representation of the corresponding tissues. This visualization was generated using the ‘ggplot2‘−3.3.4 and ‘reshape2’-1.4.4 packages. The top 10 of the main normalized taxa (relative abundances) are shown at both the phylum and family levels.

#### Alpha diversity analyses

The diversity of the bacterial 16SrRNA can be quantified at the level of the alpha and beta diversity. The alpha diversity is quantified by different algorithms based on the distribution of the different OTUs. The raw count table of OTUs is imported in the R studio environment-v1.3.959 and analyzed with the ‘vegan ‘−2.*5*.7, ‘ape ‘-*5*.5, ‘dendextend ‘−1.15.1, ‘ggstatplot ‘−0.8.0 and ‘ggplot2‘−3.3.4 packages. The richness indexes such as Observed and Chao1 and the diversity index such as Shannon have been calculated for each adipose tissue thanks to the ‘diversity’ function and represented as boxplot after removing OTU’s present in any individual of the tissue.

The statistical significance was determined for each index by Wilcoxon rank-test adapted for paired data or t-test adapted for paired data if the index followed a normal distribution and had an equal variance between the adipose tissues (as for the Shannon Index). The Shannon index, or relative entropy, considers both the number of species and the abundance of the same species among individuals. The Chao1 index gives more weight to low abundance variables. In addition, we calculated the Observed diversity index which corresponds to the number of different OTUs observed in a sample.

#### Beta diversity analyses

The second level at which the diversity is calculated is the beta diversity. It refers to the calculation of dissimilarities i.e. distances. After normalization using ALDEx2, OTU-level data or family-level data (aggregated by family) was mapped into Euclidean distance. We chose Manhattan distance, because when the number of variables is small it could increase the variance of the components. Manhattan indexes from the ‘vegan’ (version 2.5–7) package were used in our study for beta-diversity analysis. We used the Aitchison distance to analyze compositional data, mapping simplex space into Euclidean space. This method accounts for the relative proportions of species in the samples, making traditional weighted or unweighted methodologies unnecessary. The Aitchison distance provides a more accurate interpretation of compositional differences between samples without requiring a phylogenetic tree. From distance matrices, Principal Coordinate Analysis (PCoA) and hierarchical clustering (method ward.D2) were calculated and graphically represented using respectively ‘ape’-5.5 and ‘dendextend’-1.15.2 packages to estimate the distances between patients from the adipose tissues.

To test whether the groups differ in terms of centroid and dispersion, a PERMANOVA statistical test was performed using ‘adonis2’. This method is based on the distances between samples. The test compares the distances of samples within the same group to the distances of samples from different groups. If the distances between samples from different groups are much larger than those within the same group, we conclude that the groups are not identical.

Significant differences were reached between groups when p < 0.05. Analysis of similarities (ANOSIM) were carried out with ‘anosim’ functions in the Vegan package Positive numbers were allocated to similarity within groups rather than between groups and the values between groups close to zero indicated no difference. Values close to zero indicate no difference between groups (i.e., similarities are the same between groups). Significance was considered reached when both a and b were true as follows: a) p-value < 0.05 and b) *R* value > 0.3. In particular, the results shown in PCoA and Boxplot could help us to see intra- and inter-variability among the two groups.

#### Discriminant analyses

To identify discriminant bacterial signatures between subcutaneous (SAT) and visceral adipose depots (VAT), the process involved several steps: (1) We conducted Sparse Partial Least Square Discriminant Analysis (sPLS-DA) using family-level counts normalized by the ALDEx2 R package. To validate the sPLS-DA model, we employed ‘Mfold’ with five-folds and fifty-fold Cross-Validation. (2) Heat maps were generated using the ‘cim’ function in ‘mixOmics’ version 6.14.1 to visualize data clustering across different components. (3) The Network function was utilized to illustrate relationships between bacteria or bacterial metabolic functions and subgroups. (4) Variable Importance in Projection (VIP) coefficients played a critical role in our analysis. VIP coefficients help identify key predictors, reduce dimensionality, perform robust feature selection, enhance model performance, and provide valuable insights into the data. This approach allowed us to highlight the most influential bacteria and pathways contributing to differences observed between groups. The selection of the most discriminant bacteria was based on VIP coefficients, which quantify the influence of each predictor (X) on the response variables (Y) in the model. In our study, Y represented different tissue groups (SAT and VAT) or different Total Weight Loss (TWL) groups (lower and upper), while X represented Operational Taxonomic Units (OTU), OTU family levels, or pathways (PICRUST2) data.

#### Regularized canonical correlation analysis

To identify if bacterial variables were associated with clinical features specifically with each depot, we combined both databases, the normalized family level of OTU and the clinical variables, to perform a Regularised Canonical Correlation Analysis (RCCA) thanks to the ‘rcc’ function in ‘mixOmics’-6.14.1. We performed a heat map and a network analysis from the RCCA analysis in the same way as for sPLS-DA analysis.

#### Functional metagenomic prediction

To generate hypotheses regarding the potential role of bacteria on the host we used PICRUSt2 to infer from the genome, of the identified taxa, metabolic pathways that could be associated with the suggestive functions of the corresponding taxa. The outcomes from this software do not demonstrate the existence of a function but are only suggestive of a potential mechanism that should be further demonstrated through functional analyses. Therefore, we draw the attention of the reader to this important limit.

To predict the functional potential of a bacterial community we inferred, from the 16S rDNA profiles, the abundance of corresponding microbial genomes and associated metabolic pathways using the PICRUSt2 (Phylogenetic Investigation of Communities by Reconstruction of Unobserved States v2) algorithm. The prediction uses the total OTU table and FASTA file. The processes of PICRUSt followed the PICRUSt2 tutorial (https://github.com/picrust/picrust2/wiki) and generated pathway and enzyme code abundance tables which were imported into the different analyses as used for the OTUs, PCA, beta diversity, sPLS-DA, Network, Random Forest, and RCCA. In addition to these analyses, we performed a graphic representation of the discriminant inferred metabolic pathways thanks to Ipath3 software (https://pathways.embl.de). We then colored in orange the pathways discriminating visceral adipose tissue and in blue the pathways discriminating subcutaneous adipose tissue. To verify the results of the discriminant analyses VIP coefficients, statistical significance was determined for each discriminating pathways by Wilcoxon rank-test adapted for paired data or t-test adapted for paired data if the index follows a normal distribution and has an equal variance between the adipose tissues.

#### Prediction of body weight loss

Thirty-four patients from the FLORINASH cohort have been followed between 1 and 10 years following bariatric surgery. For this study, we included only those patients followed up for at least 1 year (on average 5 years) after bariatric surgery. We divided the group according to the median of total weight loss (TWL) (33.415%). The TWL index was calculated as follows: %TWL = 100%× BMI loss/(BMI).

The mean and standard deviation was calculated for each clinical variable, as well as the comparisons of the variables between the group above the median (Upper group) of the TWL and the one below (Lower group). Statistical significance was determined for each clinical variables by Wilcoxon rank-test adapted for paired data or t-test adapted for paired data if the index followed a normal distribution and had an equal variance between the adipose tissues.

#### Adipose tissue 16SrRNA abundances and bodyweight loss prediction following bariatric surgery

In order to predict weight loss 5–10 years after bariatric surgery, we performed similar analyses as for the subcutaneous and visceral 16SrRNA analyses: Taxonomic, beta diversity, sPLS-DA and network analyses between median groups in each tissue. To verify the results of the discriminant analyses, VIP coefficients and statistical significance were determined for each discriminating bacteria by the Wilcoxon rank-test adapted for paired data or the t-test adapted for paired data if the index follows a normal distribution and had an equal variance between the adipose tissues.

#### Inferred bacterial metabolic pathway abundances in adipose depots predict body weight loss following bariatric surgery

In order to identify bacterial metabolic pathways predicting bodyweight loss 5–10 years following bariatric surgery, we performed sPLS-DA, network, and Ipath3 analyses between median groups in each tissue. To verify the results of the discriminant analyses, statistical significance was determined for each discriminating pathway by the Wilcoxon rank-test adapted for paired data or the t-test adapted for paired data if the index followed a normal distribution and had an equal variance between the adipose tissues.

## Results

### Cohort clinical parameters

**Figure 1.**
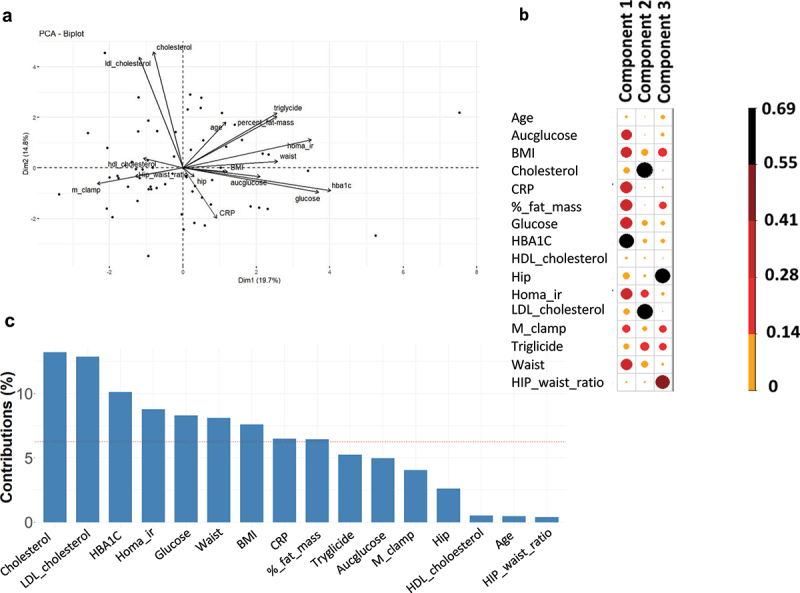
Graphic representation of the clinical and biochemical features of the patients. (a) biplot of the principal component analysis (PCA). the patients’ clinical parameters were analyzed, and the first two principal components (Dim1, Dim2) are shown. Black arrows represent the vectors of the clinical parameters. (b) importance of each clinical feature within the first four components (dim) and the corresponding scores associated with each variable. The size of the dots (as indicated by the scale) and the color intensity are proportional to the importance of the clinical variable within each component. (c) bar plot of the most important contributing variables in principal component 1. the contribution in percentage to the variance for each clinical variable within component 1 is shown.

Obese patients from the FLORINASH (FP7) cohort were recruited, as described.^21^ To analyze the distribution of the patients based upon their anthropomorphic and clinical features (Table 1) a principal component analysis was performed (Figure 1(a)). Although some outliers were observed, the first two components explained about 34.5% of the total variance. Interestingly, the main variables driving component one were related to the glycemic control (AUC glucose, HBA1C) and to insulin resistance (M_clamp; Homa_ir) (Abbreviations & Figures 1(b,c)). For the second component, variables related to cholesterol were the dominant ones (HDL_cholesterol, Cholesterol, LDL_cholesterol).

### Adipose tissue bacterial DNA analyses

To identify the 16S rRNA signatures specific or common to adipose depots, we sequenced the 16S rDNA present in the subcutaneous and the visceral fat pads from the same patients and compared them to those from liver. We first normalized the sequencing data per individual and generated instances of CLR-transformed data through Monte Carlo sampling from the Dirichlet distribution. Then, we analyzed the principal components of the normalized database and graphically represented the distribution of the individual samples (Figure 2(a)). The biplot PCA graph shows that the 16SrRNA bacterial signatures from both the visceral and the subcutaneous adipose depots, were different from that of the liver (Figure 2(a)). The *Enterobacteriaceae* and *Flavobacteriaceae* were mostly associated with the liver while the *Corynebacteriaceae* were discriminately associated with the adipose tissues. Between adipose fat pads, the bacterial signatures mostly overlapped, showing no clear discriminant signature (Figure 2(b)).

**Figure 2.**
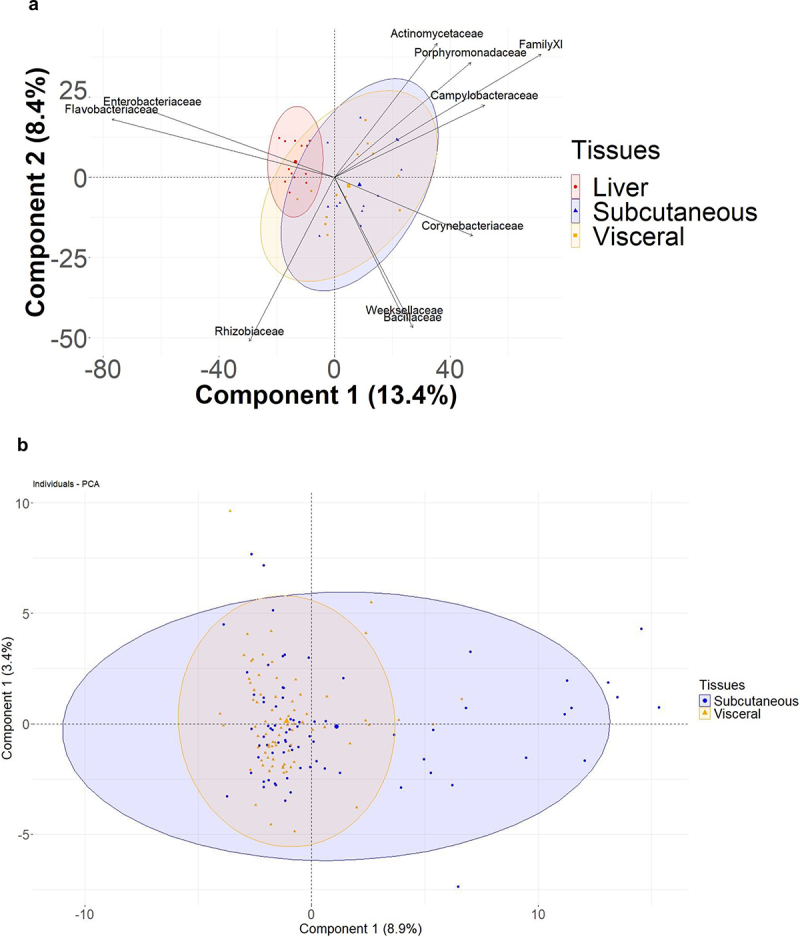
Graphic representation of the metagenomic data of the patients. (a, b) Principal component analysis of 16S rRNA sequences at the family taxonomic level showing the first and second components. (a) displays ellipses comparing the signatures of the liver, subcutaneous, and visceral fat pads; (b) shows ellipses of the adipose depots only. (c) stacked bar chart of the most contributing phyla. Their contribution in percentage is shown for subcutaneous, visceral, and pooled analysis (total). (d, f) bar plots of the relative abundance of the different taxa per individual and per tissue. The most significant phyla are highlighted. (e,g) graphic representation of the correspondence of the relative abundance of the different phyla between the subcutaneous and visceral fat pads of each individual patient.

**Figure 2.**
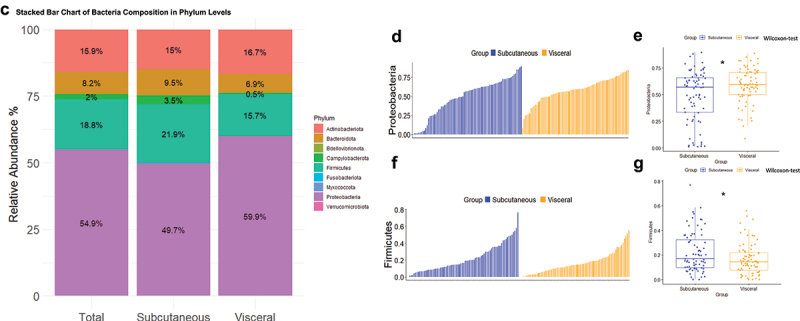
(Continued).

To further graphically describe the bacterial signatures associated with different adipose tissues, we performed a stacked bar plot analysis at the phylum and family taxonomic levels, for different individuals (Online resource Figure 2(a)). The data show that four phyla (*Proteobacteria, Firmicutes, Actinobacteria; Bacteroidetes*) mostly composed the common core bacteria DNA of the adipose tissues (Figure 2(c)). When comparing both depots we observed that the frequencies of *Firmicutes* and *Proteobacteria* were significantly different between groups (Table 2, Figures 2(d-g)) demonstrating that each tissue was characterized by its own signature. We also performed similar analyses at the family taxonomic level (Table 3, Figure 2(a)). Our results show the presence of DNA from bacteria in both adipose tissues but the major issue is to demonstrate a prior presence of live bacteria. We conducted experiments supporting our hypothesis in mice models. In reductionist models, we evaluated the kinetic of the physiological translocation of bacteria from the gut to tissues thereby establishing the “tissue microbiota.” Germ-free mice (C57bl6) from our facility were withdrawn from their germ-free environment (isolators) and cohoused with conventional mice to get naturally colonized as if they were newborns. We evaluated the kinetic of colonization in metabolic tissue (liver/adipose tissue) and spleen by quantifying by qPCR (V3_V4 16SrRNA primers) in normal mice in green or in Glycaemia disorders mice (GD mice) in red over a short period of time capturing the first instants of the colonization in Supp Online resource Figure 1(a-c). We could show that the amount of bacterial DNA was increasing over the first 2 weeks reaching a maximum after 16 weeks in liver for normal and GD mice (Online resource Figure 1(a-b)). Furthermore, it shows that the qPCR technique allows the quantification of bacterial DNA way above contaminations since no quantification could be obtained from germ-free mice before the time 0. Our data also show that the bacterial translocation process is physiologic and contributes to establishing a tissue microbiota in adipose tissue and spleen (Online resource Figure 1(b-c)). It is noteworthy that such a process is altered in Glycaemia disorders conditions providing strong pathological insights to the physiological meaning of such a process. In conclusion, we could identify the presence of bacterial DNA in tissue in metabolic disorders.

**Table 2.** Relative abundance (%) of OTUs at the phylum taxonomic level in adipose depots at inclusion of the patients. Significant differences between subcutaneous and visceral tissues are shown (data as mean ± SD. *p < 0.05, **p < 0.01, ****p < 0.0001, Wilcoxon test).

Phylum	Total (%)	Subcutaneous (%)	Visceral (%)	p value
Actinobacteriota	15.89	15.03	16.74	ns
Bacteroidota	8.22	9.52	6.93	ns
Bdellovibrionota	0.14	0.16	0.13	ns
Campylobacterota	1.97	3.45	0.50	0.0084
Firmicutes	18.77	21.85	15.73	0.042
Fusobacteriota	0.09	0.16	0.02	0.03
Myxococcota	0.02	0.04	0.008	ns
Proteobacteria	54.86	49.74	59.89	0.034
Verrucomicrobiota	0.02	0.02	0.02	ns

**Table 3.** Relative abundance (%) of OTUs at the family taxonomic level in adipose depots at inclusion of the patients. (refait 3) the top 10 most abundant families, identified with significant differences in subcutaneous and visceral tissues, are shown (data as mean ± SD, *p < 0.05, **p < 0.01, Wilcoxon test.

Family	Total (%)	Subcutaneous (%)	Visceral (%)	p value
Pseudomonadaceae	36.82	32.29	41.23	0.0028
Family XI	10.72	15.31	6.27	0.002
Moraxellaceae	8.33	7.69	8.96	ns
Micrococcaceae	8.04	7.04	9.00	0.045
Corynebacteriaceae	7.82	8.94	6.74	ns
Staphylococcaceae	7.22	8.67	5.82	ns
Sphingomonadaceae	6.50	6.61	6.39	ns
Xanthomonadaceae	5.33	5.71	4.96	ns
Comamonadaceae	4.70	4.41	4.98	ns
Rhodobacteraceae	4.48	3.29	5.63	ns

Altogether, this first set of analyses suggests that a common bacterial DNA core exists for both adipose depots. However, we also identified specific differences between depots but could not discriminate whether such changes were related to differences in the overall bacterial diversity.

### Adipose tissue bacterial 16SrRNA alpha and beta diversity indexes

**Figure 3.**
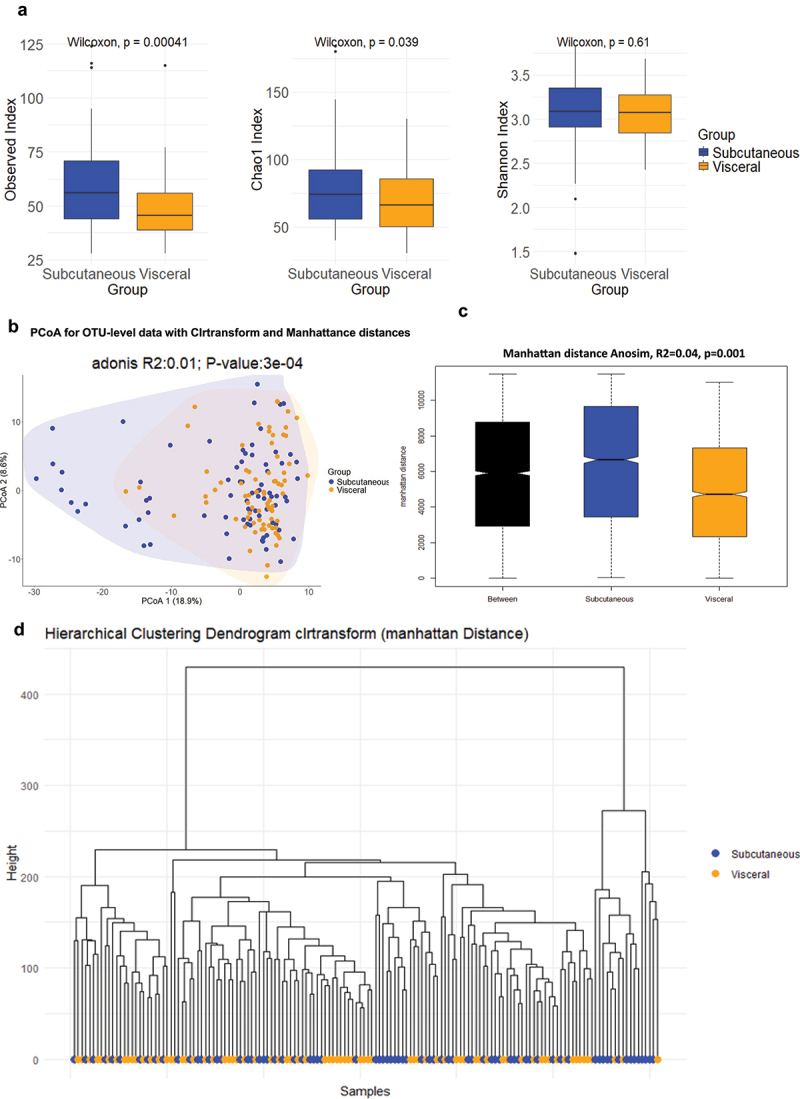
Alpha and beta diversity analyses. (a) various indices of alpha diversity. Both adipose depots were analyzed, showing correspondence between samples for each patient. Statistical significance, analyzed using the Wilcoxon test on non-parametric values of adipose tissue bacteria from different groups, is indicated by an asterisk (*). Differences between groups are shown when p-value <0.05. (b) beta diversity of the 16S rRNA sequences at the OTU level as analyzed by Manhattan principal coordinate analysis (PCoA) and PERMANOVA analysis by A?donis. (c) analysis of similarities (ANOSIM) based on Manhattan distance, showing dissimilarity between subcutaneous and visceral adipose depots. Correlation indices (r) and *p* values are shown between groups. The bold horizontal bar in each box indicates the median; the bottom bar of the box indicates the 25th percentile, and the top bar indicates the 75th percentile. The width of the bar is directly proportional to the sample size. Significance was considered reached when both conditions were met: a) p-value <0.05 and b) *R* value > 0.3. (d) hierarchical classification of patients based on their 16S rRNA sequences (at the OTU level for both tissues) using Manhattan distances and calculated with the ward.D2 method.

The number of different bacterial DNA sequences (alpha diversity) and how they are similar to each other (beta diversity) are important indexes characterizing the microbiota ecologies. To better capture the potential differences between subjects, we evaluated the diversity in bacterial richness (observed species) at large, and within high abundant (Simpson) and low abundant (Chao1) species.^34^ We therefore quantified the two corresponding indexes of diversity. We represented three currently used indexes of alpha diversity. The data show thatChao1, Observed and Shannon indexes of alpha diversity were reduced in the visceral fat pad compared to subcutaneous adipose tissue (Figures 3(a)). These indexes rather characterize low frequency taxa.

The beta diversity, where the principal coordinate analysis (PCOA) using Manhattan distances for the two principal components (1–2) is graphically represented, shows that the signature of both tissues mostly, but not fully, overlapped suggesting only subtle differences between tissues (Figure 3(b)). Therefore, the Beta diversity index was calculated using Analysis of Similarity software (ANOSIM, Figure 3(c)). Statistical significance was considered when both p-values < 0.05 and *R* values > 0.3, were observed. The corresponding hierarchical classification of the samples (Figure 3(d)) showed large heterogeneity.

Altogether this first set of analyses suggests that the two adipose depots are characterized by specific and subtle 16SrRNA signatures notably for the low frequency taxa. We therefore performed other discriminant analyses to identify more precisely the potential differences.

### Discriminant analyses of adipose tissue specific bacterial 16SrRNA signatures

To identify discriminant bacterial DNA signatures between subcutaneous and visceral adipose depots. We first identified the incidence of these discriminant bacterial DNA sequence corresponding taxa within the cohort. Principal component analysis (PCA, Figure 4(a)) and sparse partial least square (sPLS, Figure 4(b)) analyses were performed and graphically represented to identify the major bacterial families associated with the adipose depots. The biplot-PCA on the first two components of the selected Family OTUs summarized 13.6% of the variance. The data show that the *Porphyromonadaceae*, *Campylobacteraceae*, *Prevotellaceae*, *Actimomycetaceae*, *Veillonellaceae*, *Anaerivoracaceae*, *Fusobacteriaceae* and the *Clostridium, Family XI* were associated with the subcutaneous adipose depots while *Pseudomonadaceae* and *Micrococcacecae* families more specifically characterized the visceral adipose depot (Figure 4(a)). The sPLS-DA analysis similarly showed a clustering between visceral and subcutaneous tissue following tissue microbiota(sPLS, Figure 4(b,c)). The clustered image map/heat map per tissue using variables from the first component showed a specific classification of the tissues (Figure 4(d)). However, the discrimination was not absolute since some tissues were allocated to the opposite group suggesting that a signature still needs to be identified (Figure 4(d)).

**Figure 4.**
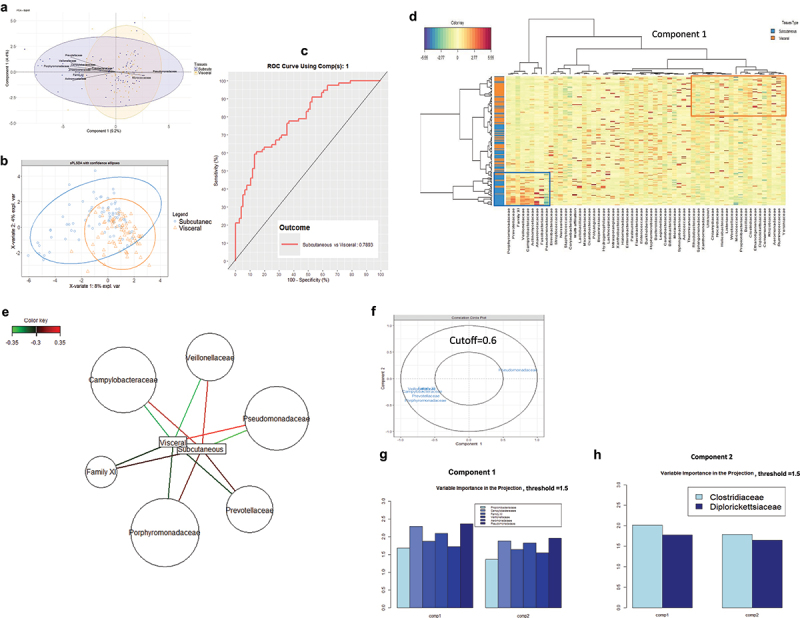
Discriminant differences of the 16S rRNA sequences for both adipose depots in each patient. (a) Graphic representation of the normalized family-level OTUs by principal component analysis (PCA). (b) sparse partial least squares discriminant analysis (sPLS-DA) of the normalized family-level OTUs. Ellipses are shown around groups of individuals and per tissue. (c) Receiver Operating Characteristic (ROC) curve analysis performed using the discriminant variables from component 1 of the sPLS-DA. Sensitivity and specificity scores are calculated. (d) clustered image maps of sPLS-DA showed classification of taxa per patient and per tissue. (e) network of sPLS-DA showing the correlation of the six most discriminant family-level bacteria and different adipose tissues. (f) correlation circle plots from the sPLS-DA applied to family-level OTUs, with a cutoff of 0.6. (g-h) variable importance in projection (VIP) coefficients for each predictor family bacteria and for each sPLS-DA component. (i, j, m, o) bar plots of the relative abundance of the different taxa per individual and per tissue. The most significant phyla are highlighted. (j, l, n, p) graphic representation of the correspondences of the relative abundance of the different phyla between the subcutaneous and visceral fat pads of each individual patient. (q, s) clustered image maps of the regularized canonical correlation analysis (RCCA) between clinical variables and family bacteria in different adipose tissues. (R) relevance network plot from the rCCA performed on the clinical variables and family bacteria. This shows the correlation structure for all bipartite relationships with a correlation above 0.2. (t) relevance network plot from the rCCA performed on the clinical variables and family-level bacteria. This shows the correlation structure for all bipartite relationships with a correlation above 0.25.

**Figure 4.**
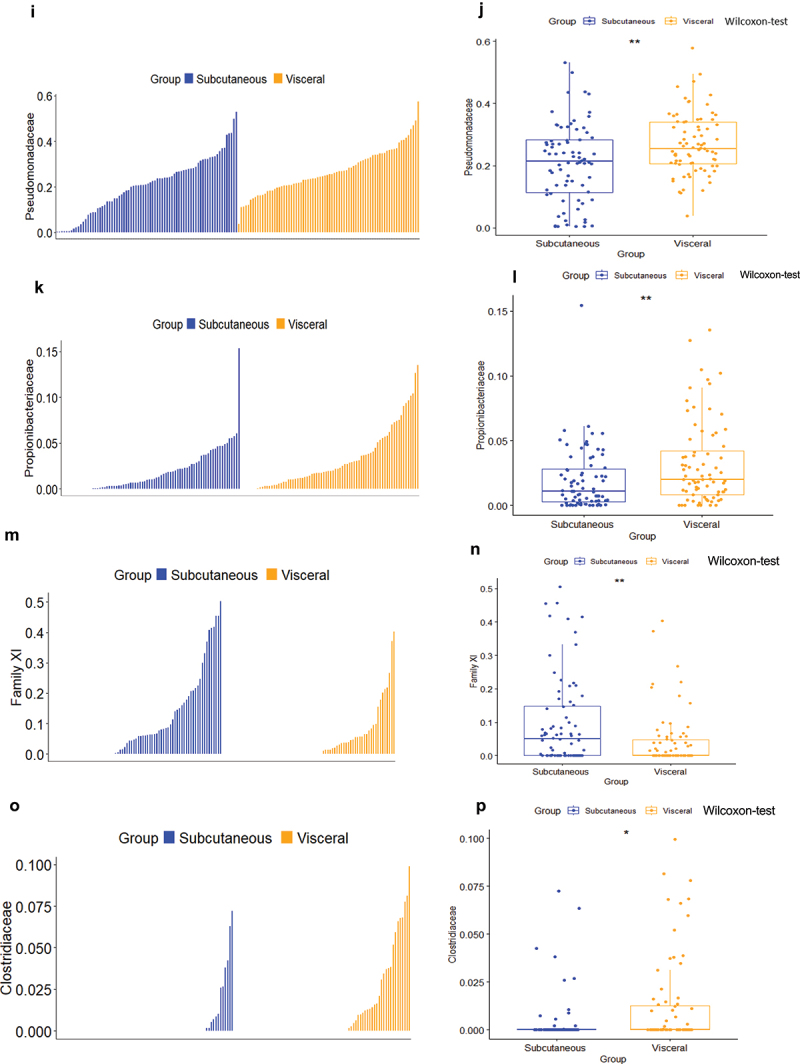
(Continued).

**Figure 4.**
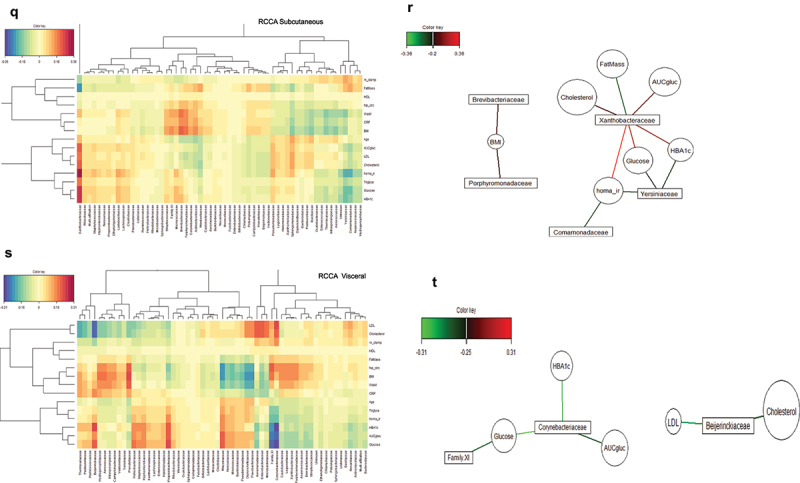
(Continued).

From the above discriminant analyses specific variables are identified and used to calculate the ROC score for sensitivity and specificity (Figure 4(c)). The ROC score was 0.79.

The network analysis from sPLS-DA reveals that *Pseudomonadaceae* is positively correlated with visceral fat, while *Campylobacteriaceae, Veillonellaceae, family XI, Prevotellaceae* and *Porphyromonadaceae* show positive correlations with subcutaneous fat, consistent with the PCA biplot (Figure 4(a) and 4e, Table 4). To further validate these findings, we employed Variable Importance in Projection (VIP), setting the threshold to 1.5 in components 1 and 2 of sPLS-DA. In component 1, *Propionibacteriaceae, Campylobacteraceae, Family XI, Veillonellaceae, Aeromonadaceae*, and *Pseudomonadaceae* were significant, while *Clostridiaceae* and *Diplorickettsiaceae* were notable in component 2 (Figure 4(f-h), Online resource Figure 3). Subsequently, we conducted Wilcoxon tests to investigate their distribution between SAT and VAT. Our analysis indicates higher abundance of *Pseudomonadaceae* and *Porphyromonadaceae* in VAT, with *Family XI* more prevalent in SAT (Figure 4 (i-p)). Due to a high prevalence of missing values across samples, other microbes were not statistically evaluated.

**Table 4.** Network correlation scores between both adipose depots from the same patient at the family taxonomic level (refait 6) *p* values are represented, and significant differences were considered when p < 0.05 using the Wilcoxon test (negative values indicate reduction).

Family	Subcutaneous	Visceral	p value
Campylobacteraceae	0.23	−0.23	ns
Family XI	0.24	−0.24	0.002
Veillonellaceae	0.25	−0.25	ns
Pseudomonadaceae	−0.28	0.28	0.0028
Clostridiaceae	−0.24	0.24	0.017

We also confirmed most of these data by running a nonlinear analysis using the supervised Random Forest analysis (Online resource Figure 4(a)).

To identify whether such bacterial DNA networks were associated with clinical features specifically for each depot we combined both databases and showed that for the subcutaneous fat depot the different glycemic indexes (homa_ir,glucose and HbA1c) were positively correlated with *Xanthobacteraceae*, and were negatively correlated with *Yersiniaceae* and *Comamonadaceae*. (Figure 4(q-r)).

Conversely, for the visceral adipose tissue, *Corynebacteriaceae* were negatively correlated with the different glycemic indexes (glucose, Glucose AUC, HbA1c), *Beijeninckiaceae* were negatively correlated with LDL (Figure 4(s-t)). These data suggest that each bacterium to adipose depot relationship could have different impacts on metabolic parameters further reinforcing the differential roles of the two fat pads in health. To refine this hypothesis, we further analyzed other indexes and from all the above analyses, we established core signatures per fat depot (Tables 5 and 6).

**Table 5.** Bacteria selected by the network and the VIP coefficient selection at the family taxonomic level specifically associated with each depot.

Methods	Subcutaneous	Visceral
Networks	Family XI	Pseudomonadaceae
	Porphyromonadaceae	Veillonellaceae
	Prevotellaceae	Campylobacteraceae
VIP	Family XI	Pseudomonadaceae
		Propionibacteriaceae
		Clostridiaceae

**Table 6.** Summary of bacteria at the family taxonomic level as signatures of each depot.

Visceral	Subcutaneous
Pseudomonadaceae	Family XI
Propionibacteriaceae	
Clostridiaceae	

### Inferred bacterial biochemical pathways in adipose depots

To infer potential bacterial biochemical pathways, thereby suggesting working hypotheses, we used the PICRUSt2 software. Due to the low bacterial biomass of our samples, the PICRUSt2 are only intended for hypothesis generation. Since this approach is based on potential genomic functions that have not been sequenced in our study, the corresponding outcomes are purely suggestive. This analysis is, however, useful to raise hypotheses thereby opening the door to discussions and further developments. The above discriminant analyses suggest that some specific bacterial DNA sequences are discriminant signatures of fat pads. However, whether the corresponding bacteria could be responsible for the functional differences observed between the two fat depots, remains to be determined since the live bacteria has not been observed in the tissue. Therefore, to generate working hypotheses regarding the potential bacterial functions interacting with the adipose depots, we inferred such functions by running metagenomics prediction algorithms i.e. PICRUSt-2 on the OTU table sets. The PICRUSt-2 analysis outcomes provide lists of enzyme codes and pathways. We focused our attention on the latter since it helps to better understand the molecular mechanisms potentially interacting with the host.

The biplot PCA on inferred pathways shows that the biochemical pathways from both groups of tissues mostly discriminate pathways in component 2 of PCA (Online resource Figure 5(a)). The PCoA of the beta diversity similarly showed overlapping signatures between groups (Online resource Figures 5(b,c)).

**Figure 5.**
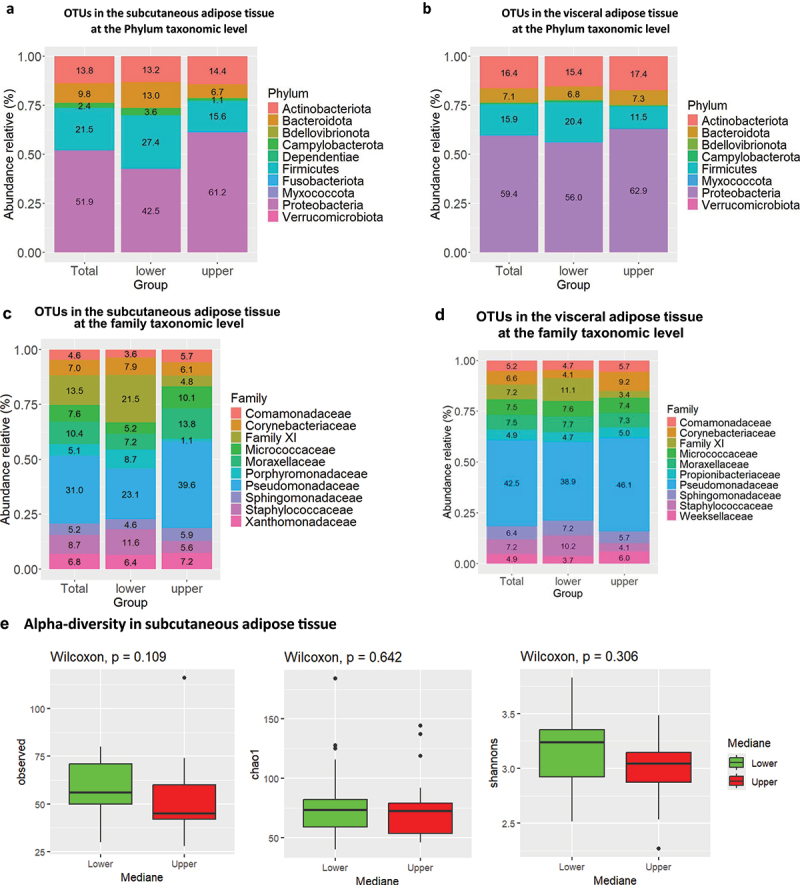
Graphic representation of the taxonomic signatures in the adipose tissues predicting body weight loss groups. (a,b) Stacked bar chart of the most contributing phyla in the subcutaneous (a) and visceral (b) adipose tissues. Their contribution in percentage is shown for in the overall cohort studied (total) and according to the groups of patients who have maintained their body weight loss (low group) or regained weight (upper group) 5–10 years after bariatric surgery. (c,d) stacked bar chart of the most contributing family in the subcutaneous (c) and visceral (d) adipose tissues. Their contribution in percentage is shown for the overall cohort studied (total) and according to the groups of patients who have maintained their body weight loss (low group) or regained weight (upper group) 5–10 years after bariatric surgery. (e,f) various indices of alpha diversity in subcutaneous (e) and visceral (f) adipose tissue. Upper and lower groups were analyzed. Statistical significance, analyzed using the Wilcoxon test on non-parametric values of adipose tissue bacteria from different groups, is indicated by the p-value (p). Differences between groups are significative when p < 0.05. (g,i) beta diversity of the 16S rRNA sequences at the OTU level as analyzed by Manhattan principal coordinate analysis (PCoA) and PERMANOVA analysis by adonis in subcutaneous (g) and visceral (i) adipose tissue. (h,j) analysis of similarities (ANOSIM) based on Manhattan distance, showing dissimilarity between lower and upper groups in subcutaneous (h) and visceral (j) adipose tissues. Correlation indices (r) and *p* values are shown between groups. The bold horizontal bar in each box indicates the median; the bottom bar of the box indicates the 25th percentile, and the top bar indicates the 75th percentile. The width of the bar is directly proportional to the sample size. Significance was considered reached when both conditions were met: a) p-value <0.05 and b) *R* value > 0.3. (k) network of sPLS-DA showing the correlation of the seven most discriminant family-level bacteria and upper and lower groups in subcutaneous tissue. (l,m) graphic representation of the correspondence of the relative abundance of different family between the TWL groups in subcutaneous tissue. (N) sample plots from sPLS-DA performed on the family according to TWL groups in subcutaneous tissue, including 95% confidence ellipses. Samples are projected into the space spanned by the first and second components. (o) correlation circle plots from the sPLS-DA applied to family data in subcutaneous tissue, with a cutoff of 0.6. (p,q) variable importance in projection (VIP) coefficients for each predictor family and for each sPLS-DA component 1 (p) and component 2 (q) in subcutaneous tissue. (r) network of sPLS-DA showing the correlation of the three most discriminant family bacteria and TWL groups in visceral tissue. (s,t,u) graphic representation of the correspondence of the relative abundance of different families between the lower and upper group in visceral tissue. (v) sample plots from sPLS-DA performed on the family-level according to TWL groups in visceral tissue, including 95% confidence ellipses. Samples are projected into the space spanned by the first and second components. (w) correlation circle plots from the sPLS-DA applied to family data in visceral tissue, with a cutoff of 0.6. (x,y) variable importance in projection (VIP) coefficients for each predictor family and for each sPLS-DA component 1 (x) and component 2 (y) in visceral tissue.

**Figure 5.**
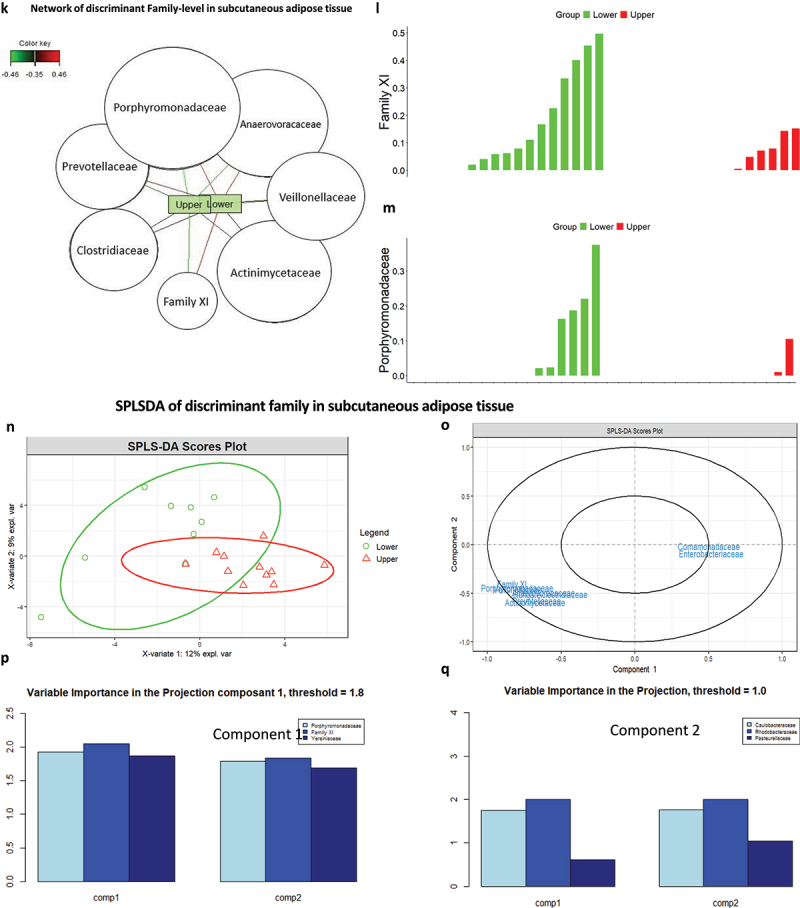
(Continued).

**Figure 5.**
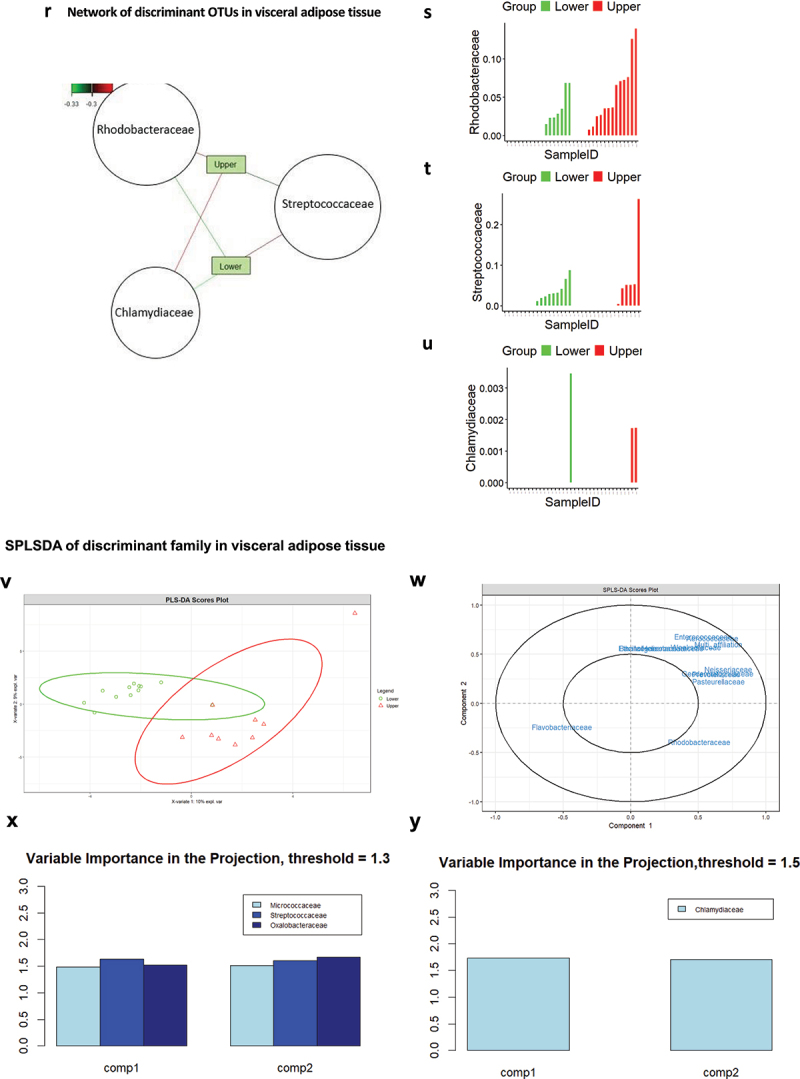
(Continued).

Since the graphical analyses of the inferred molecular pathways did not dentify major differences between groups, we then run discriminant analyses such as sPLS-DA). A heatmap was drawn, showing that the molecular pathways from both tissues could be mostly separated in component 2 (Online resource Figure 5(d)). To further validate these findings, we employed Variable Importance in Projection (VIP), setting the threshold to 1.5 in components 1 and 2 of sPLS-DA (Online resource Figure 5(e-h), Online resource Figure 7 (A-R)). On the one hand, we could identify specific pathways from microbiotal depots (superpathway of purine nucleotides de novo biosynthesis II, 5-aminoimidazole ribonucleotide biosynthesis I, S-adenosyl-L-methionine cycle I, superpathway of 5-aminoimidazole ribonucleotide biosynthesis and pyrimidine deoxyribonucleotides de novo biosynthesis II) expressed specifically in subcutaneous tissues (Online resource Figure 5(i). On the other hand, palmitoleate biosynthesis I (from (5Z)-dodec-5-enoate), cob(II)yrinate a,c-diamide biosynthesis II (late cobalt incorporation), superpathway of fatty acid biosynthesis initiation (*E. coli*) and stearate biosynthesis II (bacteria and plants) pathways from microbiota were more expressed in visceral adipose tissues than in subcutaneous tissues (Online resource Figure 5(i)).

**Figure 5.**
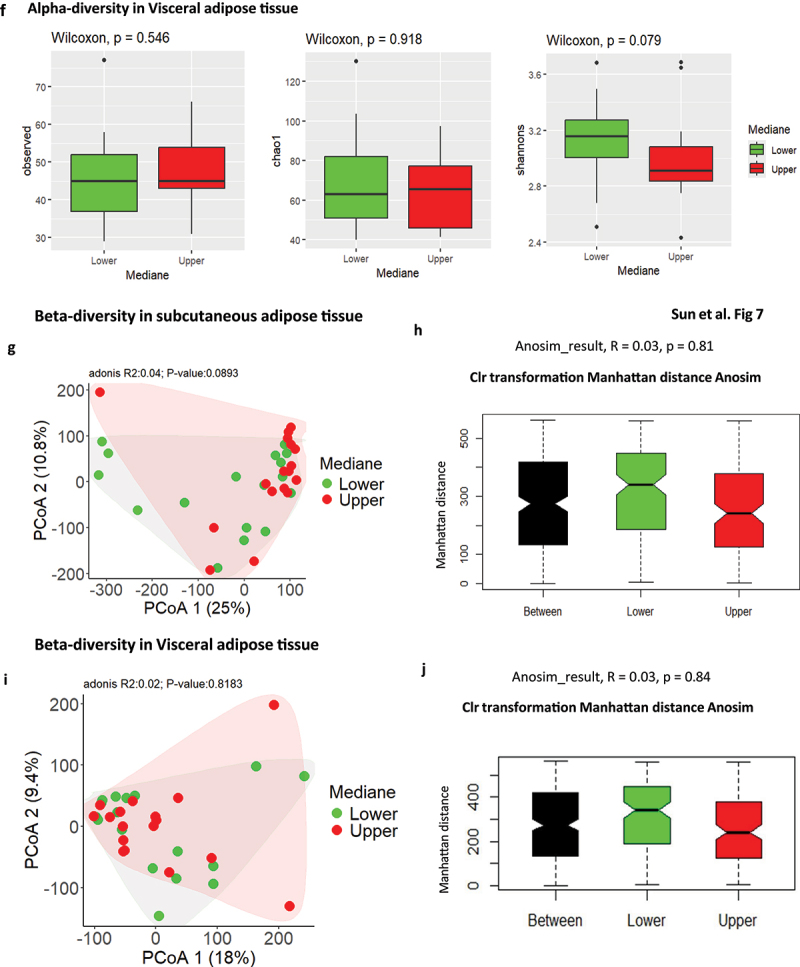
(Continued).

In addition to linear regression models, we used nonlinear regression analytical mathematical approaches to explore differences between groups and performed Random Forest analyses (Online resource Figure 7S). We observed that the vitamin pathway (PWY-5705: Allantoin degradation to glyoxylate III) was one of the most discriminant pathways to separate the different tissues.

It is notable that the aerobactin synthesis pathway was particularly and positively associated with the visceral fat pad. In addition to linear regression models, we used nonlinear regression analytical mathematical approaches to explore differences between groups and performed Random Forest analyses (Online resource Figure 7S). We observed that the vitamin pathway (pwy 6891: Thiamine metabolic pathway

PWY-5705: Allantoin degradation to glyoxylate III) was one of the most discriminant.

We identified lipid metabolism-related pathways palmitoleate biosynthesis I, (5Z)-dodecenoate biosynthesis I pathways and stearate biosynthesis II (bacteria and plants), that are significantly enriched in the visceral fat pads (metacyc: pwy: 5989, 6282), while the amino-acid related metabolic pathways were rather associated with subcutaneous adipose tissue (pwy 6891). We could also suggest that two specific pathways are potentially increased in visceral fat depots of interest: one linked to the degradation of the natural anti-oxidant allantoin (pwy-5705) and one to the biosynthesis of aerobactin, a siderophore involved in iron metabolism (aerobactinsyn-pwy) (Online resource Figure 5(i)). From this analysis, we open the door to discussions and to further demonstration of gains and losses of function that are mandatory to validate our hypothesis. Along the same line of analysis, it is important to estimate whether the potential mechanisms are correlated with clinical features to refine our hypothesis and narrow down the investigation direction.

### Correlations between inferred biochemical pathways and clinical features

To this aim, we took advantage of the richness in anthropometric and biochemical parameters from the FLORINASH cohort to perform Regularized Canonical Correlation Analyses of metabolic pathway abundance with clinical parameters. In the subcutaneous adipose depots, PWY-7003, PWY-6690, HCAMHPDEG-PWY, and PWY-6728 were positively correlated with glycemic parameters (glucose, HbA1c) (Online resource Figure 6(A,C) and Table 7, Online resource Figure 8(A)), while In visceral fat pad the network of sPLS-DA showed that PWY-3661, P381-PWY, and PWY-5347 were positively correlated with glycemic parameters (glucose, HbA1c) (Online resource Figure 6(B,D) and Table 7, Online resource Figure 8(B)).

**Table 7.** Metabolic pathways correlated with indexes of insulin resistance in both depots. Network analysis was used to select the significant and discriminant molecular pathways as signatures of both adipose depots. V: visceral, SC: subcutaneous adipose tissues.

RCCA Subcutaneous	RCCA Visceral
Glycerol degradation to butanol (PWY-7003)	Glycine betaine degradation I (PWY-3661)
Cinnamate and 3-hydroxycinnamate degradation to 2-hydroxypentadienoate (PWY-6690)	Adenosylcobalamin biosynthesis II (aerobic) (P381-PWY)
3-phenylpropanoate and 3-(3-hydroxyphenyl) propanoate degradation to 2-hydroxypentadienoate (HCAMHPDEG-PWY)	Superpathway of L-methionine biosynthesis (transsulfuration) (PWY-5347)
Methylaspartate cycle (PWY-6728)	

This important result refines our hypothesis into the concept related to the major role played by the visceral fat pad in the control of cardiometabolic diseases, while the subcutaneous fat is either protecting or neutral to these diseases. The identification of correlated bacteria mostly in the visceral fat pad reinforces their potential deleterious role in the cardiometabolic disease. Those which were still correlated also belonged to the *N*-acetylneuraminate metabolism (Online resource Figure 6(D)).

Altogether, we summarized potential metabolic pathways inferred from the 16S rDNA signatures of the adipose depots which could discriminate between depots (Online resource Figure 5(I)). Although their function could not be validated through inference analyses, we thought that they could predict a function involved in the control of the host metabolism. To reinforce this hypothesis and bring further light to the potential role of tissue bacteria we then aimed at predicting body weight loss maintenance overtime following bariatric surgery. We draw the attention that in the absence of full metagenomic sequencing, such metabolic pathways are not genomically identified and remain hypothetical. It is noticeable that the full metagenomic sequencing is not technically doable due to the huge proportion of eukaryotic DNA when compared to that of the bacteria, thereby limiting such demonstration.

### Adipose tissue 16SrRNA abundance predicts the maintenance of body weight loss following bariatric surgery

Thirty-four patients from the FLORINASH cohort have been followed 5–10 years after bariatric surgery (Table 8). We here aimed at identifying whether a 16SrRNA signature from the visceral and the subcutaneous adipose depots at inclusion could predict the body weight loss maintenance 5–10 years after bariatric surgery. It is noteworthy that patients followed for less than a year were not included since their bodyweight loss is mostly variable and intense over time course. We then identified from the median of the cohort, two groups of patients with an index of Total Weight Loss, calculated as follows. The BMI five years post-surgery minus the BMI at inclusion is divided over BMI at inclusion x100. Two groups were identified with either high (above 33.4%, higher group, those patients who did maintain high body weight loss) or others low (below 33.9%, lower, those patients who did not maintain body weight loss) (Table 8). A barplot analysis of abundances at the taxonomic phylum and family levels show differences between the two groups from the subcutaneous fat pad (Figure 5(a, c) & Table 9 and 11. A similar trend was observed for the visceral fat pad (Figure 5(b, d) & Table 10, 12). However, the quantitative difference in the abundance of certain OTUs in Chao1, Observed and Shannon of the alpha diversity indexes was not significative in TWL groups in subcutaneous tissue (Figure 5(e)) and visceral tissues (Figures 5(f)). Similarly, the beta diversity and the corresponding ANOSIM tests show no difference in TWL groups for both tissues (Figure 5(g-j)).

**Table 11.** Bacterial families specifically associated with the subcutaneous adipose depots. Network analysis and *p* values are shown when p < 0.05 and considered significant when using the Wilcoxon test.

Family	Total (%)	Lower (%)	Upper (%)	P value
Comamonadaceae	4.64	3.65	5.73	ns
Corynebacteriaceae	7.04	7.91	6.12	ns
Family XI	13.54	21.54	4.83	0.008894
Micrococcaceae	7.58	5.22	10.15	ns
Sphingomonadaceae	5.22	4.60	5.89	ns
Staphylococcaceae	8.74	11.64	5.59	ns
Moraxellaceae	10.36	7.2	13.79	ns
Porphyromonadaceae	5.07	8.71	1.12	ns
Pseudomonadaceae	30.99	23.10	39.57	0.04708
Xanthomonadaceae	6.80	6.42	7.22	ns

**Table 12.** Bacterial families specifically associated with the visceral adipose depots. Network analysis and *p* values are shown when p < 0.05 and considered significant when using the Wilcoxon test.

Family	Lower group	Upper group	p value
Acetobacteraceae	0.247	−0.247	ns
Aerococcaceae	−0.255	0.255	ns
Caulobacteraceae	0.238	−0.238	ns
Halomonadaceae	−0.310	0.310	ns
Hymenobacteraceae	−0.284	0.284	ns
Intrasporangiaceae	0.296	−0.296	0.023
Planococcaceae	−0.273	0.273	ns
Pseudonocardiaceae	−0.286	0.286	ns
Sphingomonadaceae	−0.274	0.274	ns
Unknown	0.283	−0.283	ns

**Table 8.** Clinical features of patients at inclusion, when analyzed after 5 years.

Clinical features	Mean Total ± SD	Mean Lower ± SD	Mean Upper ± SD	P value (Upper VS Lower) Wilcox.test
Age	43.2 ± 7.9	42.4 ± 7.3	44.1 ± 8.3	ns
Glucose AUC	16426.9 ± 2540.4	15995.4 ± 2118.2	16985 ± 2982	ns
Bmi	46.1 ± 4.7	47.3 ± 3.7	44.9 ± 5.8	ns
Choleste	198.7 ± 29.0	204.6 ± 31.0	192.2 ± 25.3	ns
Crp_ultr	0.864 ± 0.507	0.975 ± 0.582	0.748 ± 0.420	ns
Fat_mass	47911 ± 15875	53964 ± 15086	42570 ± 14416	0.05
Glucose	99.1 ± 13.8	95.4 ± 8.8	102.9 ± 19.2	ns
HbA1c	5.79 ± 0.39	5.7 ± 0.3	5.8 ± 0.4	ns
Hdl_chol	47.0 ± 8.1	46.4 ± 9.7	47.8 ± 6.0	ns
Hip_circ	134.1 ± 11.9	131.0 ± 13.0	137.3 ± 10.3	ns
TWL_ir	6.109 ± 4.405	4.686 ± 1.817	7.5 ± 7.1	ns
Ldl_chol	130.1 ± 24.6	135.7 ± 27.7	123.9 ± 21.0	ns
M_clamp	3.7 ± 1.7	3.5 ± 1.5	3.9 ± 1.9	ns
Triglyce	111.3 ± 31.5	115.3 ± 26.8	106.5 ± 35.8	ns
Waist_ci	126.2 ± 8.9	126.8 ± 9.1	125.6 ± 9.0	ns
Twl_5y	32.0 ± 7.7	24.5 ± 6.7	39.8 ± 2.8	4.858e-13

**Table 9.** Bacterial phyla specifically associated with the subcutaneous adipose depots. Network analysis and *p* values are shown when p < 0.05 and considered significant when using the Wilcoxon test.

Phylum	Total (%)	Lower (%)	Upper (%)	p value
Artinobacteriota	13.8	13.2	14.36	ns
Bacteriodota	9.85	13.0	6.74	0.09071
Bdellovibrionota	0.216	0.225	0.206	ns
Campylobacterota	2.37	3.61	1.13	ns
Dependentiae	0.0447	0.000827	0.0886	ns
Firmicutes	21.5	27.4	15.6	0.05086
Fusobacteriota	0.291	0	0.582	0.01922
Myxococcota	0.0166	0	0.0333	0.08033
Proteobacteria	51.9	42.5	61.2	0.03149
Verrucomicrobiota	0.0497	0.0849	0.0146	ns

**Table 10.** Bacterial phylum specifically associated with the visceral adipose depots. Network analysis and *p* values are shown when p < 0.05 and considered significant when using the Wilcoxon test.

Phylum	Total (%)	Lower (%)	Upper (%)	p value
Actinobacteriota	16.38	15.39	17.36	0.7666
Bacteroidota	7.10	6.85	7.35	0.7193
Bdellovibrionota	0.48	0.41	0.55	0.892
Campylobacterota	0.44	0.77	0.09	0.2742
Firmicutes	15.92	20.35	11.49	0.07359
Myxococcota	0.18	0.12	0.23	0.6172
Proteobacteria	59.43	55.97	62.90	0.181
Verrucomicrobiota	0.07	0.12	0.02	0.3613

Therefore, we focused our attention on abundance differences rather than on diversity and identified through discriminant analyses (sPLS-DA) that both groups could clearly be discriminated and for both tissues (Online resource Figure 9(A,B)). The corresponding ROC calculated for sensitivity and specificity showed an almost perfect predictability coefficient for both tissues (Online resource Figure 9(C,D)). In the subcutaneous fat pad, the network analysis identified families positively correlated with the groups of patients with low TWL while the same families were correlated negatively with the high TWL (Figures 5(k-m) & Table 13). To further validate these findings, we employed Variable Importance in Projection (VIP), setting the threshold to 1.8 and 1.0 in components 1 and 2 of sPLS-DA (Figure 5(n-q)), we found Family XI significantly higher in the lower groups for subcutaneous tissues (Online resource Figure 9(e-x)). We performed the same network analysis (Figure 5(r-u) & Table 14) for the visceral fat pads. To further validate these findings, we employed Variable Importance in Projection (VIP), setting the threshold to 1.3 and 1.5 in components 1 and 2 of sPLS-DA (Figure 5(v-y)). No significant differences were seen between lower and upper groups.

**Table 13.** Bacterial families specifically associated with the subcutaneous adipose depots. Network analysis and *p* values are shown when p < 0.05 and considered significant when using the Wilcoxon test.

Family	Lower_group	Upper_group	p value
Porphyromonadaceae	0.45	−0.45	0.04
Anaerovoracaceae	0.42	−0.42	ns
Veillonellaceae	0.39	−0.39	ns
Actinomycetaceae	0.35	−0.35	ns
Family XI	0.44	−0.44	ns
Clostridiaceae	0.35	−0.35	ns
Prevotellaceae	−0.39	0.39	ns

**Table 14.** Bacterial families specifically associated with the visceral adipose depots. Network analysis and *p* values are shown when p < 0.05 and considered significant when using the Wilcoxon test.

Family	Lower group	Upper group	p value
Rhodobacteraceae	−0.32	0.32	ns
Streptococcaceae	0.31	−0.31	ns
Chlamydiaceae	−0.33	0.33	ns

### Inferred bacterial metabolic pathway abundances in adipose depots predict body weight loss maintenance following bariatric surgery

Taking into account the limitation of the inferred analysis, we aimed here to identify bacterial metabolic pathways potentially predicting body weight loss. We performed a Stacked Bar Chart visualization of the dominant pathways for each group and for both tissues.

We first used Venn diagrams to select the structural zero in group TWL and both tissues (Online resource Figures 10(a,b) and Tables 15 and 16). As for the taxonomic analysis the bar charts show differences in the pathway abundance according to the group of patients (Online resource Figure 10(A-B)). To verify the distribution of the pathways in different group TWL and both tissues, we performed PCA showing no different between TWL groups in both tissues (Online resource Figure 10(C-D)). Heatmaps demonstrated separated TWL groups in two components of sPLS-DA model (Online resource Figure 10(E)). To identify pathways associated with TWL groups in both tissues, we performed Variable Importance in Projection (VIP), setting the threshold to 1.9 and 1.7 in components 1 and 2 of sPLS-DA (Online resource Figure 10(F-I)). PWY-6895, PWY-6891, PWY-7332, P42-PWY, and KDO-NAGLIPASYN pathways are significatively higher expressed in lower groups than in upper groups in subcutaneous adipose tissues (Online resource Figure 10(J), Online resource Figure 12(A-R)). For the visceral depots, the same analysis was performed to identify pathways associated with the variability of responses to bariatric surgery (Online resource Figure 11(A-F), Online resource Figure 13(B-K)). The PWY-6891, PWY-6895, and P461-PWY pathways were significatively higher in the lower group whereas the pathways were significatively higher in the upper group compared to the lower group. The corresponding ROC scores were 0.93 (SAT) and 0.93 (VAT) (Online resource Figure 12(S) and Online resource Figure 13(A)). Altogether we can here show at the taxonomic and the metabolic pathway levels that patients who succeed in maintaining body weight loss after bariatric surgery i.e. the “high” group, are characterized at inclusion by a specific adipose tissue bacterial DNA signature. A different signature was observed for patients who regained weight following bariatric surgery: the “low” group.

**Table 15.** Relative abundance (%) of pathways specifically associated with the subcutaneous adipose depots. The top 10 most abundant pathway identified with significant differences in subcutaneous tissue are shown (data as mean ± SD, *p < 0.05, **p < 0.01, Wilcoxon test).

Pathway	Total (%)	Lower (%)	Upper (%)	p value
ILEUSYN.PWY	9.51	9.55	9.45	ns
NONOXIPENT.PWY	9.15	9.34	9.00	ns
PHOSLIPSYN.PWY	9.27	9.40	9.15	ns
PWY.5101	10.24	10.21	10.22	ns
PWY.5667	10.22	10.29	10.19	ns
PWY.5973	9.85	9.77	9.94	ns
PWY.7111	12.06	11.69	12.35	ns
PWY.7663	9.96	9.90	10.05	ns
PWY0.1319	10.22	10.29	10.19	ns
VALSYN.PWY	9.51	9.56	9.46	ns

**Table 16.** Relative abundance (%) of pathways specifically associated with the visceral adipose depots. The top 10 most abundant pathways identified with significant differences in visceral tissue are shown (data as mean ± SD, *p < 0.05, **p < 0.01, Wilcoxon test).

Pathway	Total (%)	Lower (%)	Upper (%)	p value
ILEUSYN.PWY	9.68	9.65	9.72	ns
PWY.5101	10.4	10.3	10.4	ns
PWY.5667	9.83	9.88	9.79	ns
PWY.5695	8.93	8.66	9.11	0.04486
PWY.5973	9.97	9.79	10.10	ns
PWY.7094	9.30	9.44	9.22	ns
PWY.7111	12.5	12.9	12.2	ns
PWY.7663	9.90	9.77	10.00	ns
PWY0.1319	9.83	9.88	9.79	ns
VALSYN.PWY	9.68	9.65	9.72	ns

## Discussion

We here provide, usstate-of-the-art art discriminant statistical approaches, the first evidence that specific profiles of sequences of the 16SrRNA in adipose depots discriminate the visceral from the subcutaneous fat pads. Similarly, the corresponding and metabolic pathways inferred from the identification of the bacterial taxa, are suggestive of potential functional signatures. Furthermore, specific taxonomic and inferred metabolic signatures could even predict, with a very high efficacy, the maintenance or disappearance of the total weight loss in patients 5 years after bariatric surgery. Considering the limitation of the inferred conclusions we can only suggest that arginine/NAD/Allantoin/tryptophan metabolic pathways could control the maintenance of weight loss efficiency while thiamine/thiazol/methanogenesis metabolic pathways are rather associated with body weight loss regain following bariatric surgery. Such data open the door to a novel potential target specifically present in the adipose depots to predict bariatric surgery efficacy and thereby novel pharmacological intervention to improve long-term efficacy of bariatric surgery.

The existence of specific signatures of bacterial DNA within tissues has recently been confirmed by many research groups. In a study by Anhê et al.^20^ which compared microbial profiles in the plasma, liver, and adipose tissues of individuals with morbid obesity with and without type 2 diabetes (T2D), specific tissue microbial signatures were found. The study showed a higher bacterial load in the liver and omental adipose tissue, and a reduced bacterial diversity in the mesenteric adipose tissue of patients with diabetes. While our study focused on the omental depot as a representative of visceral adipose tissue, we recognize that other visceral depots, such as mesenteric, perirenal, and retroperitoneal fat may exhibit unique bacterial signatures.^17,18^ Our current findings further support the notion that each visceral depot has distinct microbiome characteristics. Several other reports have sequenced the 16S rDNA demonstrating the existence of adipose tissue bacterial DNA in humans^35–37^ and rodents^25^ at levels significantly higher than what is observed for contaminants.^11,17,25,37^ Our results are consistent with those of Massier et al.^17^ which show that bacterial DNA are present in human visceral and subcutaneous adipose tissue. The signature of tissue microbiota in adipose tissue is associated with inflammatory parameters. Massier et al. proposed the visceral tissue is the principal site of bacterial translocation from gut microbiota in metabolic disorders. The profiles of 16S rDNA sequences in host tissues, notably in fat pads, are signatures of metabolic disease. However, to generate hypotheses regarding potential roles of 16SrRNA, we needed to further define these signatures. In addition, Shantaram et al. show that the obese patients are characterized by a distinct bacterial community compared to lean patient. This bacterial infiltration is associated with the increase of neutrophils infiltrations in adipose tissue, which could contribute to inflammation of adipose tissue.^13^ They proposed to target the bacteria inside visceral adipose tissue to reduce the development of obesity.

First, using state of the art targeted 16S rDNA sequencing and discriminant predictive approaches, we here describe, from the longitudinal FLORINASH cohort of patients who underwent bariatric surgery, visceral, and subcutaneous fat pad-specific profiles of bacterial 16S rDNA as signatures. In numerous instances, the bacterial DNA sequences have been quantified and associated with some physiological features of the host. Hence, bacterial DNA sequences within tissues are signatures of clinical characteristics. We and others studied cardiometabolic disease and identified that blood bacterial DNA signatures allowed the prediction of diabetes occurrence 3–6 years ahead of time, as well as cardiovascular events^14,35^ and obesity.^18,20^ In other instances, tissue microbiota have been described as a common trait in other inflammatory diseases such as cancer.^38,39^The authors have identified live bacteria, notably from breast tumors, which were isolated and showed their causal role in the control of the chemotherapy efficacy. They used non-cancerous tissues as controls and identified live bacteria different from those found in tumors. Hence, different gut bacteria translocate to tissues according to the inflammatory disease considered. Previous reports showed correlations between adipose tissue microbiota and the host metabolic phenotypes but less is known about the tissue specificity of adipose depot microbiota signatures. This question is of importance since both subcutaneous and visceral fat pads are considered to have opposite impacts on cardiovascular and metabolic diseases. We here addressed this question and identified, using state of the art standard 16S rRNA sequencing with V3 and V4 primers and discriminant multivariate analyses, sets of bacterial DNA signatures, at the phylum and the family taxonomic levels, which discriminate between visceral and subcutaneous fat depots. We notably identified that the frequency of *Proteobacteria* was increased in visceral when compared with subcutaneous fat pads. An augmentation of this LPS gram negative group could be associated with the proinflammatory profile of visceral fat pads, as observed during obesity-associated T2D. We initially showed that the LPS produced by gram-negative *Proteobacteria* triggers adipose precursor cell and macrophage proliferation thereby providing a proinflammatory profile to the tissue^9^ as defined by the low-grade inflammation observed during metabolic diseases. Conversely, the frequency of the gram positive (without LPS) *Firmicutes* bacteria is increased in subcutaneous fat pads. The latter adipose depot is associated with the protection against the development of cardiometabolic diseases in type 2 diabetic patients. It is noteworthy that *Firmicutes* were initially identified as bacteria producing high amounts of short chain fatty acids, notably butyrate, involved in the downregulation of inflammation by favoring T regulatory lymphocyte maturation.^40^ Therefore, the reduced ratio of Firmicutes/Proteobacteria associated with visceral fat pads would be rather proinflammatory, hence fueling the low-grade inflammation associated with metabolic diseases.

The specificity of the gut bacteria which translocate to different tissues within the same individual rules out the contamination risk linked to the experimental processing of the samples. Furthermore, we show here that the adipose tissue bacterial DNA signatures are associated with different clinical features. As an example, the frequency of the *Family XI* taxon, that is increased in visceral fat pad, is tightly correlated with all diabetic features such as HbA1c, fasted glycemia and the area undercurve of the glucose tolerance test. This suggests a potential causal role in hyperglycemia. However, we cannot rule out that the presence of this taxon could be the consequence of diabetes rather than a potential cause. Regarding the biological role of the identified bacterial taxa, it remains unclear whether they confer protective effects or contribute to metabolic dysfunction. Some bacteria, such as those from the *Porphyromonadaceae* and *Pseudomonadaceae* families, have been implicated in metabolic processes, including lipid metabolism and inflammation.^41,42^ It is likely that these bacteria, whether alive or dead, influence adipose tissue function through modulating local immune responses or metabolic pathways. However, without direct mechanistic studies, it is premature to assign a definitive protective or detrimental role to these bacteria.

To further evaluate potential mechanisms responsible for or associated with the disease, there is a need to sequence the full tissue metagenome using shot gun sequencing. However, a drawback is that this technique cannot be performed on tissue DNA using current strategies since the prokaryotic to eukaryotic DNA ratio is too low and the vast majority of the sequences would originate from the host DNA rather than the bacteria. This technical limitation prevents the analysis of the potential role played by the tissue microbiota at a functional level. However, we could here still infer some potential metagenomic functions from the taxonomic identification using the latest reference databases and the PICRUSt2 functional metagenomic prediction script. Biosynthetic pathways such as numerous lipid synthesis pathways were related to the visceral fat pads. In line with these findings, it is common knowledge that the fat content from the visceral adipose depot can be rapidly mobilized by the host to release lipids to the body. Altogether, the inferred metabolic pathway signatures were fully consistent with metabolic features of the fat pads, suggesting that novel working hypotheses could be inferred from such analyses. The analysis of metabolic pathways (PWY-6895, PWY-6891, PWY-7332, P42-PWY, and KDO-NAGLIPASYN) associated with total weight loss groups in subcutaneous and visceral adipose tissues revealed significant differences between lower and upper groups. In Subcutaneous Adipose Tissues, the pathways PWY-6895, PWY-6891, PWY-7332, P42-PWY, and KDO-NAGLIPASYN are significantly more expressed in the lower groups compared to the upper groups. This suggests that these metabolic pathways may play a crucial role in the metabolism of subcutaneous adipose tissues, particularly in individuals with lower weight loss. The higher levels of these pathways might indicate a specific metabolic response or adaptation to variations in body composition within these groups. In Visceral Depots, similarly, the pathways PWY-6891, PWY-6895, and P461-PWY are significantly more expressed in the lower group, while other pathways are higher in the upper group. This observation highlights specific metabolic differences in visceral adipose depots, which are often associated with higher metabolic risks. Variations in the expression of these pathways may reflect distinct metabolic mechanisms influencing the response to bariatric surgery.

The identification of these potential metabolic pathways could provide valuable insights into the underlying mechanisms driving variability in weight loss responses following bariatric surgery, if it can be validated in further experiments. In conclusion, considering the limitation of the inferred results, we can only suggest that studying metabolic pathways in subcutaneous and visceral adipose tissues may reveal differences that could influence individual responses to weight loss after bariatric surgery. These results, if confirmed by further functional analyses offer promising perspectives for developing targeted therapeutic strategies and gaining a better understanding of the involved metabolic mechanisms.

We opted to investigate the hypothesis that adipose tissue signatures could predict the outcome of bariatric surgery, since the bodyweight loss over the years diminishes over time. Furthermore, in numerous instances, the patients entirely regain bodyweight.^22^ We therefore inquired whether a specific adipose tissue 16SrRNA signature could predict the efficacy of bariatric surgery over time. We divided the longitudinal cohort in two and generated groups of patients who fully regained body weight (lower group) or who did not regain much weight (upper group). The data show that a very discriminant signature of bacterial DNA was identified in both the subcutaneous and the visceral fat pads of patients who regain a lot of the weight versus those who did not, 5 years post-bariatric surgery. The signature is so clear that, for example, in the subcutaneous adipose tissue, the ROC index is extremely close to 1 showing how efficient the predicting score is. In both fat pads, *Porphyromonadaceae* and *Pseudomonadaceae* were major components of the ROC score. Their molecular role in tissues is unknown. However, we could infer from their genome, potential molecular pathways that could be the bases for original mechanistic hypotheses. These microbial metabolic pathways are mostly involved in aging and radical scavenging,^43,44^ which is a process that occurs during body weight gain or loss in obesity. As a potential hypothesis, we suggest that the quality of the gut microbes present within the tissues and notably the subcutaneous adipose fat pads could not only predict but be associated with some mechanisms controlling a steady fat loss over time. The long-term efficacy of bariatric surgery could therefore depend, at least in part, on the adipose tissue microbiota ecology. This idea will require further demonstration. We provide here some evidence regarding working hypotheses to understand the kinetics of bodyweight loss and recovery following bariatric surgery.

Questions remain regarding the mechanisms through which translocation establishes tissue-specific microbiota DNA signatures. The most likely mechanism is related to the leaky gut hypothesis, notably driven by the intestinal immune system. In cancer, an impaired intestinal immune system could be responsible for the large bacterial loads observed in tumors.^45^ The specific addressing of the bacteria in cancer tissues could be linked to the inflammatory status provoked by the tumoral development within the tissue. In such biological conditions, chemokines released by the activated immune cells specifically attract monocyte-derived macrophages in tissues. Similarly, white polynuclear cells, mostly present in the intestinal lamina propria are attracted to the tumor. These intestinal cells phagocytose bacteria translocated from the gut lumen and the mucosal layer, within the lamina propria of the intestine. These bacteria-loaded phagocytes, attracted by the chemokines, migrate to the inflamed tissues and further add to the inflammatory process.^46^ In addition to cancer, intestinal permeability has been characterized in animal models of metabolic disease. An increased mucosal load of bacteria has been observed in mice with high-fat-diet-induced metabolic diseases and is associated with a specific-tissue microbiota.^11^ Microbial recognition pattern receptors such as NOD1 have been shown to control bacterial translocation and hence tissue microbiota.^12^

In summary, we propose here that adipose tissue-specific bacterial DNA signatures are first associated with fat pad specificity and are secondly strong predictors of bariatric surgery efficacy over time.

## Abbreviations

Abbreviations: **Meaning**
AUC: Area Under Curve
HbA1c: Glycated Hemoglobin A1c
M clamp: Insulin resistance quantified by the hyperinsulinemic clamp
HOMA IR: Index of insulin resistance
HDL: High Density Lipoprotein
LDL: Low Density Lipoprotein
LPS: Lipopolysaccharides
LPB: LPS Binding Protein
OGTT: Oral Glucose Tolerance Test
OTU: Operating Taxonomic Unit
PCA: Principal Component Analyses
sPLS-DA: Sparse Partial Least Square Discriminant Analyses
PICRUSt2: Phylogenetic Investigation of Communities by Reconstruction of Unobserved States v2
VIP coef: Variable important projection coefficient
SAT: Subcutaneous Adipose Tissue
VAT: Visceral Adipose Tissue

## Supplementary Material

Supplemental Material

## Data Availability

The manuscript follows the CONSORT guidelines. MiSeq 16S rDNA sequences were deposited under the primary accession number PRJEB53362 and secondary number ERP138154 in June 30^th^ 2022.
