# Supplementary material for: Identifying the location-dependent adipose tissue bacterial DNA signatures in obese patients that predict body weight loss
Source: Gut Microbes. 2024 Dec 23;17(1):2439105. doi: 10.1080/19490976.2024.2439105 (PMC12931691; doi:10.1080/19490976.2024.2439105)
Supplement: Supplemental Material [file KGMI_A_2439105_SM4671.docx]

**Supplementary material**


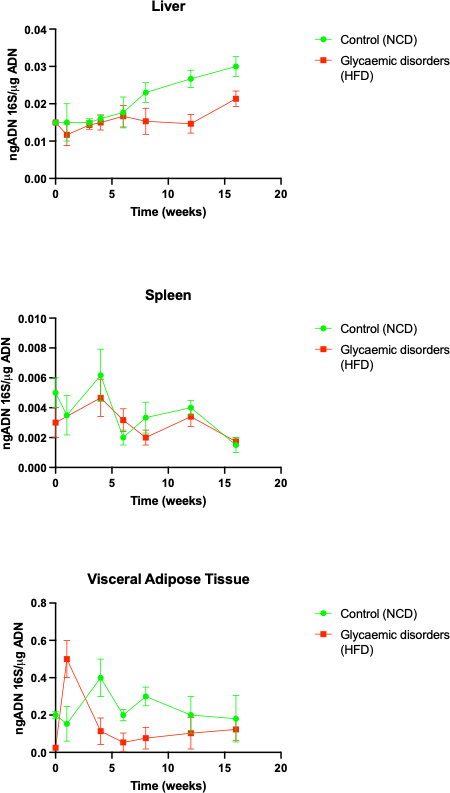


**
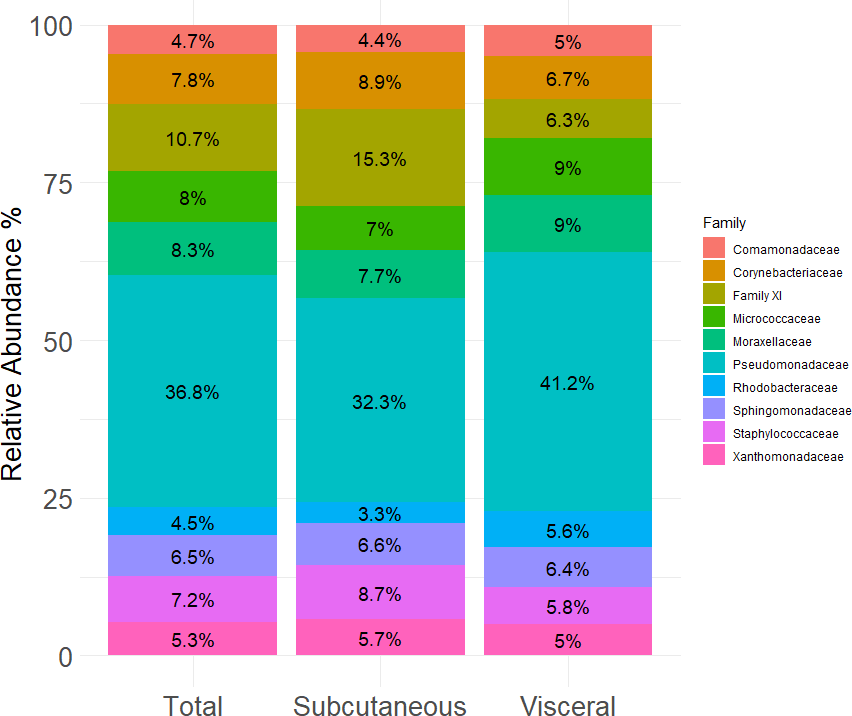
****A**

**
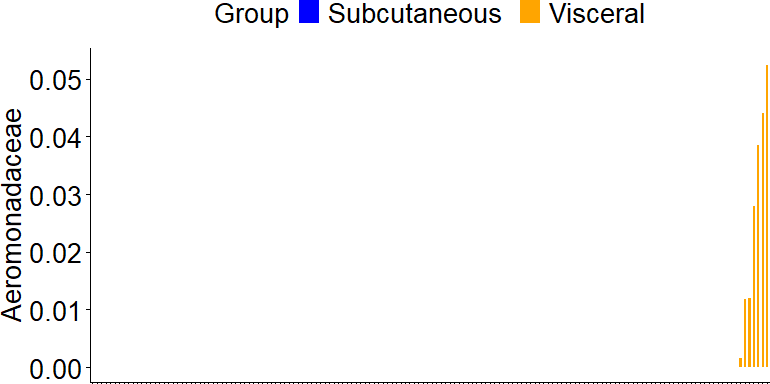
****A B**


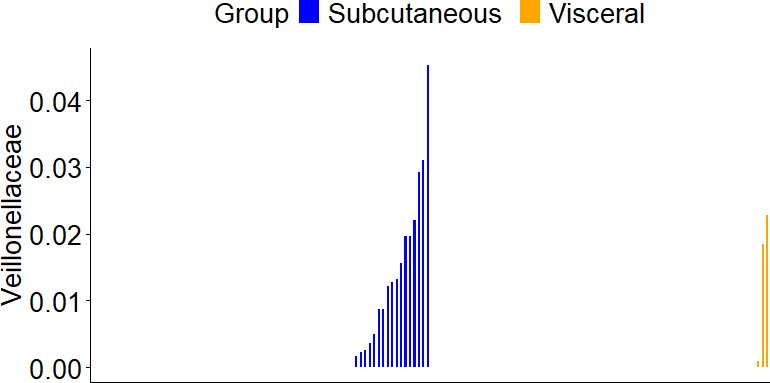

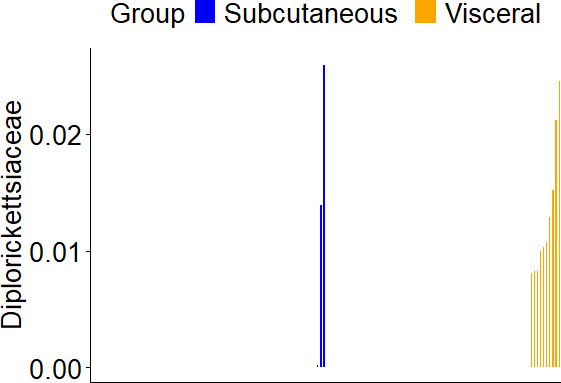


**C D**


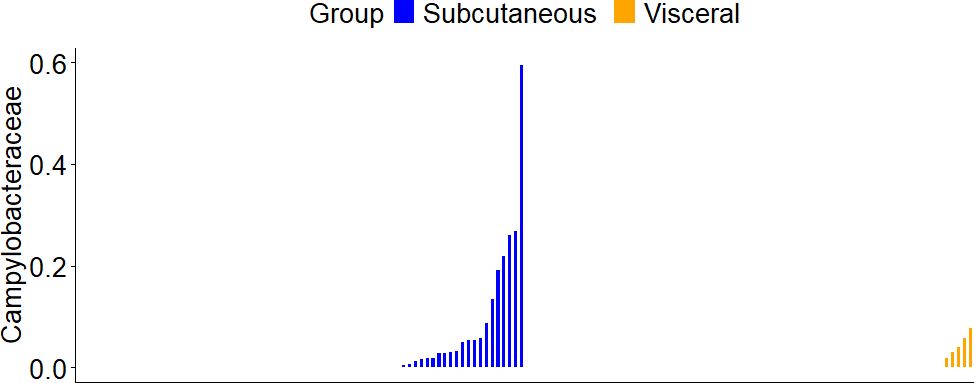


**A**


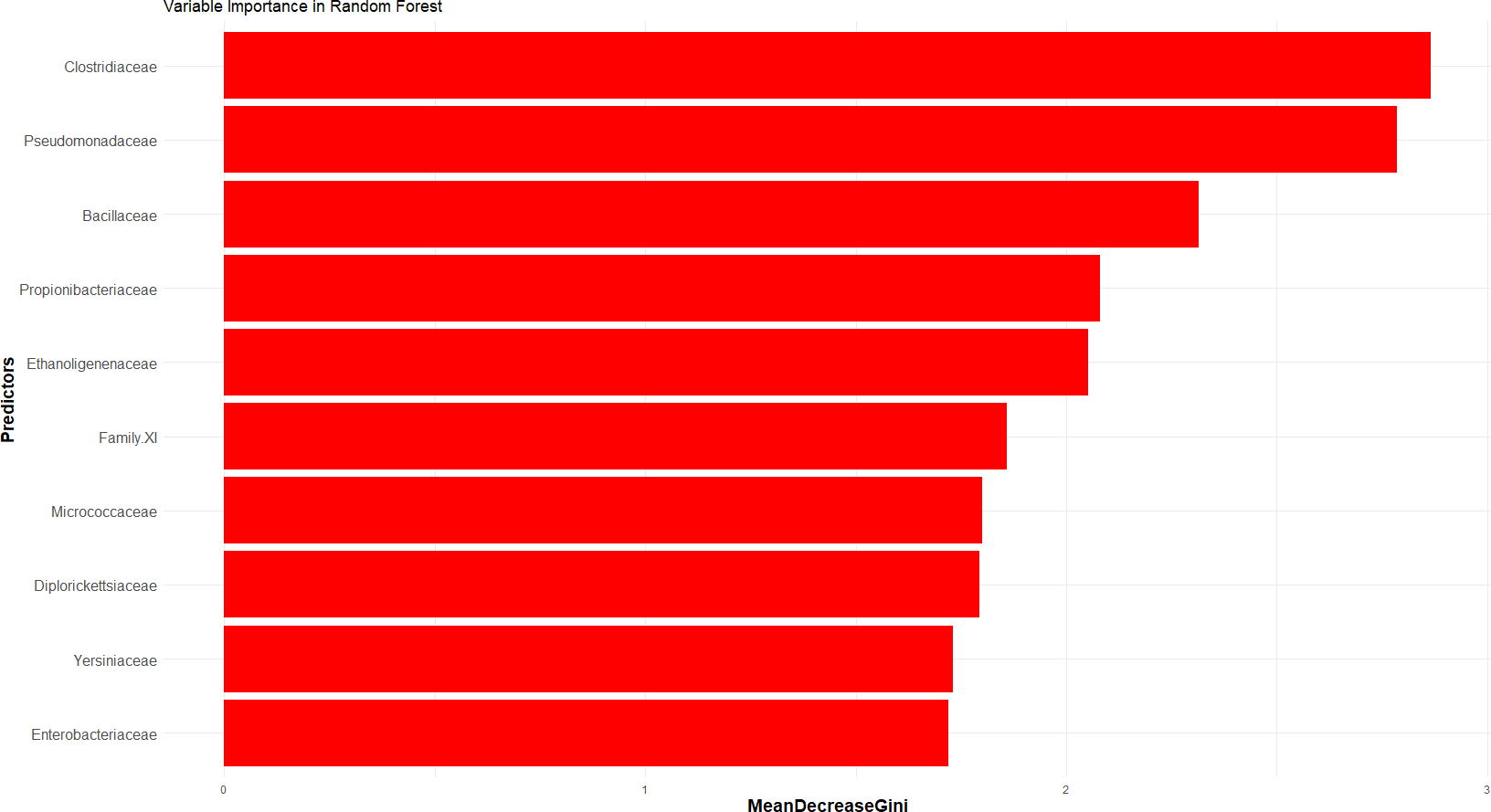


**A**


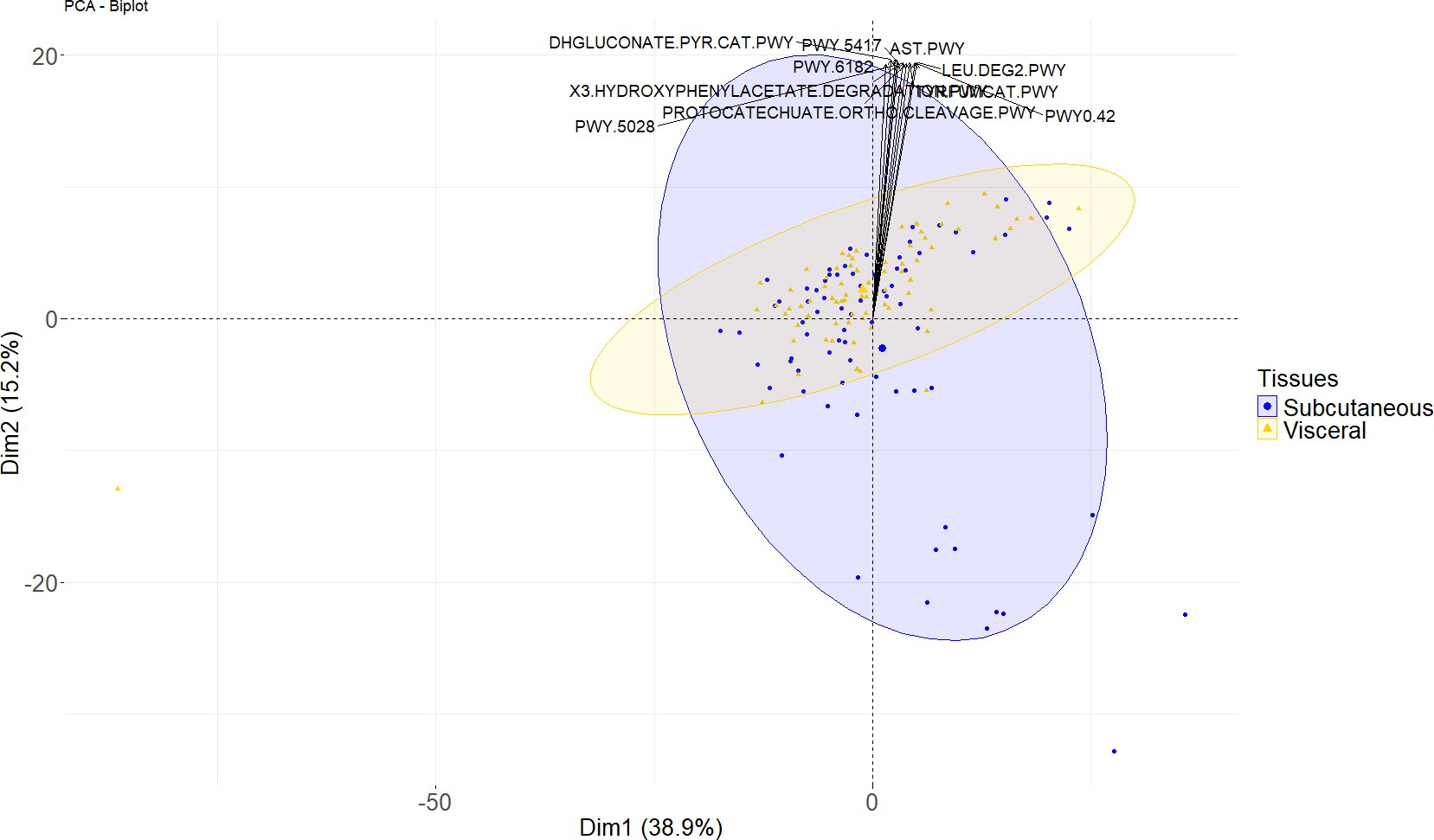


**B PCoA for Pathways-level data with Clrtransform and C**

**Manhattance distances**

**
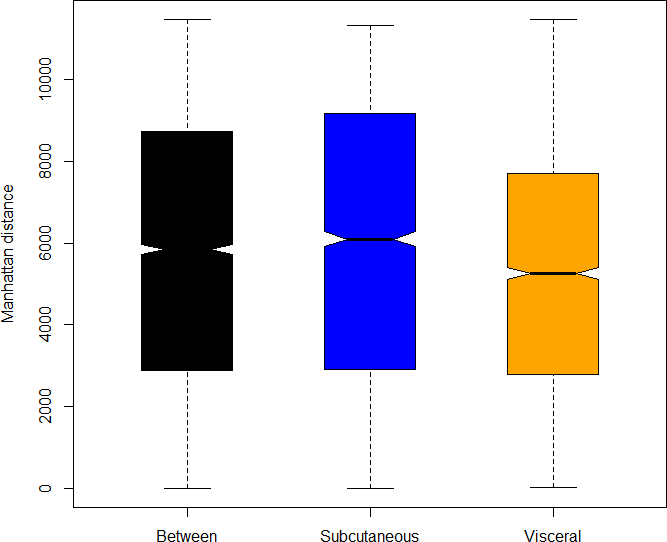
Manhattan distance Anosim, R2 = 0.03, p=0.005**


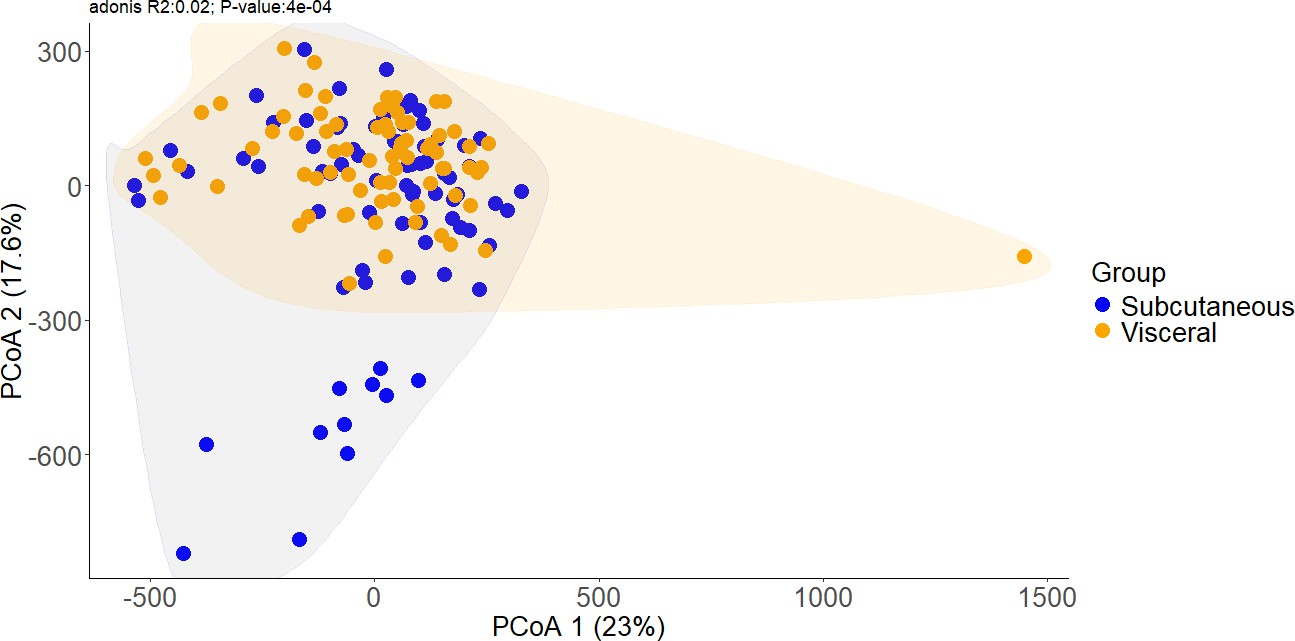

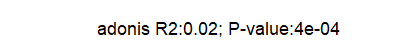


**D**


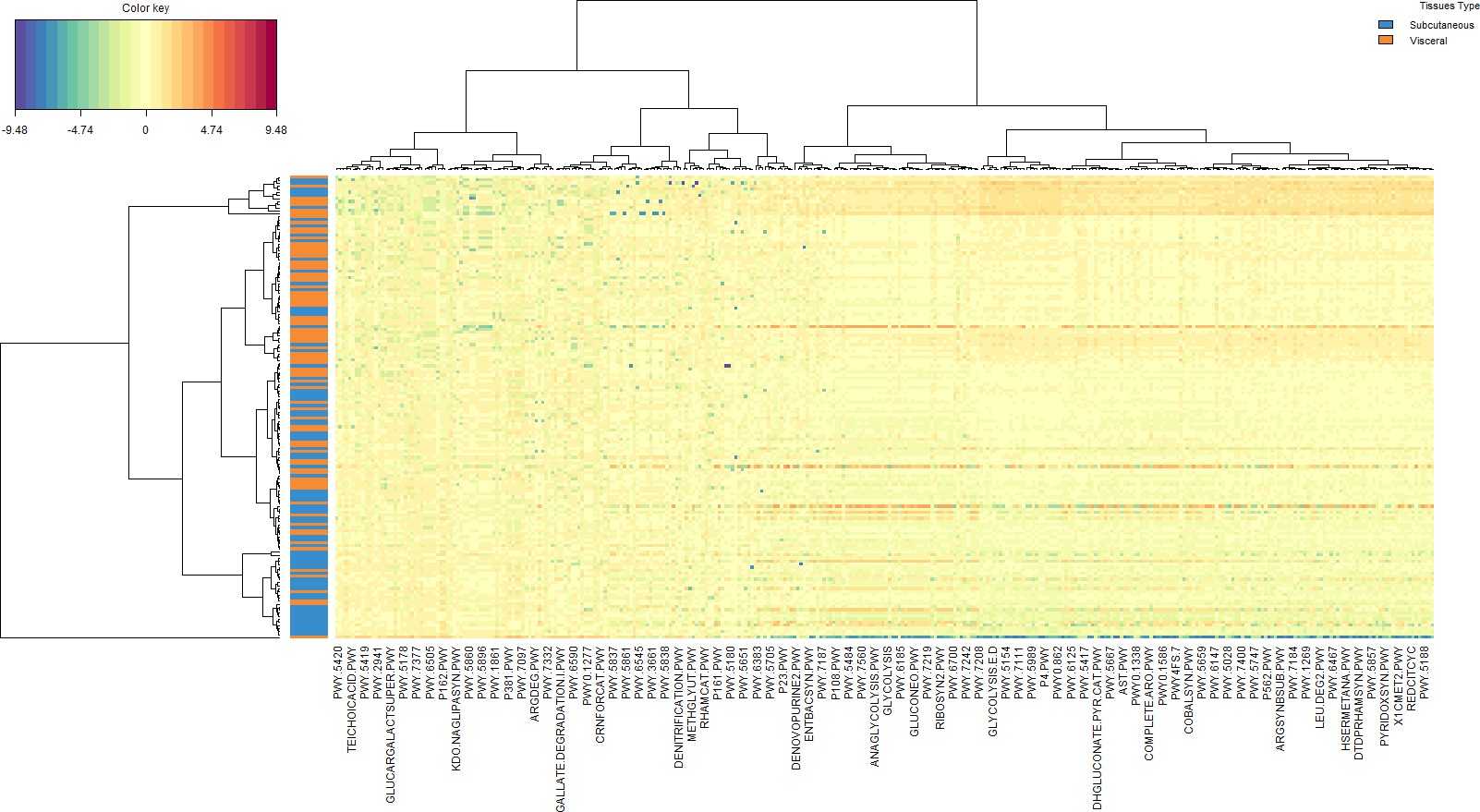

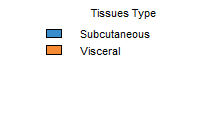


## Component 2

**
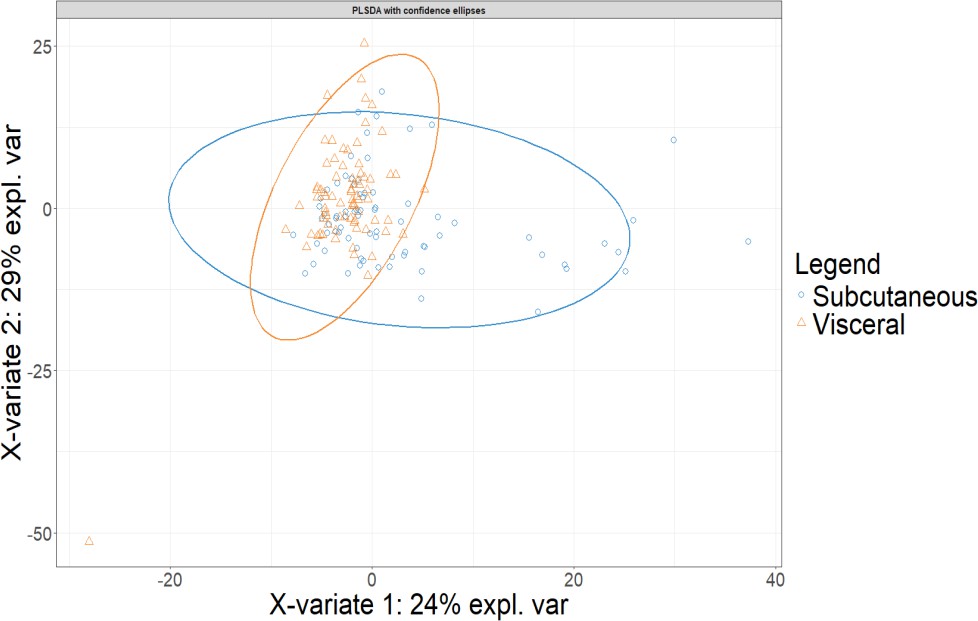

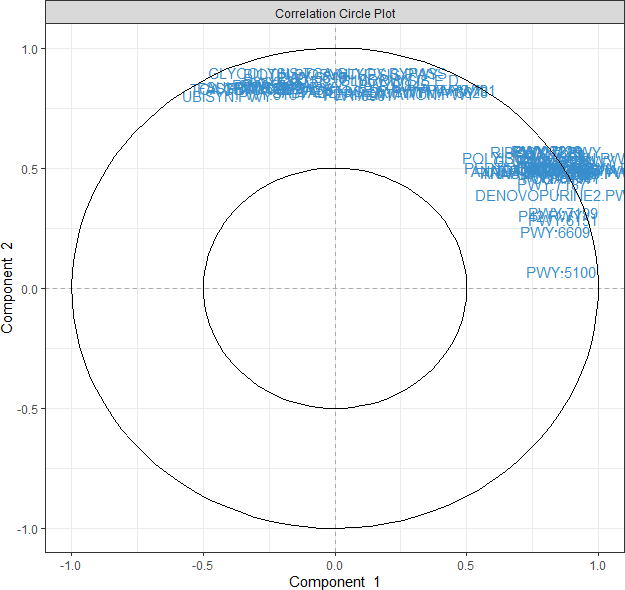
****E F**

**G**

Component 1

**H**

Component 2


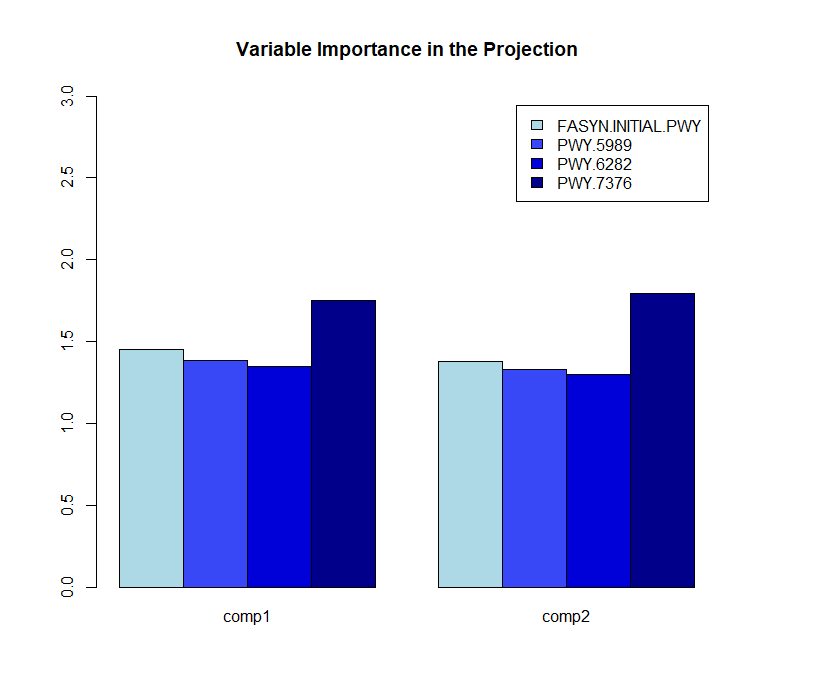


**, threshold =1.5**

**, threshold =1.5**


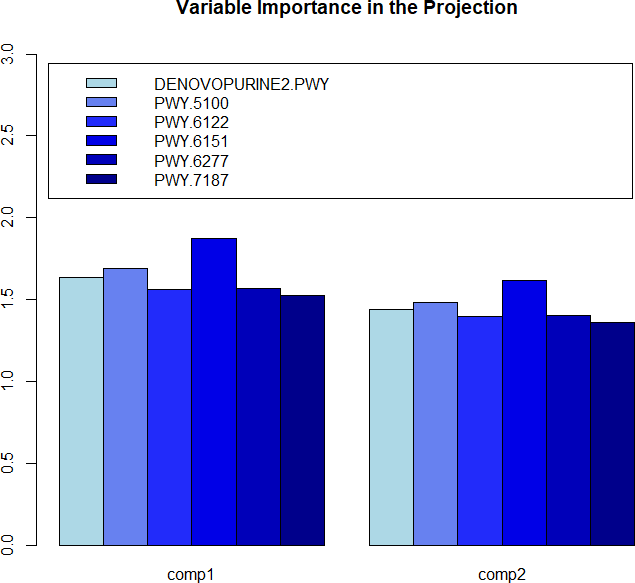


**, threshold =1.5**

#
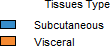
I

| **Pathway** | **Function** | **Subcutaneous**  **(%)** | **Visceral**  **(%)** | **P-value (S vs V)**  **(Wilcoxon test)** |
| --- | --- | --- | --- | --- |
| DENOVOPURINE2.PWY | superpathway of purine nucleotides de novo biosynthesis II | 0.50±0.12 | 0.44±0.09 | 0.0034 |
| PWY.6122 | 5-aminoimidazole ribonucleotide biosynthesis I | 0.67±0.15 | 0.58±0.06 | 0.00033 |
| PWY.6151 | S-adenosyl-L-methionine cycle I | 0.37±0.12 | 0.31±0.07 | 0.0010 |
| PWY.6277 | superpathway of 5-aminoimidazole ribonucleotide biosynthesis | 0.67±0.15 | 0.59±0.06 | 0.00033 |
| PWY.7187 | pyrimidine deoxyribonucleotides de novo biosynthesis II | 0.39±0.08 | 0.36±0.07 | 0.006 |
| PWY.6282 | palmitoleate biosynthesis I (from (5Z)-dodec-5- enoate) | 0.51±0.20 | 0.60±0.09 | 0.0035 |
| PWY.7376 | cob(II)yrinate a,c-diamide biosynthesis II (late  cobalt incorporation) | 0.17±0.06 | 0.21±0.04 | 2.1 e-05 |
| FASYN.INITIAL.PWY | superpathway of fatty acid biosynthesis initiation  (E. coli) | 0.46±0.18 | 0.55±0.09 | 0.0039 |
| PWY.5989 | stearate biosynthesis II (bacteria and plants) | 0.52±0.20 | 0.62±0.09 | 0.0030 |

**A**

Subcutaneous component 1


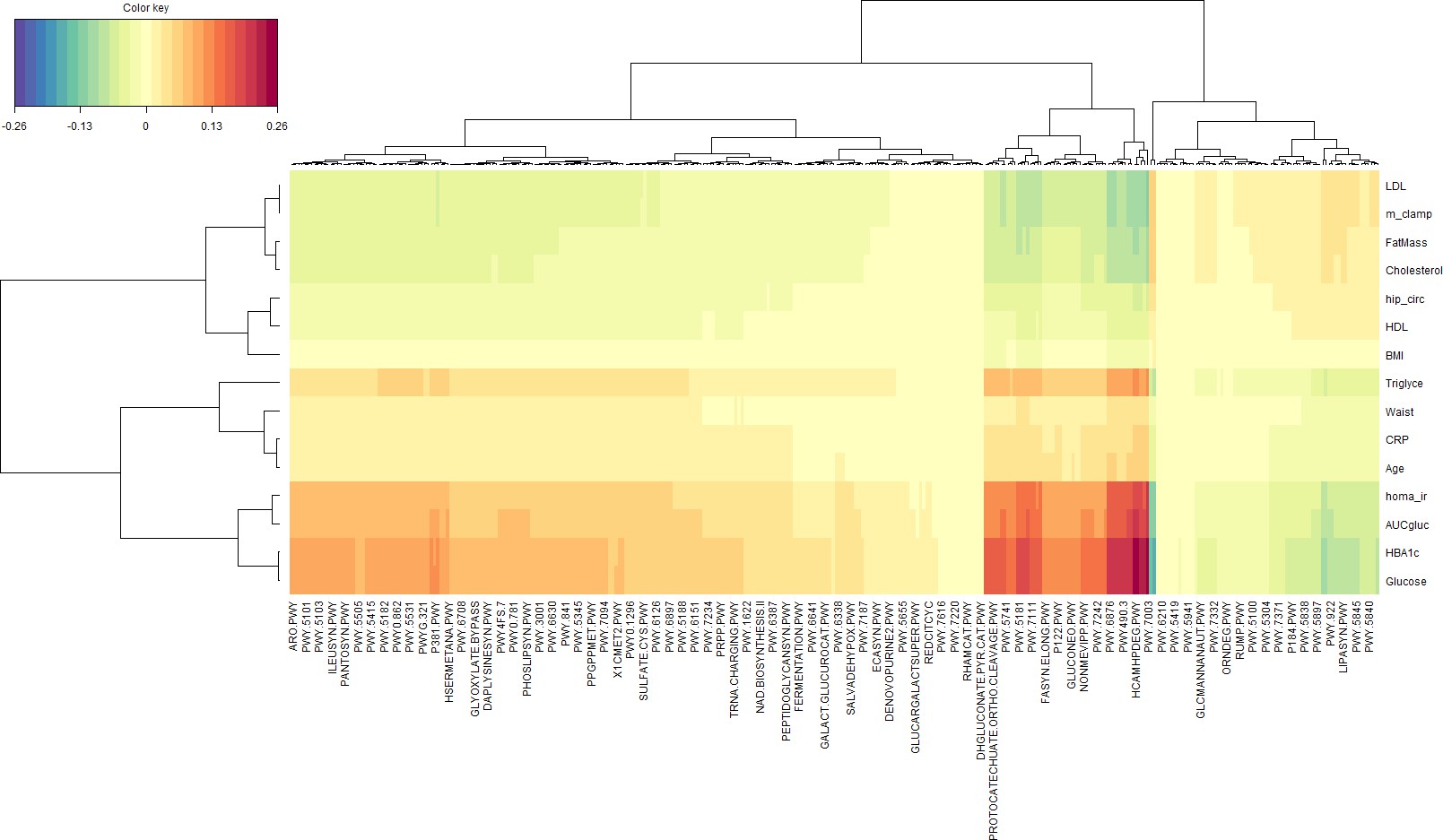


B Visceral component 1


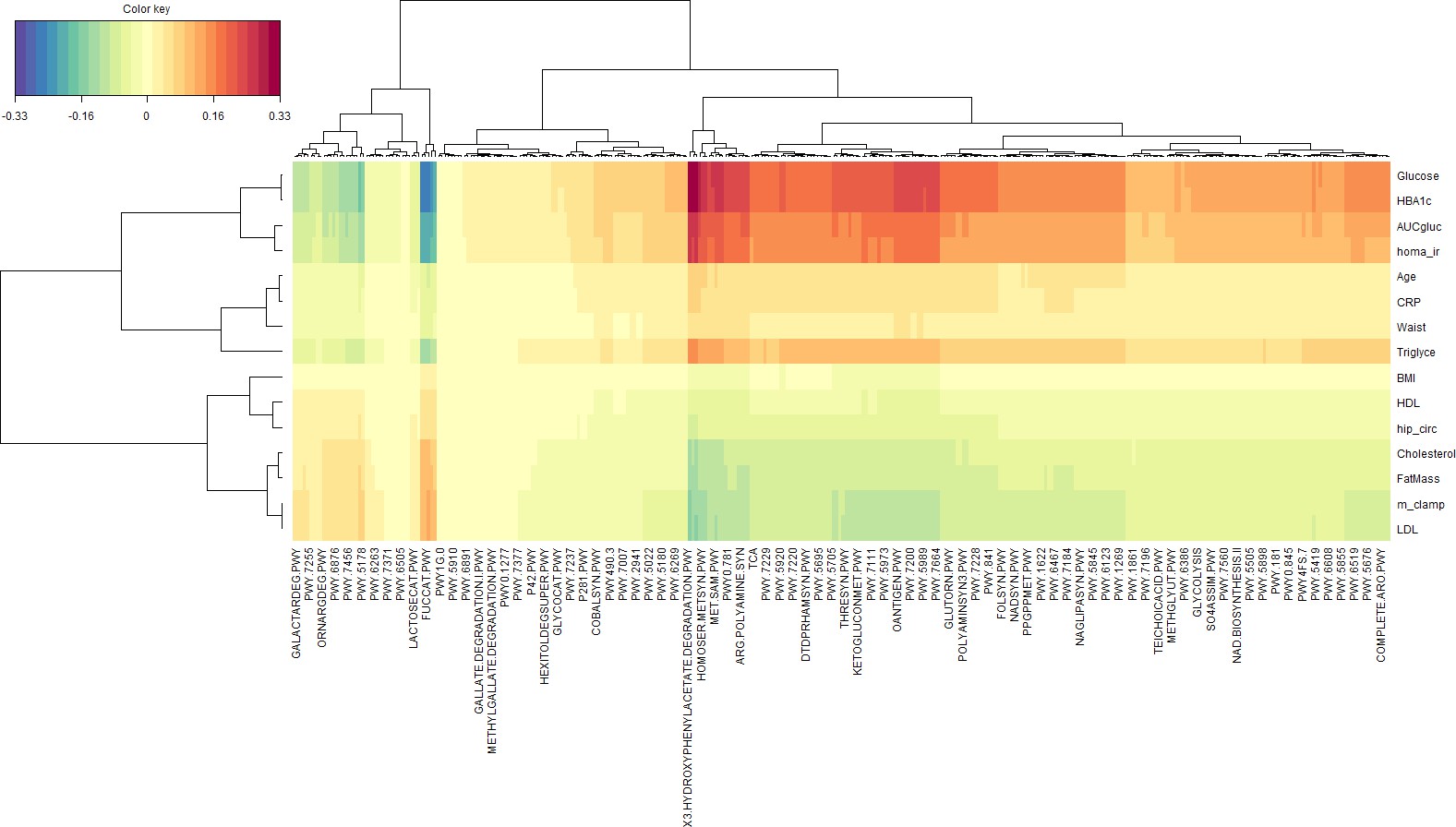


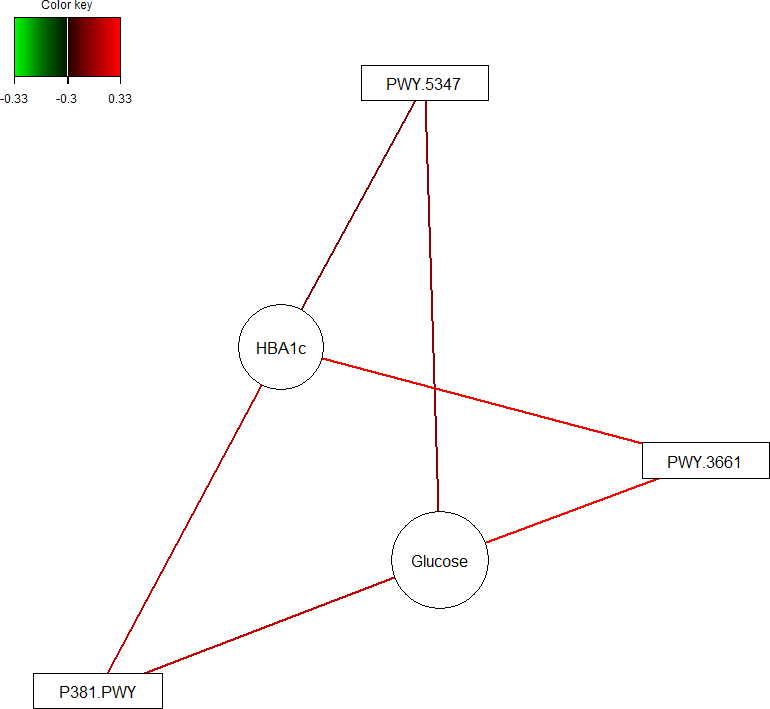

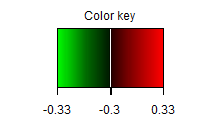

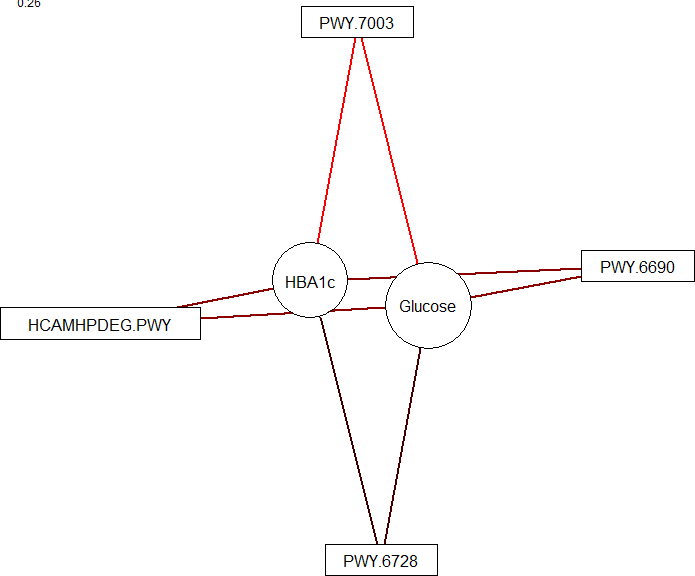

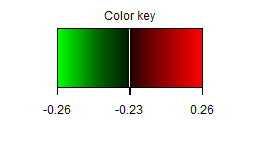


**C**

**D**

| Subcutaneous | Component 1 |
| --- | --- |
| PWY-7003 | glycerol degradation to butanol |
| PWY-6690 | cinnamate and 3- hydroxycinnamate degradation to 2-hydroxypentadienoate |
| HCAMHPDEG-PWY | 3-phenylpropanoate and 3-(3- hydroxyphenyl)propanoate degradation to 2- hydroxypentadienoate |
| PWY-6728 | methylaspartate cycle |

| Visceral | Component 1 |
| --- | --- |
| PWY-3661 | glycine betaine degradation I |
| P381-PWY | adenosylcobalamin biosynthesis II  (aerobic) |
| PWY-5347 | superpathway of L-methionine biosynthesis (transsulfuration) |


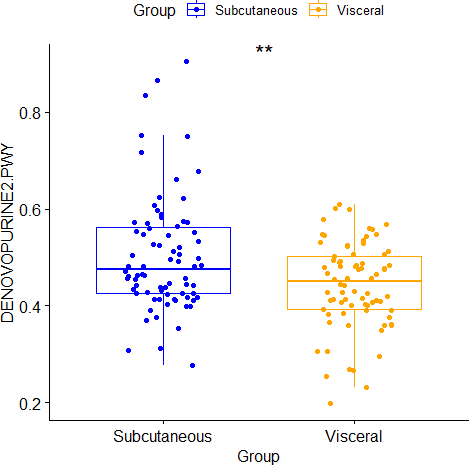
Wilcoxon-test


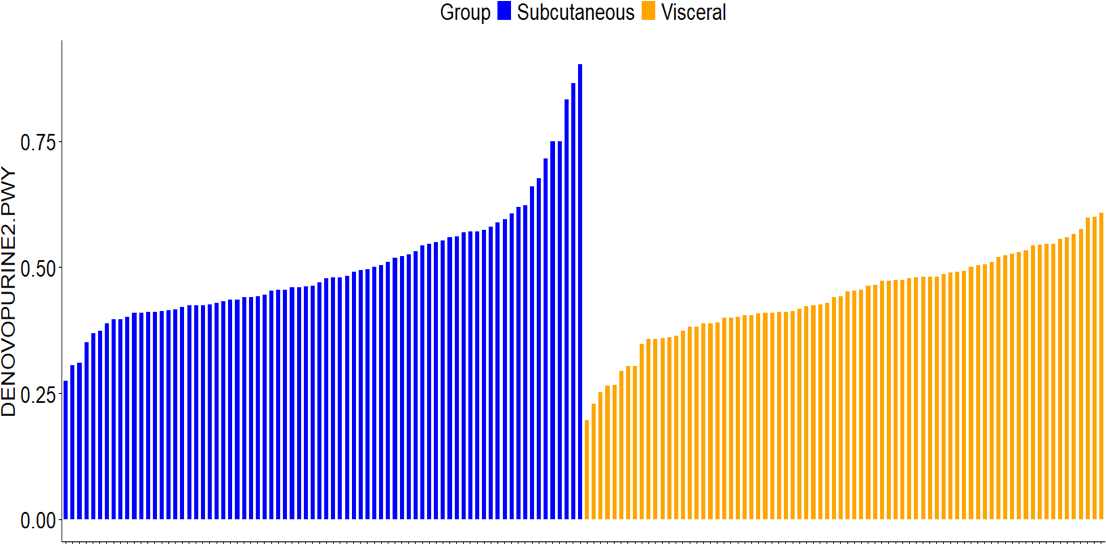


**A**

**B**

**C D**


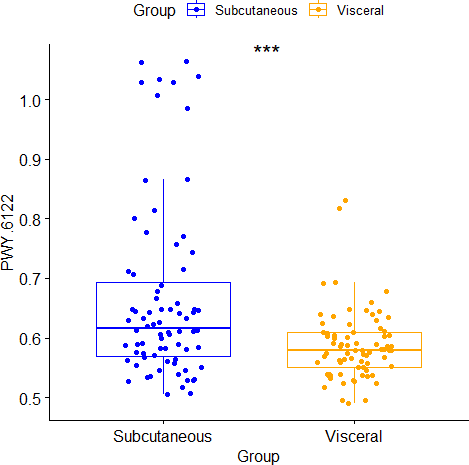
Wilcoxon-test


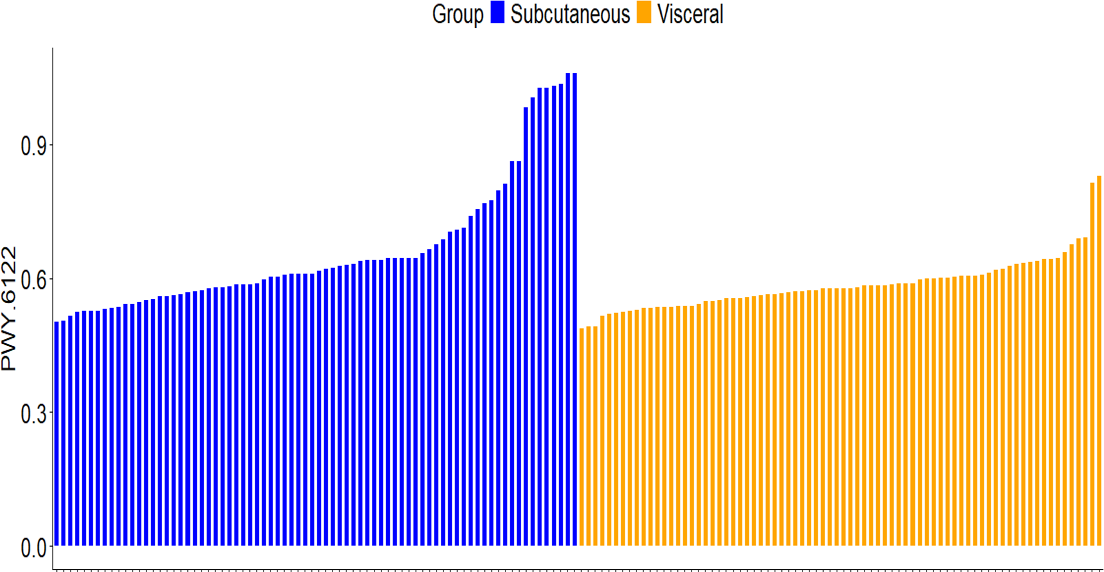


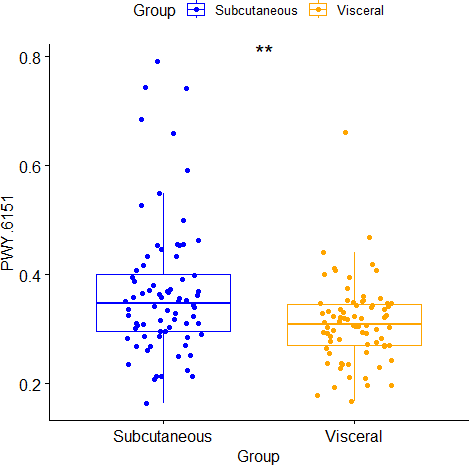
Wilcoxon-test

**E F**


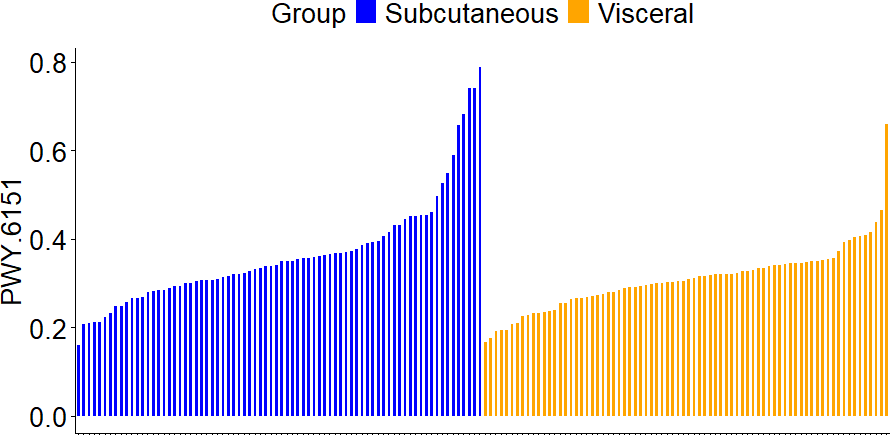


**G**


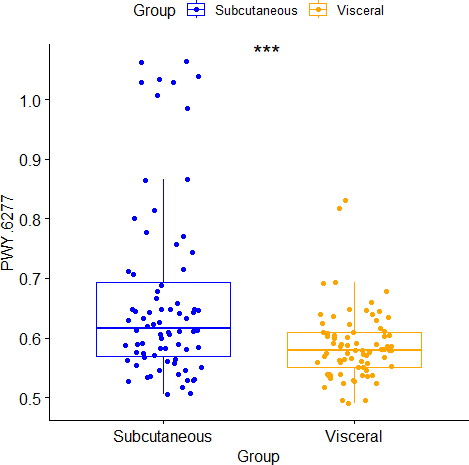


Wilcoxon-test

**H**


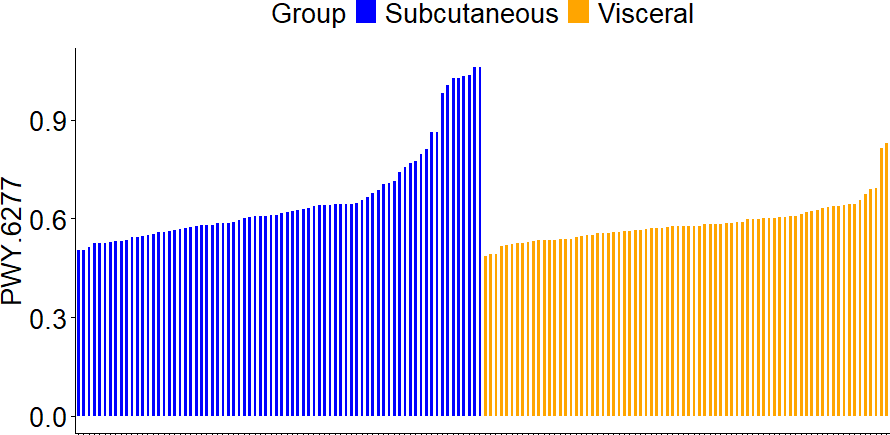


**
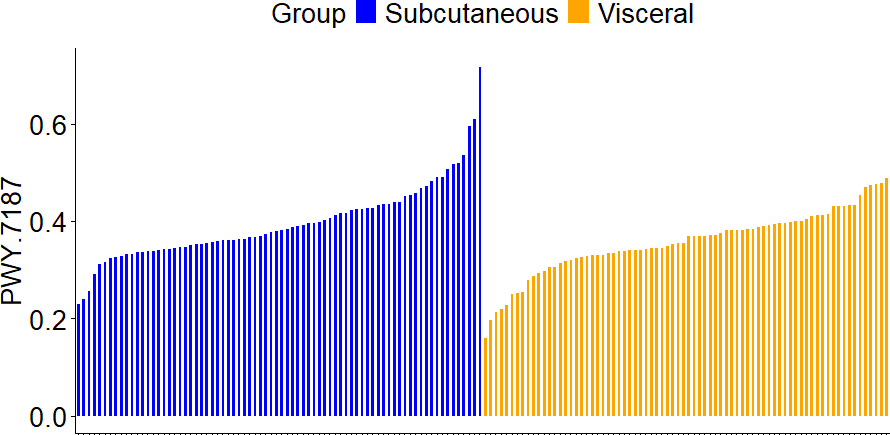
****I**


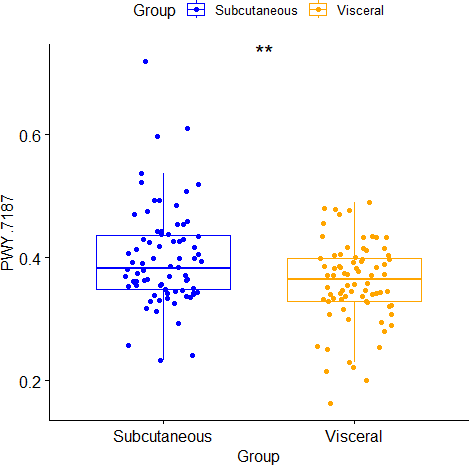


**J**

Wilcoxon-test


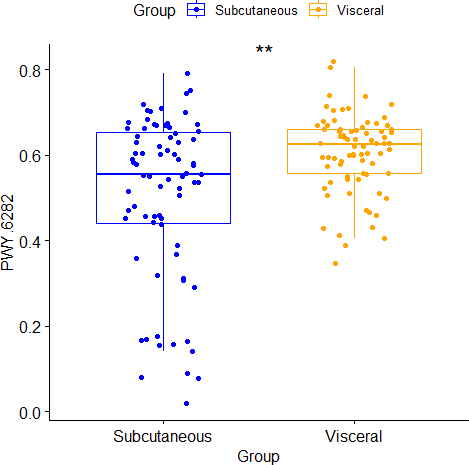
**K L** Wilcoxon-test


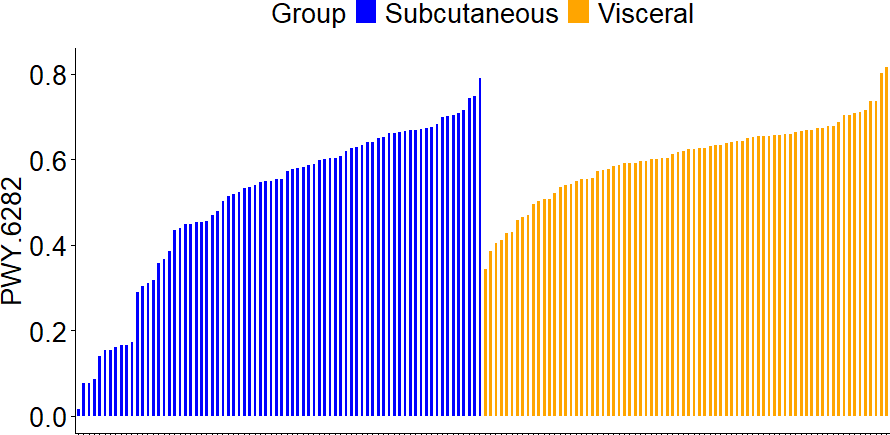


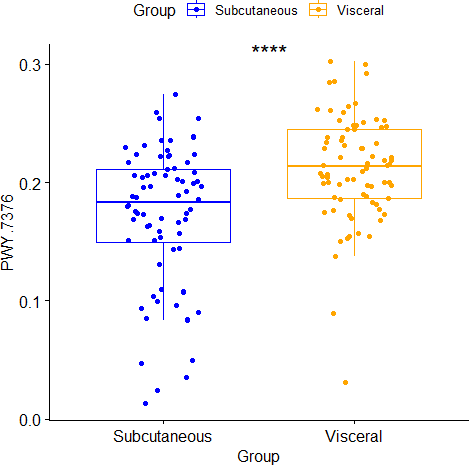
**M N** Wilcoxon-test

**N**


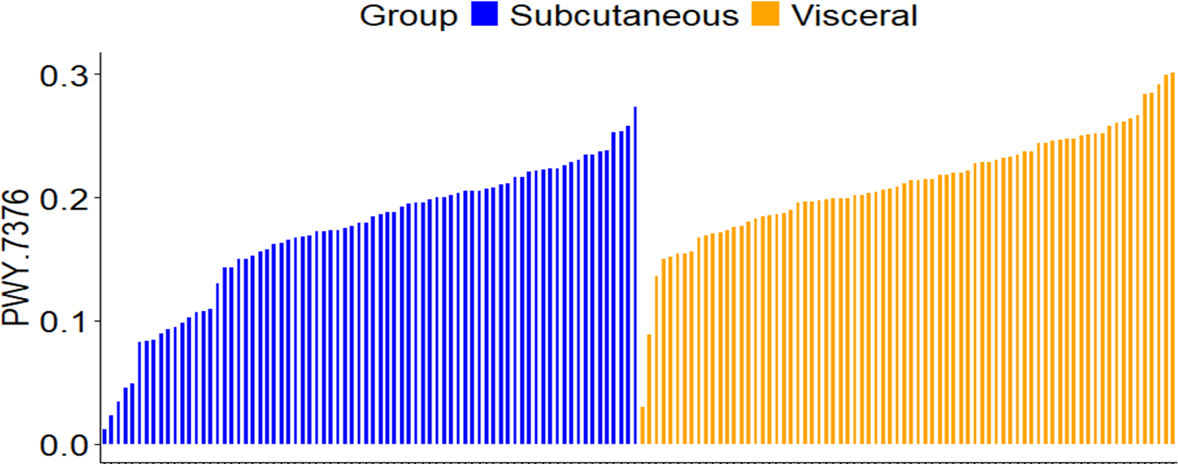


**O**


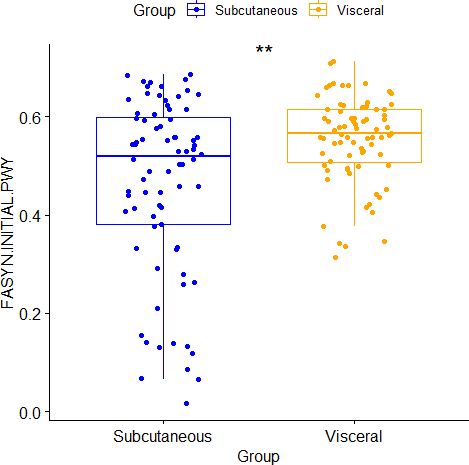


**P**

Wilcoxon-test


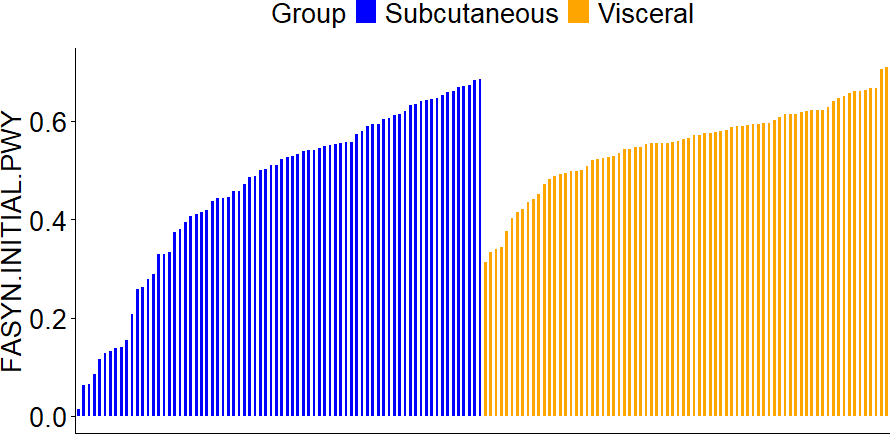


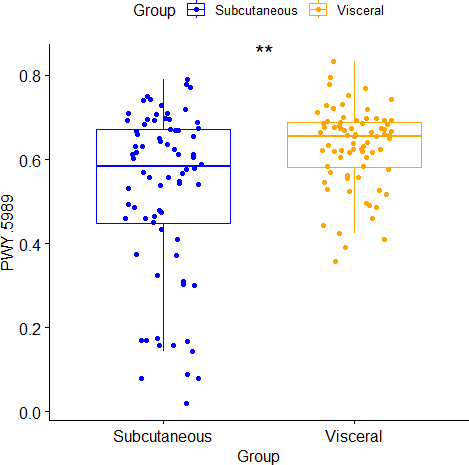
**R** Wilcoxon-test

**R**


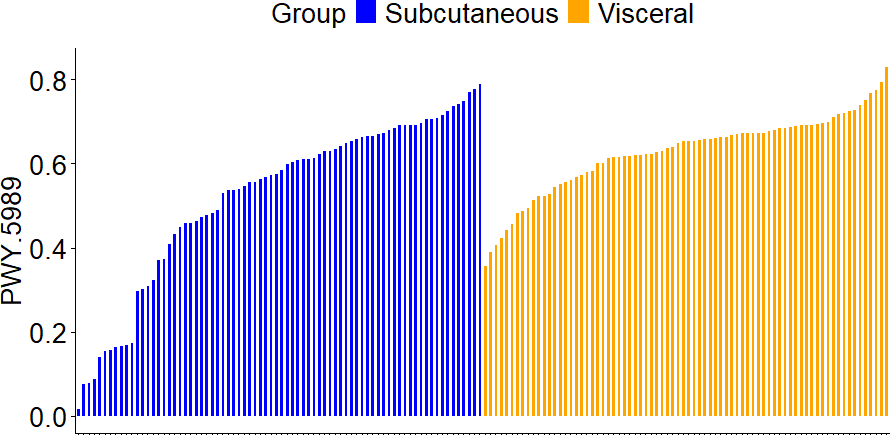


**Q**

**S**


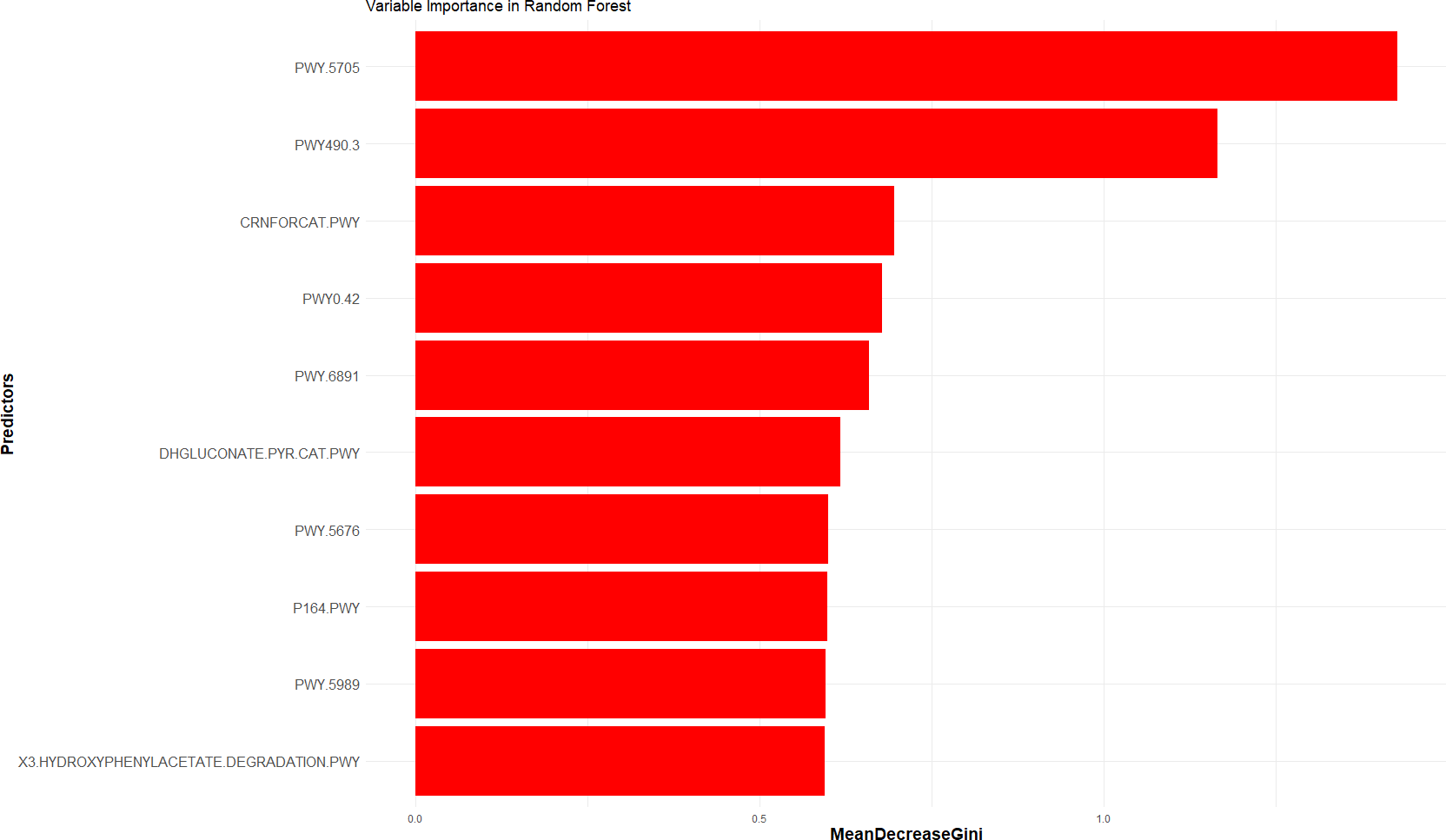


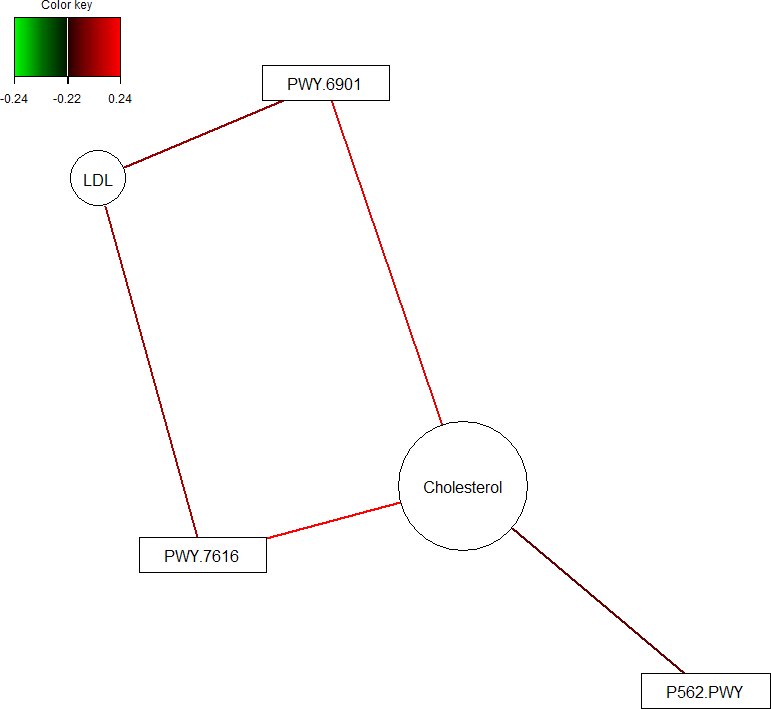

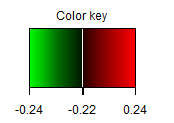

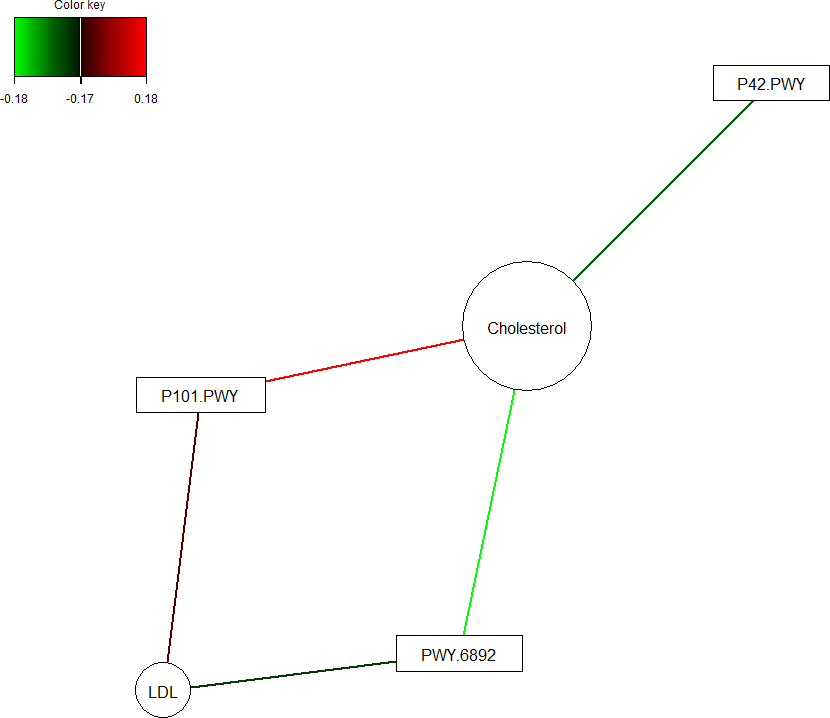

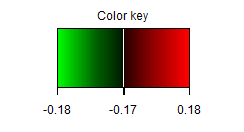


**A**

| Subcutaneous | Component 2 |
| --- | --- |
| P42.PWY | Incomplete reductive TCA cycle |
| P101-PWY | ectoine biosynthesis |
| PWY-6829 | tRNA methylation (yeast) |

**B**

| Visceral | Component 2 |
| --- | --- |
| PWY-6901 | superpathway of glucose  and xylose degradation |
| P562-PWY | *myo*-inositol degradation I |
| PWY-7616 | methanol oxidation to  carbon dioxide |

A Discriminant analysis of OTUs in subcutaneous adipose tissue


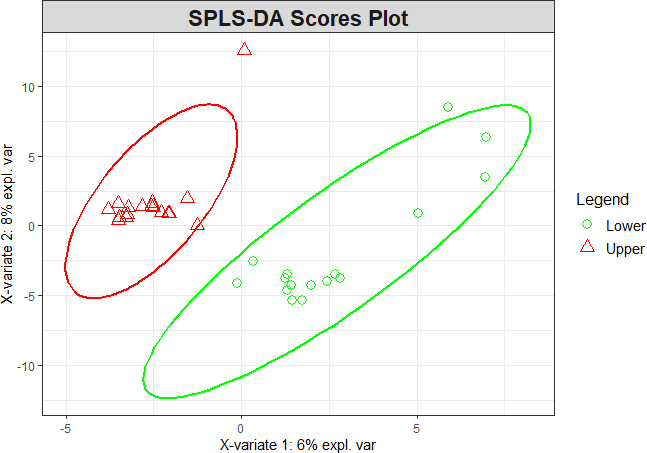


**C D**

B Discriminant analysis of OTUs in visceral

adipose tissue


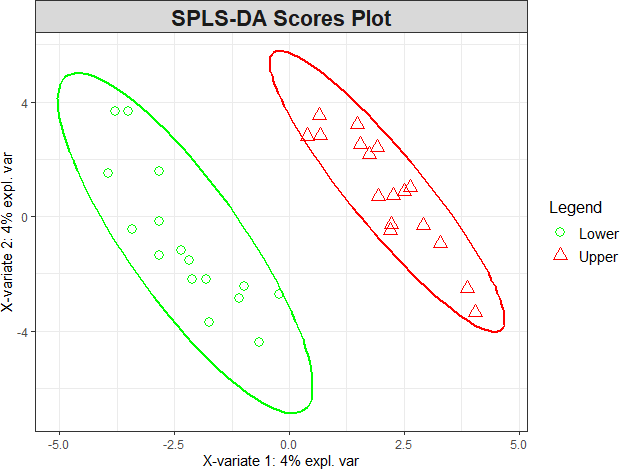


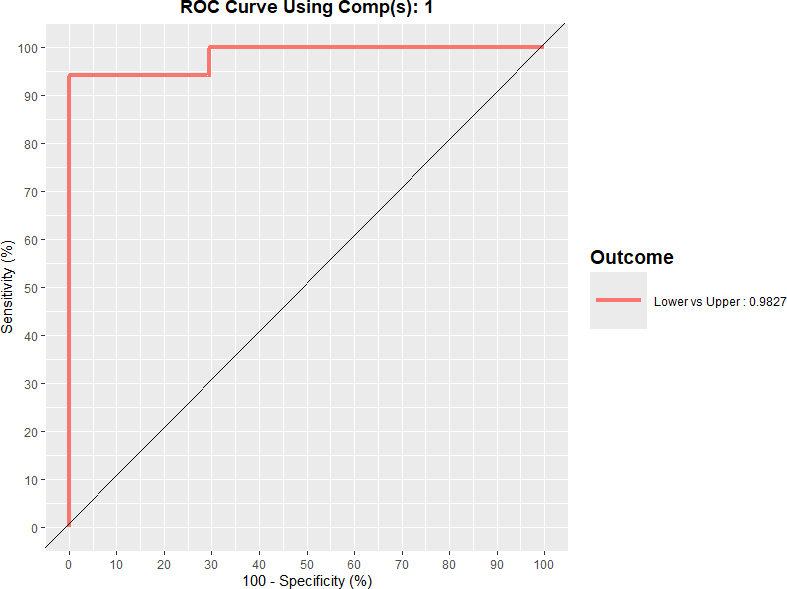

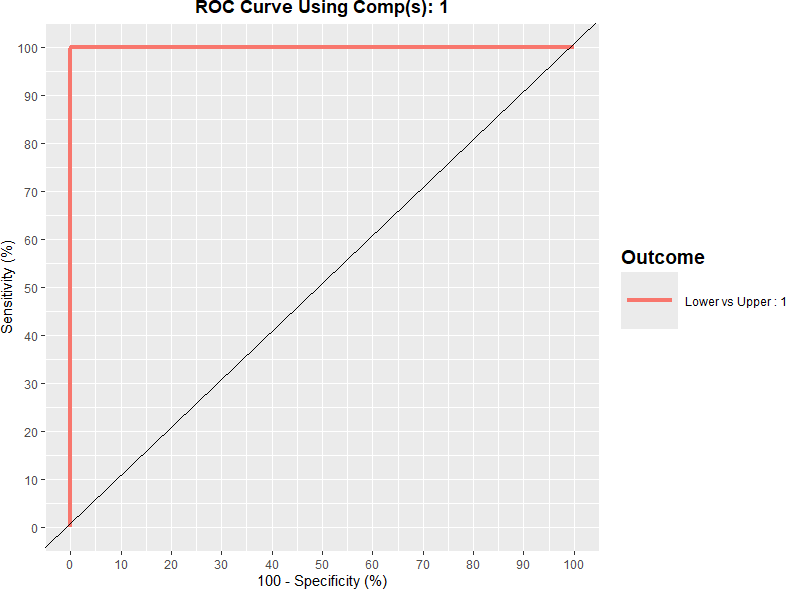


## Subcutaneous

**
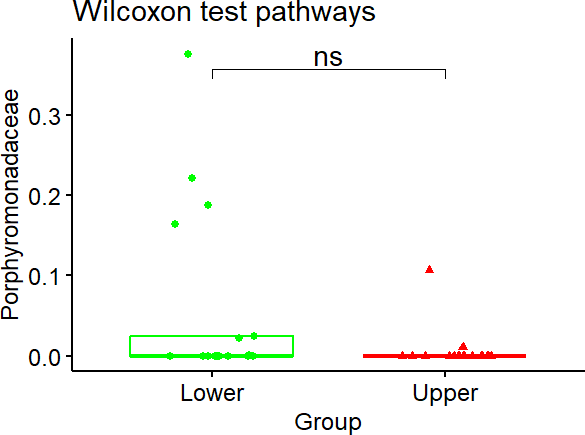
E F**


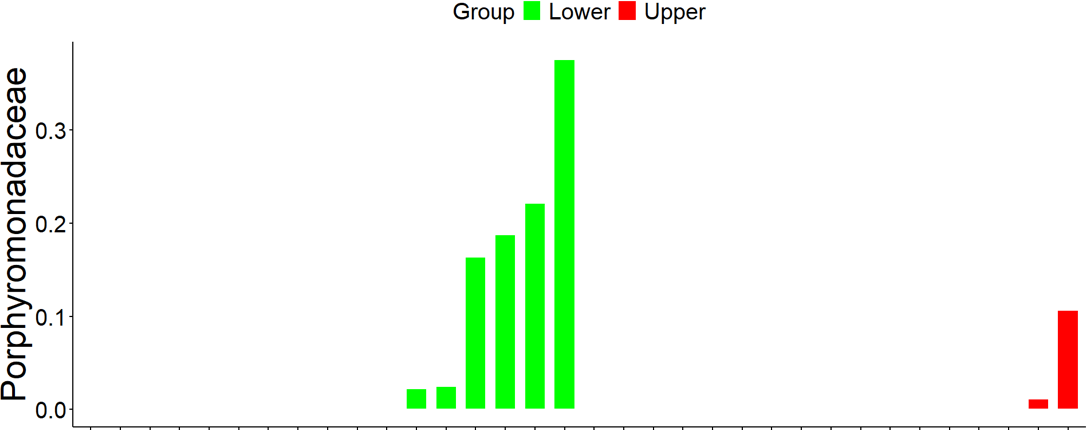


**
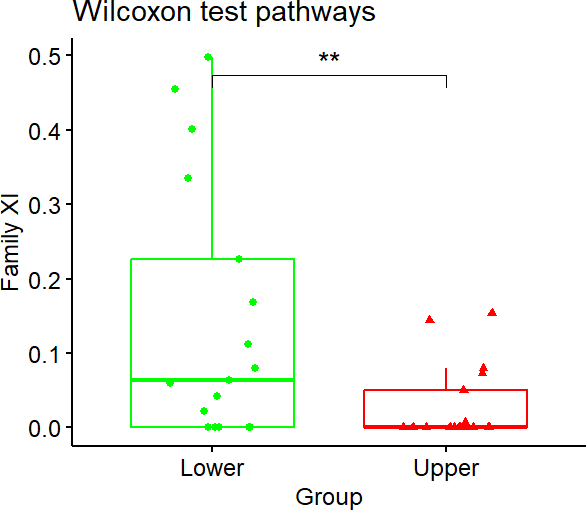
G H**


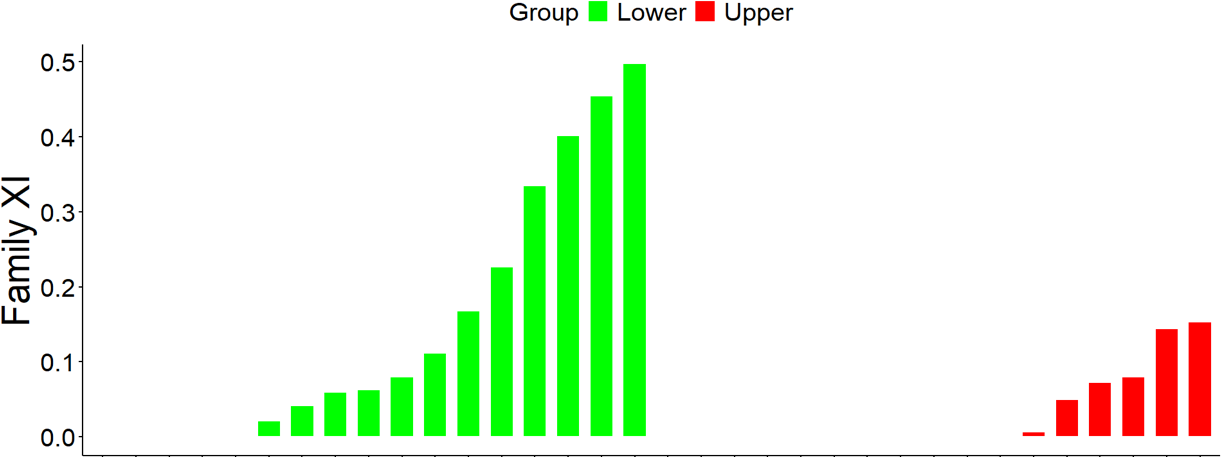


## Subcutaneous

**
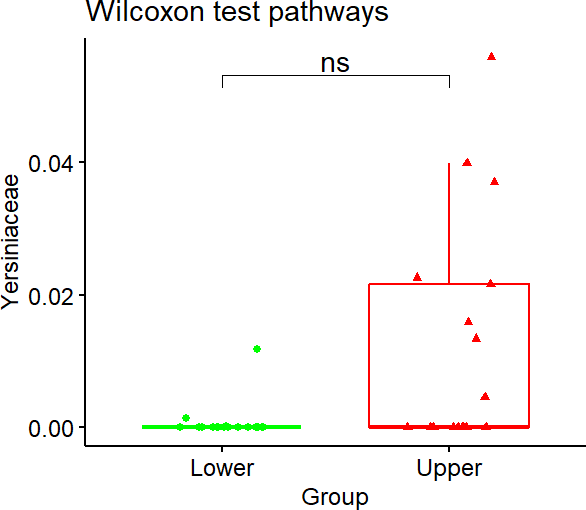
I J**


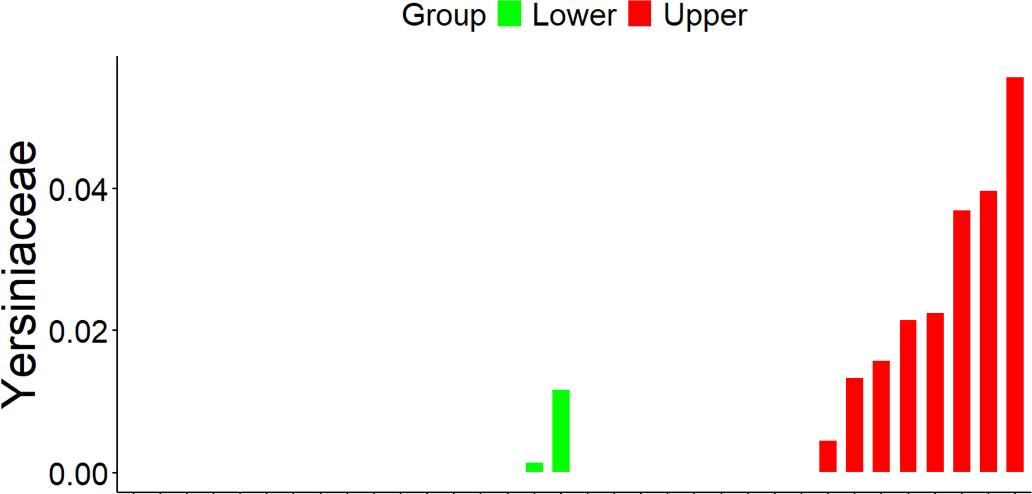


**
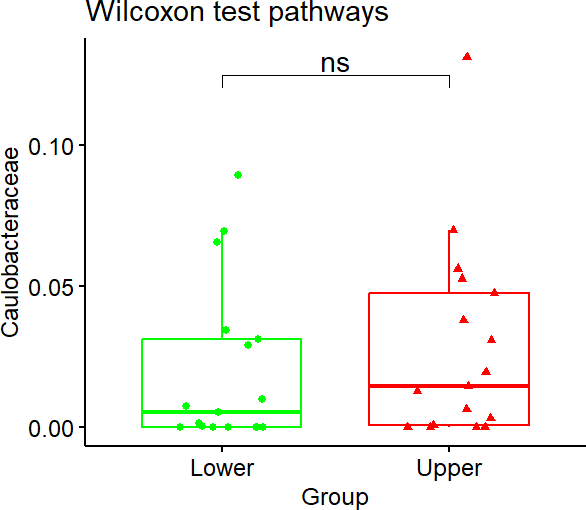
K L**


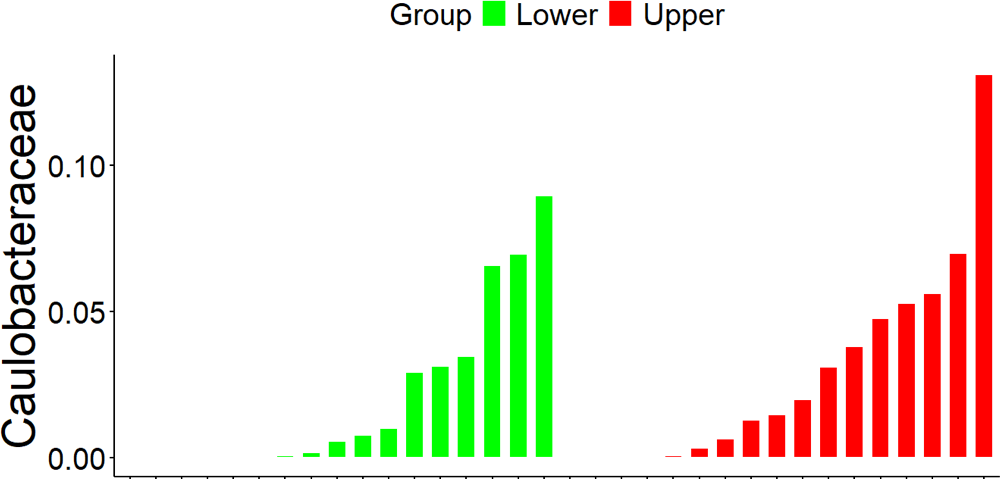


## Subcutaneous

**
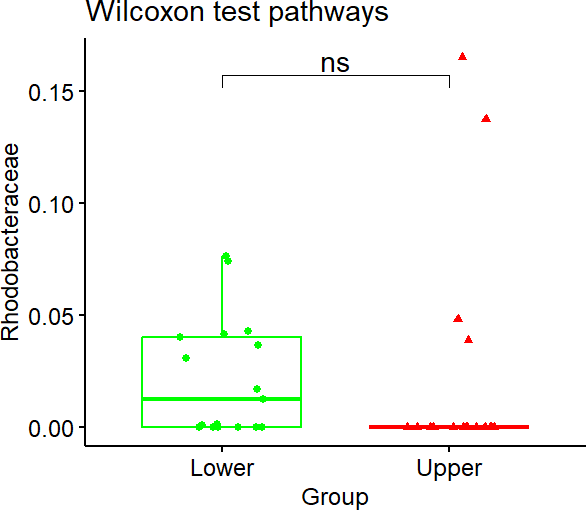
N**


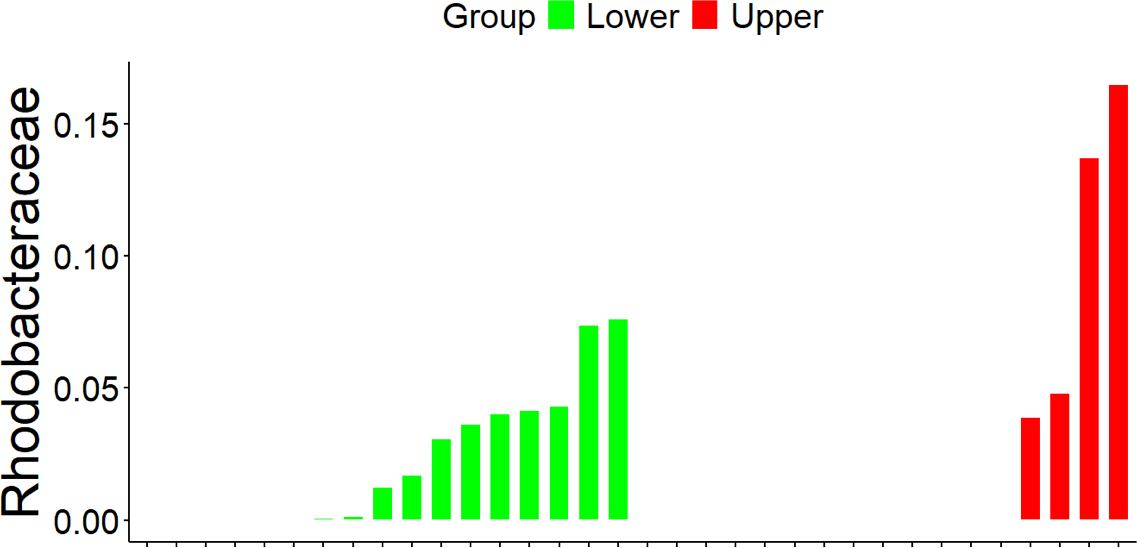


**M**

**
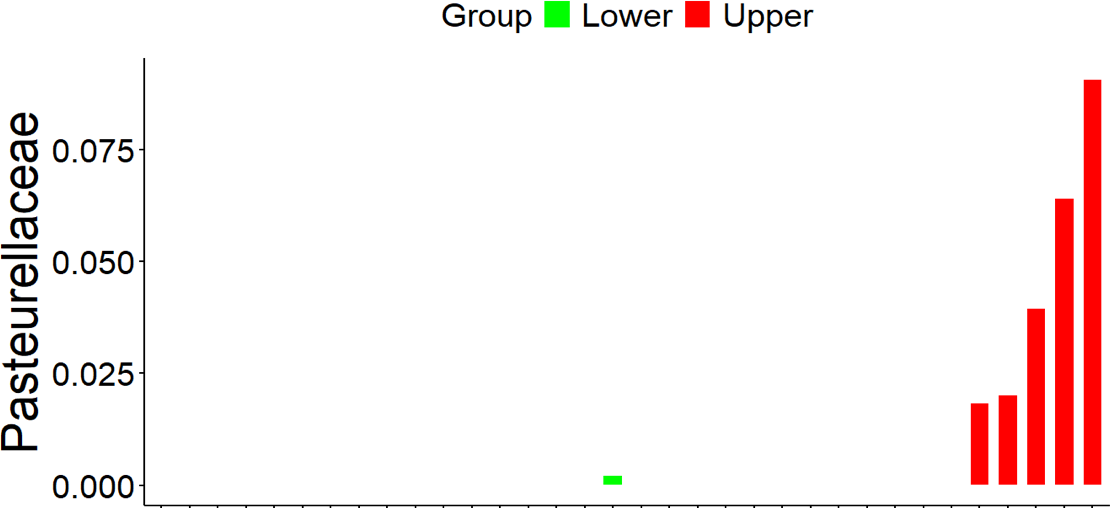
O**


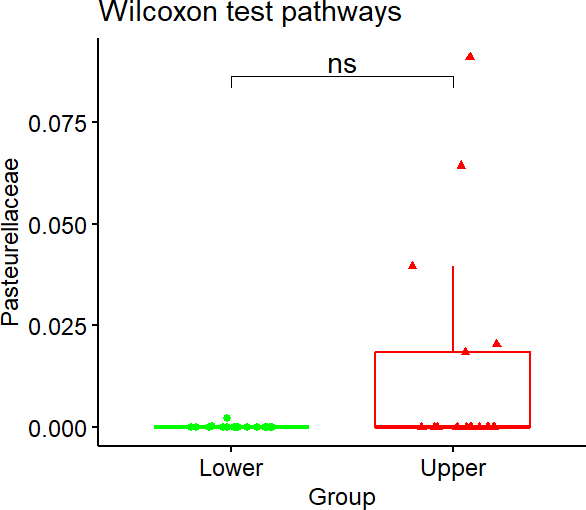


**P**

## Visceral

**
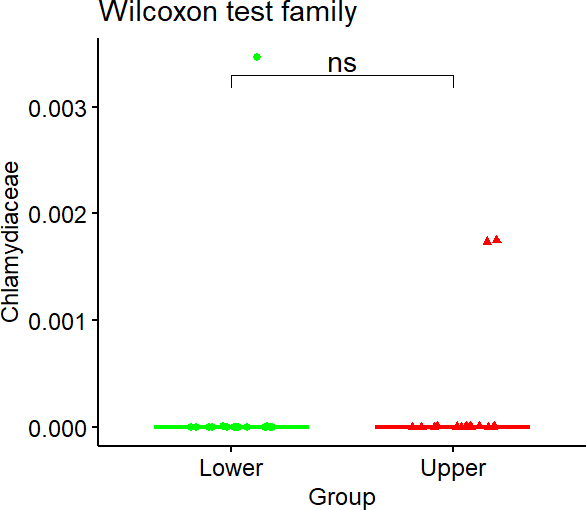
Q R**


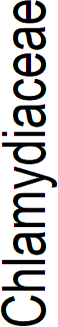

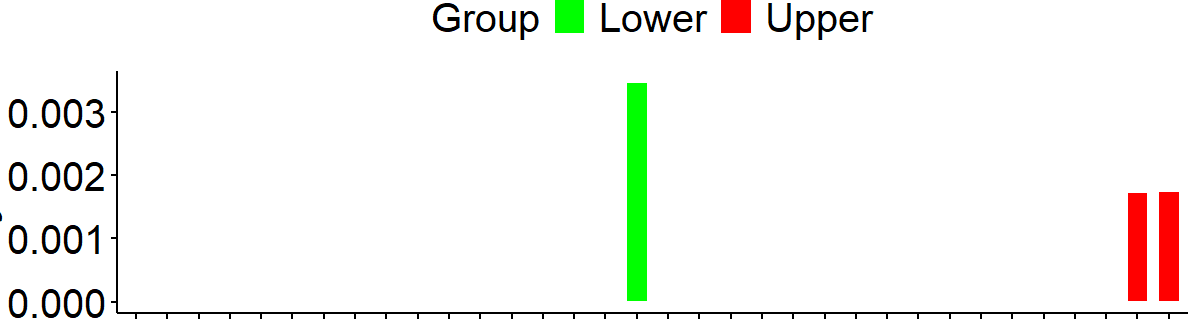


**T**

**
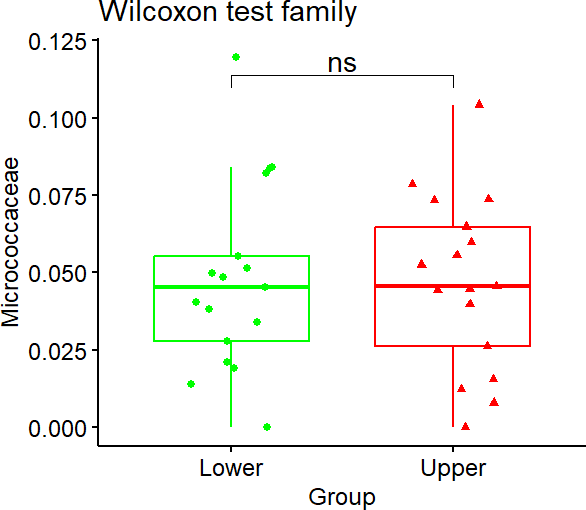
S**


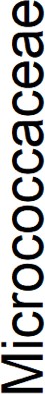

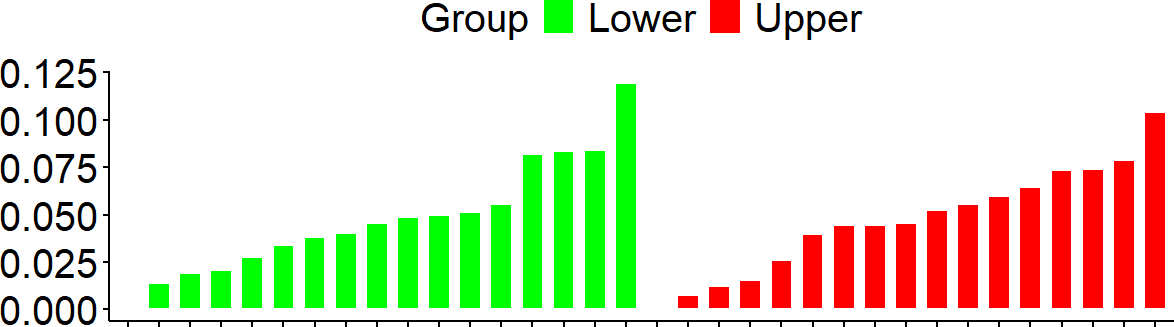


## Visceral

**
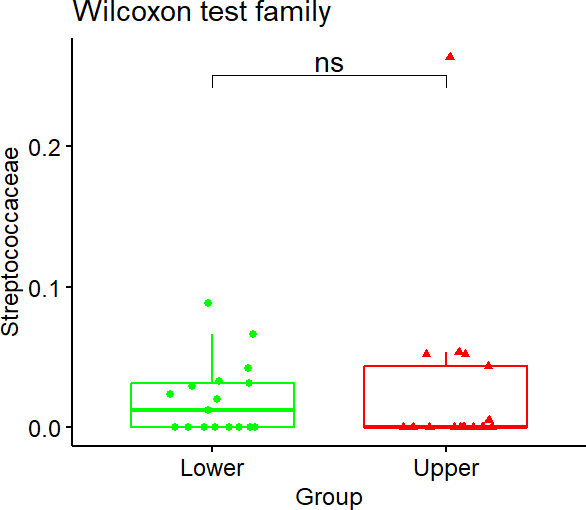
U V**


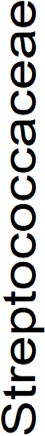

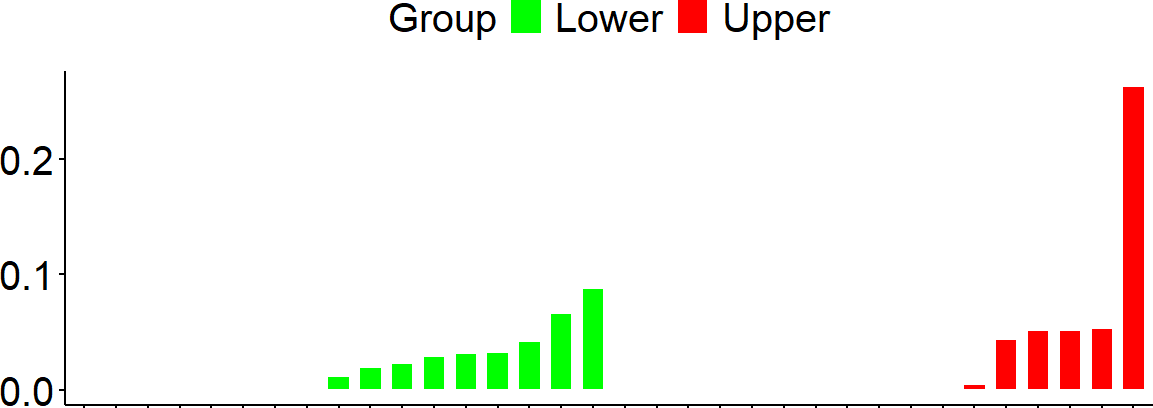


**
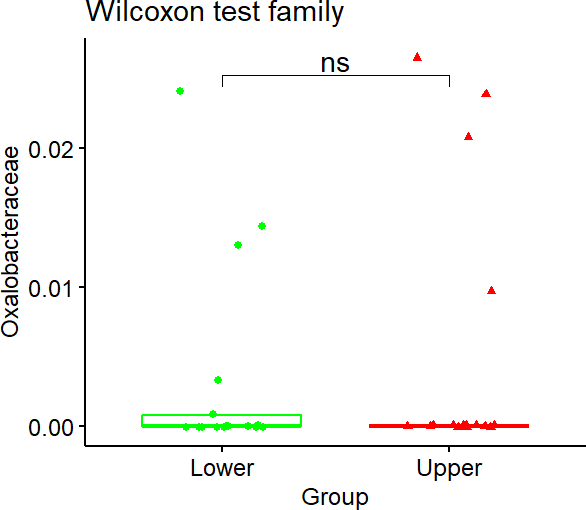
X**

**W**


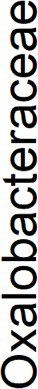

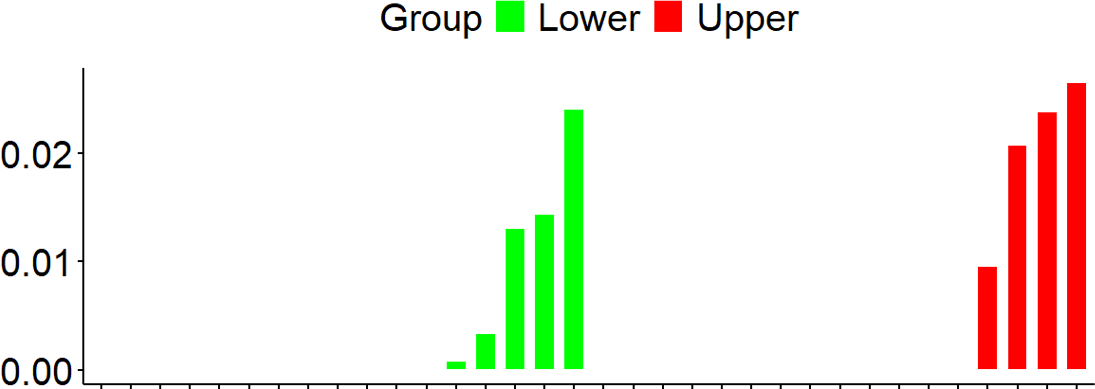


1. **Metabolic pathways in the subcutaneous adipose tissue**
2. **Metabolic pathways in the visceral adipose tissue**


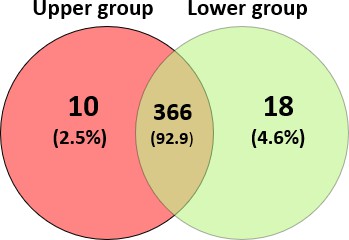

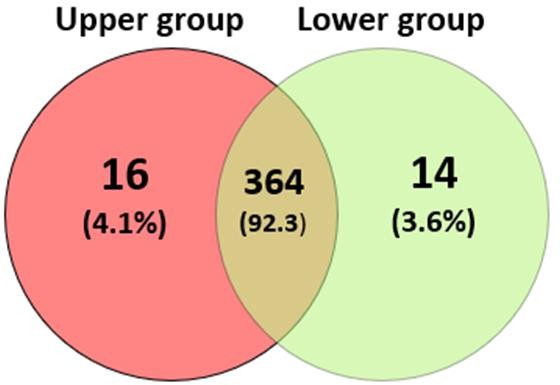


Ven Diagram Ven Diagram


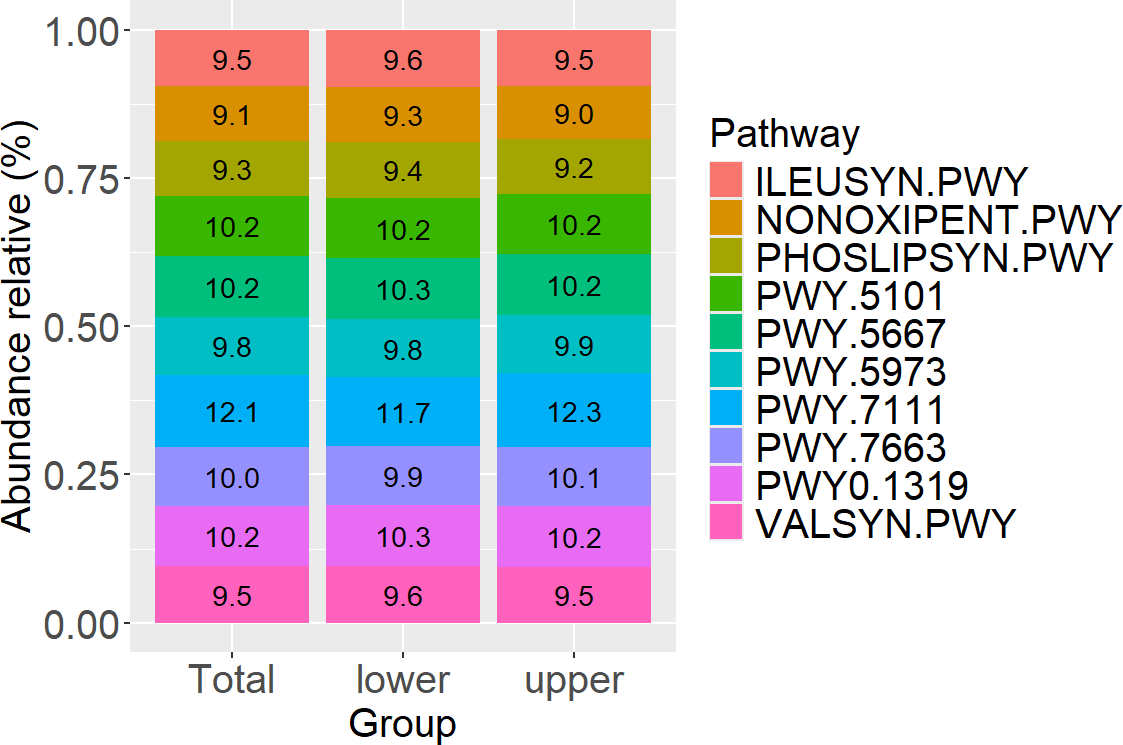

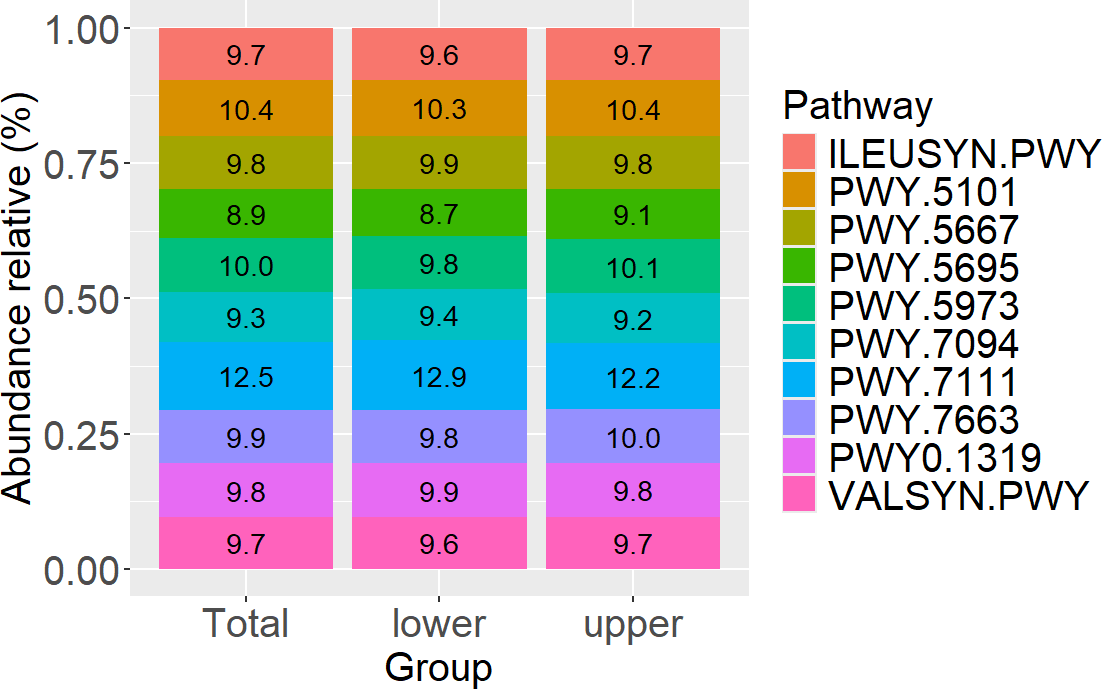


C PCA Subcutaneous adipose tissue D PCA Visceral adipose tissue


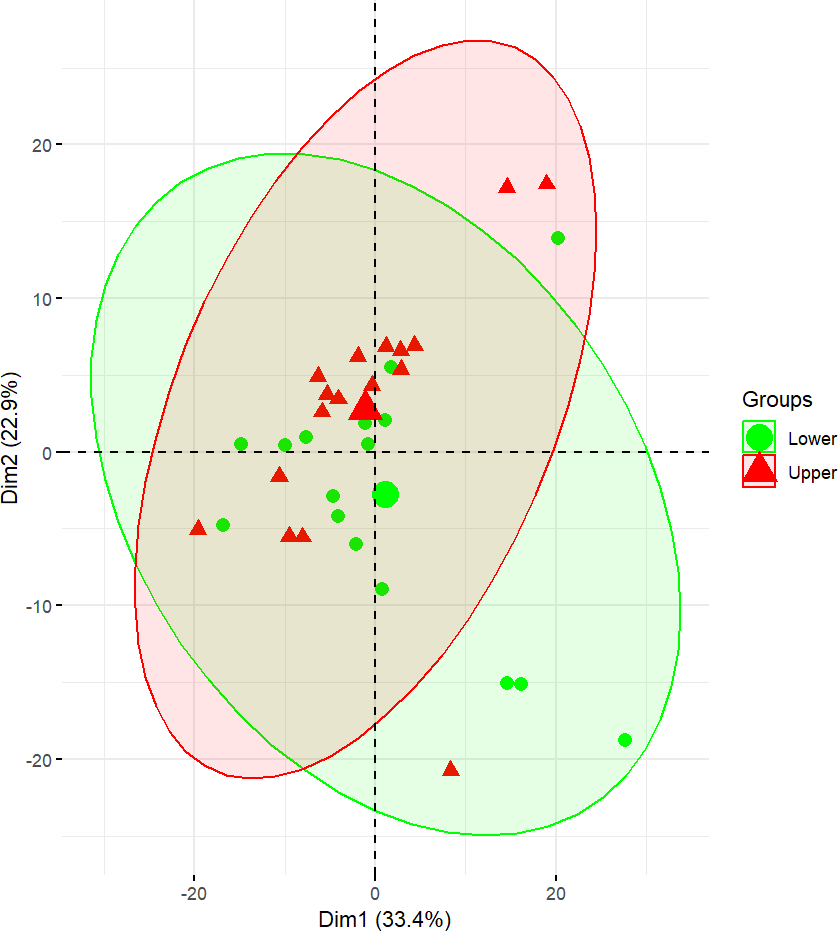

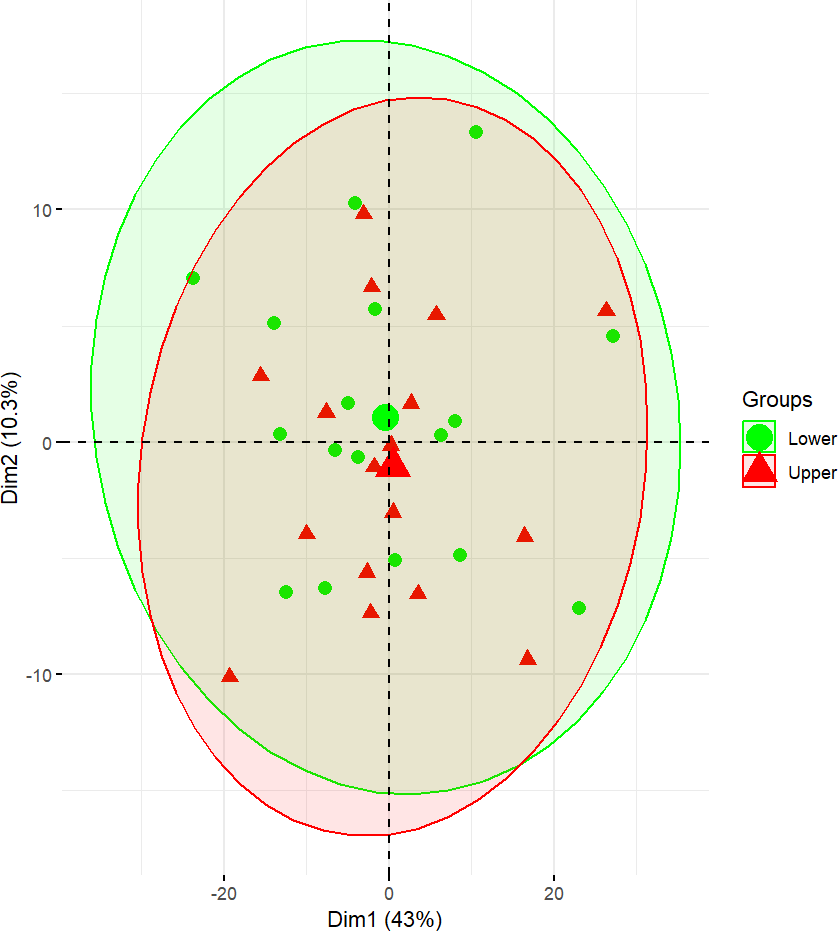


**E Heatmap of microbial metabolic pathways in subcutaneous adipose tissue**

## TWL Group


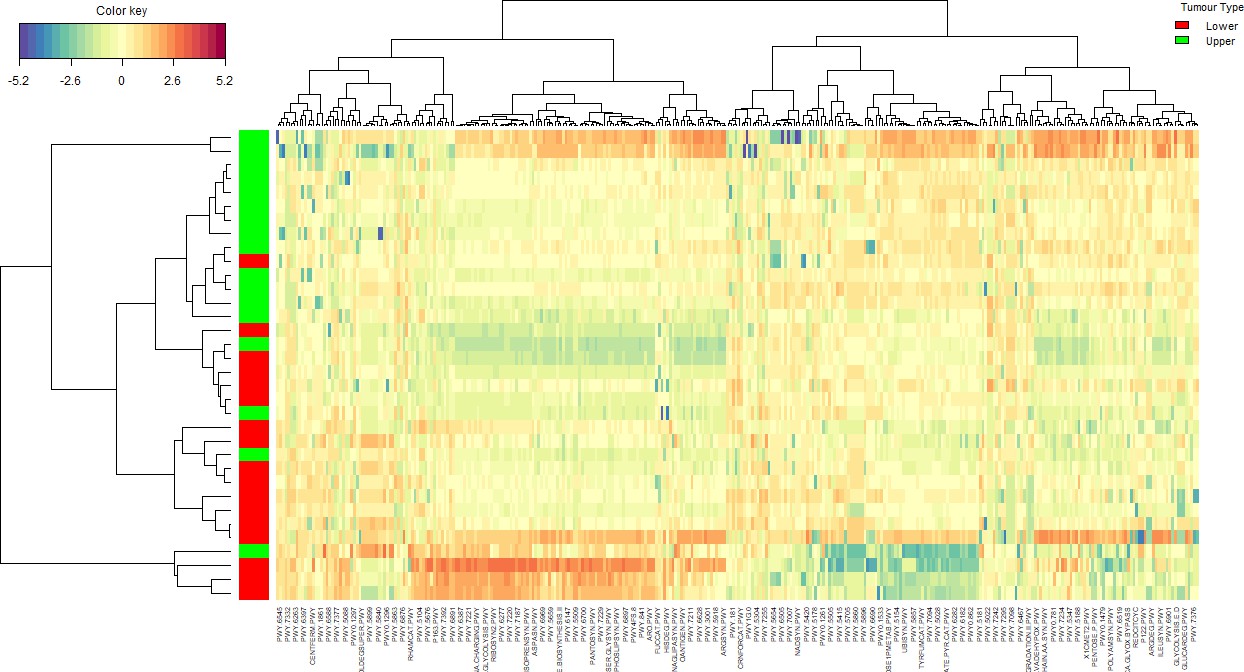

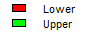


Subcutaneous adipose tissue

**
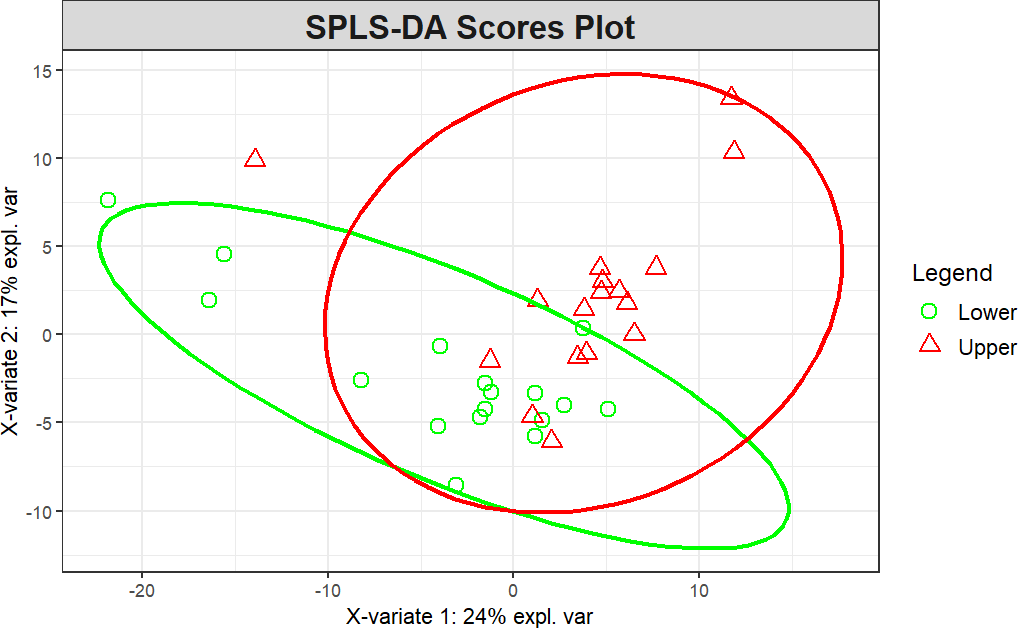
F G**


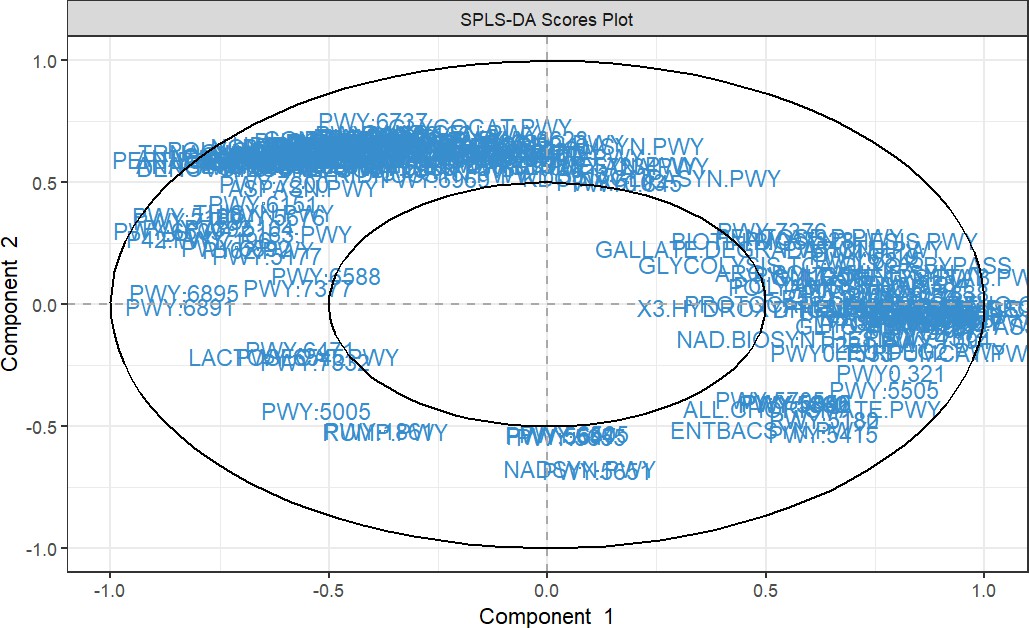


**H I**


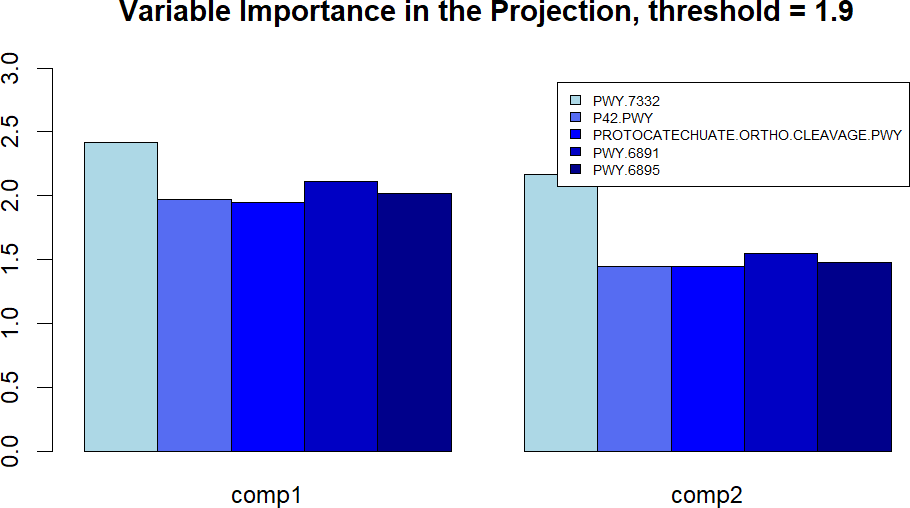


Component 1


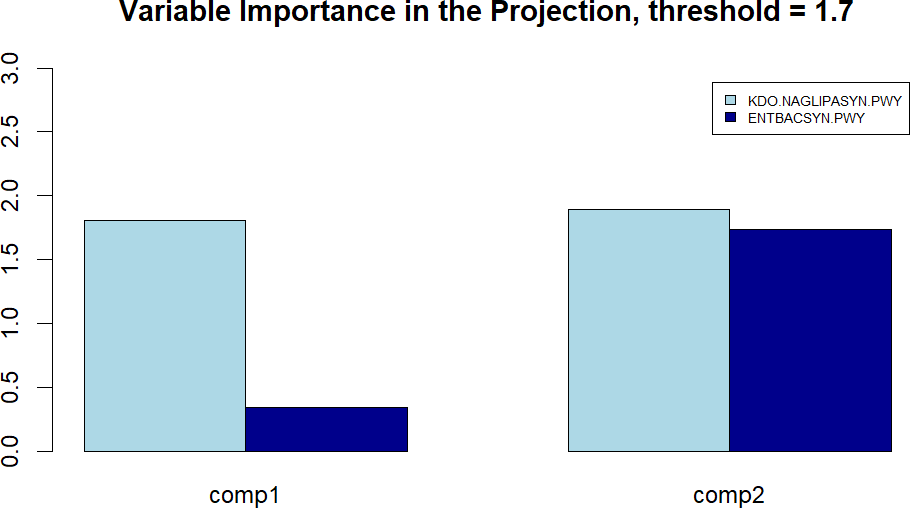


Component 2

J Subcutaneous adipose tissue

| **Pathways** | **Function** | **Lower (%)** | **Upper (%)** | **P-value Wilcoxon test** |
| --- | --- | --- | --- | --- |
| PWY-6895 | superpathway of thiamin  diphosphate biosynthesis II | 0.3±0.14 | 0.18±0.087 | 0.01449 |
| PWY-6891 | thiazole biosynthesis II (Bacillus) | 0.15±0.11 | 0.066±0.052 | 0.01959 |
| PWY-7332 | superpathway of UDP-N- acetylglucosamine- derived O-antigen building  blocks biosynthesis | 0.08±0.069 | 0.022 ±0.038 | 0.002793 |
| P42-PWY | incomplete reductive TCA  cycle | 0.56±0.26 | 0.36±0.12 | 0.008507 |
| KDO-NAGLIPASYN-PWY | superpathway of (Kdo)2-  lipid A biosynthesis | 0.043 ±0.02 | 0.061±0.068 | 0.01179 |
| ENTBACSYN-PWY | enterobactin biosynthesis | 0.13±0.058 | 0.12±0.066 | 0.8384 |

1. **Heatmap of microbial metabolic pathways in visceral adipose tissue**

## TWL Group


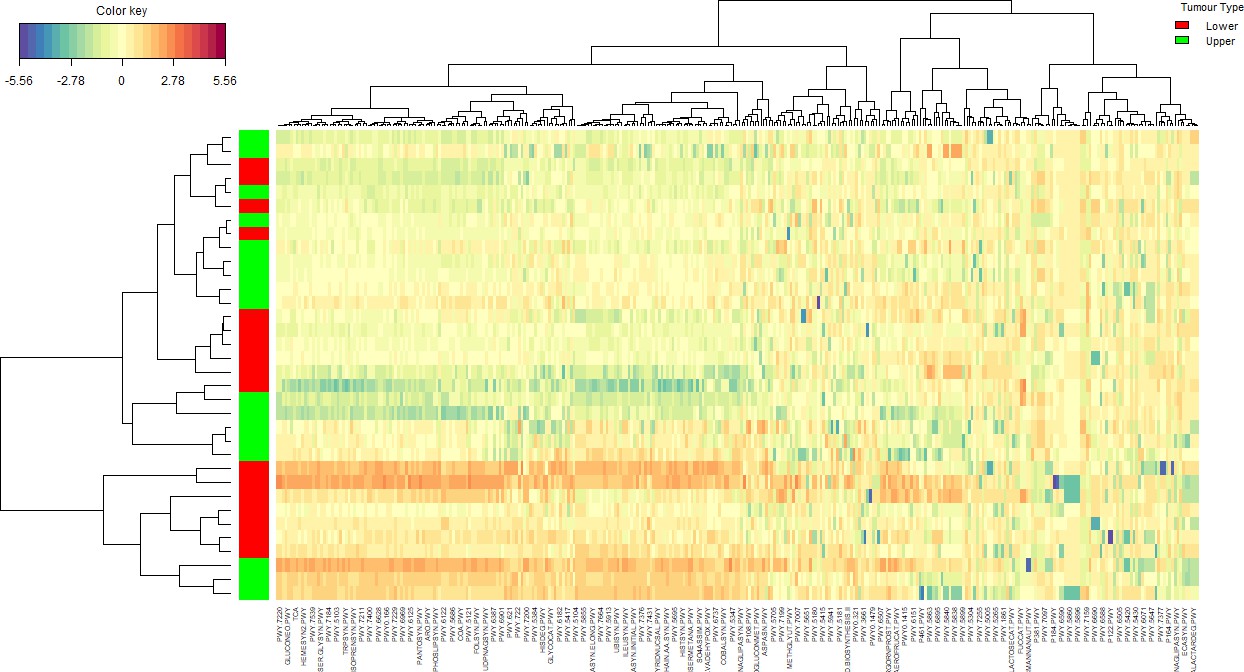

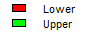


Visceral adipose tissue

1. **
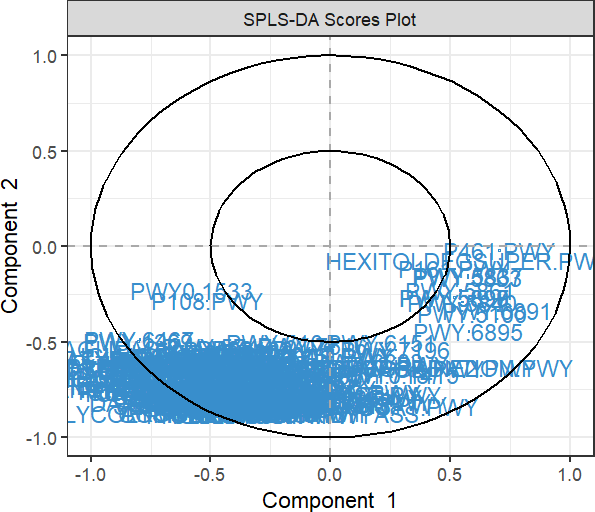

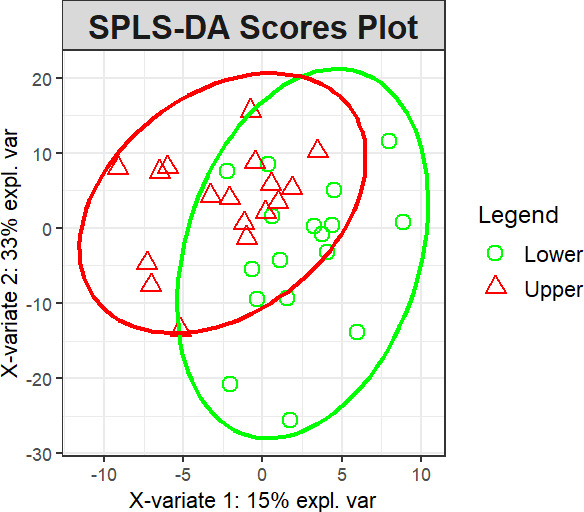
C**

**D E**

**H**


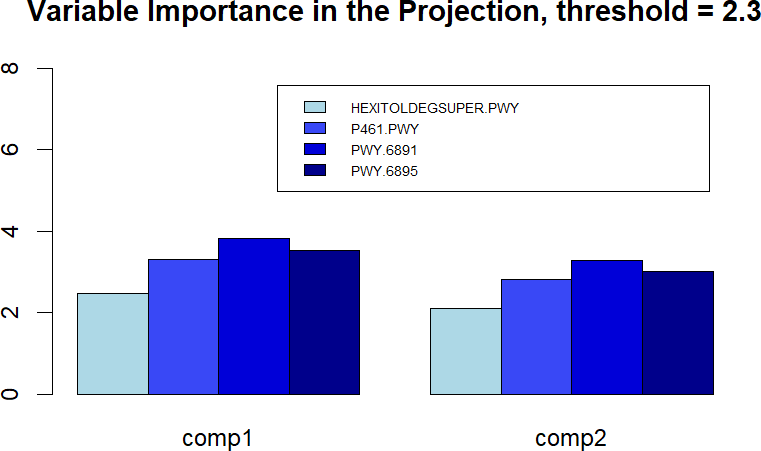


Component 1


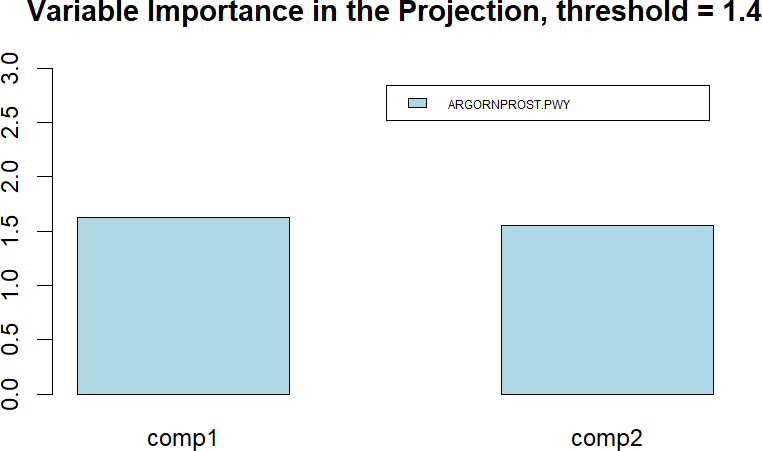


Component 2

Visceral adipose tissue

**F**

| **Pathways** | **Function** | **Lower (%)** | **Upper (%)** | **P-value**  **Wilcoxon test** |
| --- | --- | --- | --- | --- |
| P461-PWY | hexitol fermentation to lactate, formate, ethanol and acetate | 0.062±0.034 | 0.034±0.027 | 0.013 |
| PWY-6891 | thiazole biosynthesis II (Bacillus) | 0.098±0.059 | 0.046±0.025 | 0.0008 |
| PWY-6895 | superpathway of thiamin diphosphate biosynthesis II | 0.15±0.066 | 0.0024±0.065 | 0.001 |

##
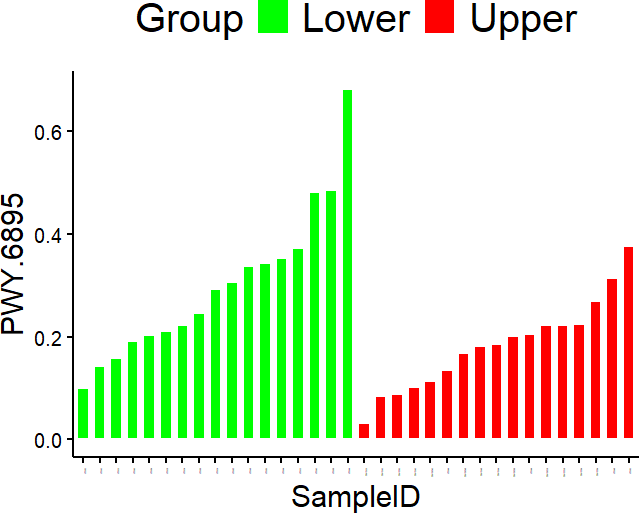
Subcutaneous

**
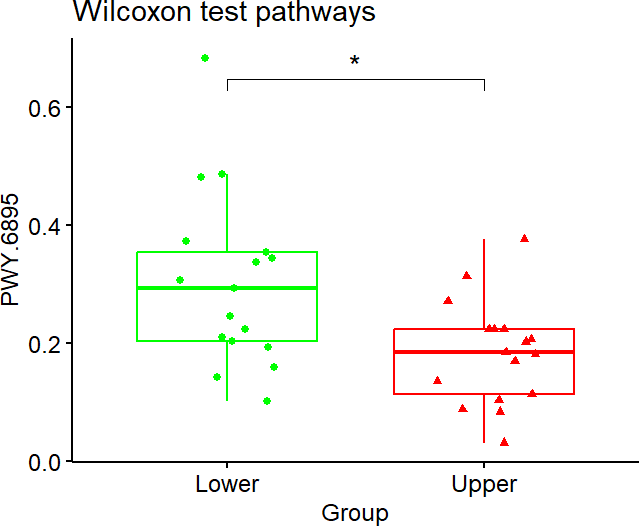
A B**

**C D**


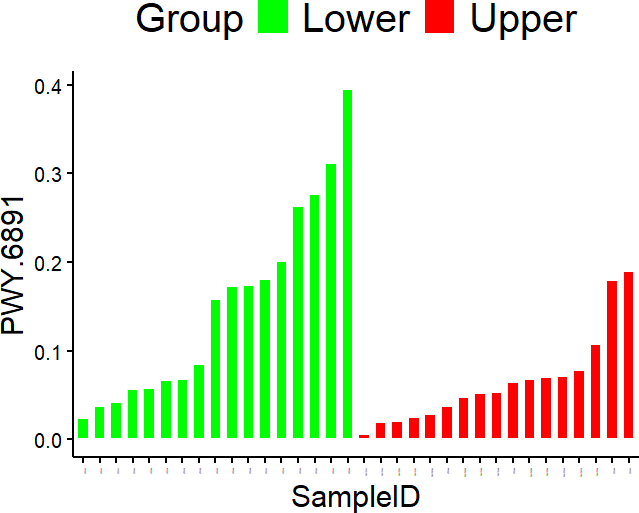

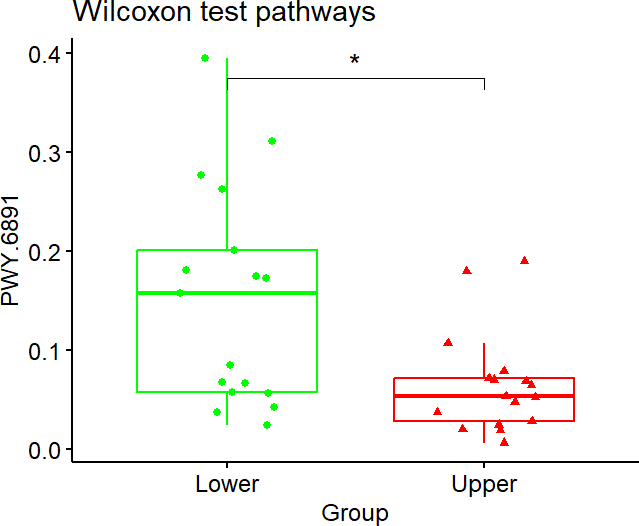


## Subcutaneous

**
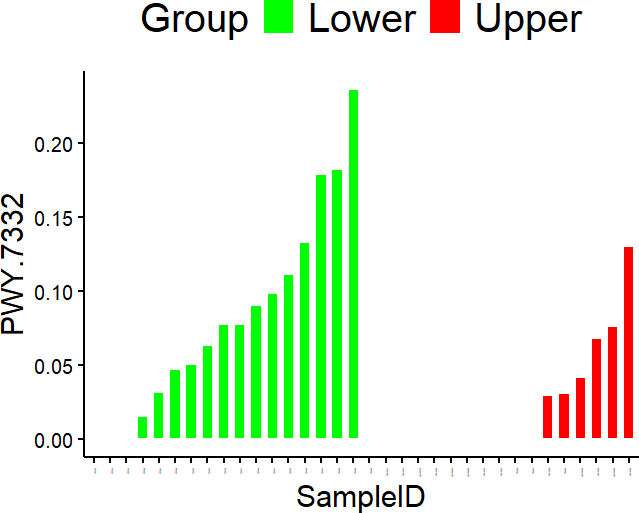

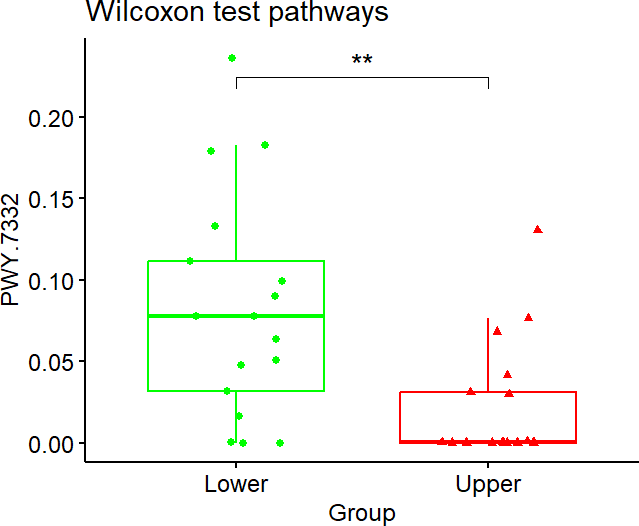
E F**

**
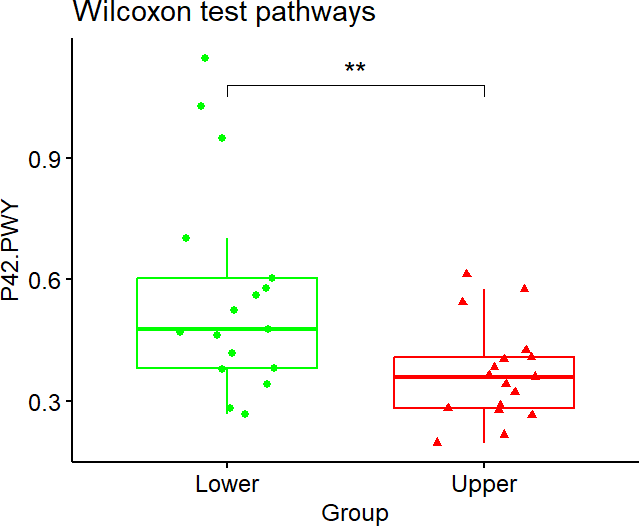
G H**

## Subcutaneous

**I J**

**K L**

## Subcutaneous

**M N**

**O P**

**Q R**

S Discriminant analysis sPLS-DA of microbial metabolic pathways in subcutaneous adipose tissue

**A Discriminant analysis of microbial metabolic pathways in visceral adipose tissue**

### Visceral

**C**

**B**

**D E**

### Visceral

**F G**

**I**

**H**

### Visceral

**J K**

**Online resource supplementary Figure 1:** Kinetic of colonization in metabolic tissue (liver/adipose tissue) and spleen by quantifying by qPCR (V3_V4 16SrRNA primers) in control mice ( normal chow diet) in green or in mice with glycaemic disorders (High Fat Diet) in red

**Online resource supplementary Figure 2:** **A** Stacked Bar Chart representation of the relative abundances of the taxa at the family level for each individual and per tissue.

**Online resource supplementary Figure 3: A-D** Barplot of the relative abundance (%) of the taxa at the family level of the overall individuals and per tissue. Corresponding correlations between subcutaneous and visceral tissues and showed per individuals for Veillonellaceae A, Diplorickettsiaceae B, Campylobacteraceae C, Aeromonadaceae D.

**Online resource supplementary Figure 4: A,** Index of importance of the most discriminant taxa at the family level is shown as calculated by the nonlinear method of Random Forest Analysis

**Online resource supplementary Figure 5: Graphic representation of the inferred metabolic pathway signatures according to tissues and patients.**

**(A) Biplot PCA of pathways** **(B) Principal Coordinate Analysis of inferred metabolic pathway signatures**. Ellipses represent the metabolic pathway signatures corresponding to subcutaneous and visceral fat pads, using PERMANOVA analysis by adonis. **(C)** ANOSIM results and the corresponding significant scores shown as bar plots. **(D)** Clustered Image Maps of the sPLS-DA in component 2 applied to pathway data. **(E)** Sample plots from sPLS-DA performed on the pathways according to tissues, including 95% confidence ellipses. Samples are projected into the space spanned by the first and second components. **(F)** Correlation circle plots from the sPLS-DA applied to pathway data, with a cutoff of 0.6. **(G-H)** Variable Importance in Projection (VIP) coefficients for each predictor pathway and for each sPLS-DA component. **(I)** Pathways selected by VIP coefficients in different adipose tissues.

**Online resource supplementary Figure 6**: **Hierarchal and network representations with clinical parameters of the inferred metabolic pathways signatures.**

**(A)** Clustered Image Maps of Regularized Canonical Correlation Analysis (RCCA) in component 1 between clinical variables and pathways in subcutaneous tissue. **(B)** Clustered Image Maps of Regularized Canonical Correlation Analysis (RCCA) in component 1 between clinical variables and pathways in visceral tissue. **(C)** Regularized Canonical Correlation Analysis (RCCA) networks in component 1 of the top discriminant inferred metabolic pathways with clinical parameters in subcutaneous tissue. **(D)** Regularized Canonical Correlation Analysis (RCCA) networks in component 1 of the top discriminant inferred metabolic pathways with clinical parameters in visceral tissue. The importance of the clinical parameter is shown by the circle size, and the strength of the correlation is shown by the color of the lines between the circles of variables, as indicated on the color scale.

**Online resource supplementary Figure 7:**

**A,C,E,G,I,K,M,O,Q**: Barplots of the relative abundance of the different taxa per individual and per tissue. The most significant Family-level bacteria selected by VIP between the subcutaneous and visceral fat pads of each individual patient. **B,D,F,H,J,L,N,P,R**: graphic representation of the correspondences of the relative abundance of the different Family-level bacteria selected by VIP between the subcutaneous and visceral fat pads of each individual patient.
**S**: Index of importance of the most discriminant pathways is shown as calculated by the nonlinear method of Random Forest Analysis

**Online resource supplementary Figure 8:**

**(A)** Regularized Canonical Correlation Analysis (RCCA) networks in component 2 of the top discriminant inferred metabolic pathways with clinical parameters in subcutaneous tissue. **(B)** Regularized Canonical Correlation Analysis (RCCA) networks in component 2 of the top discriminant inferred metabolic pathways with clinical parameters in visceral tissue. The importance of the clinical parameter is shown by the circle size, and the strength of the correlation is shown by the color of the lines between the circles of variables, as indicated on the color scale.

**Online resource supplementary Figure 9:**

**A,B**: Sparse Partial Least Square Discriminant Analysis of OTUs bacteria in TWL group of both Tissues Adipose. **C,D**: corresponding ROC using the discriminant family-levels bacteria from the sPLS DA. **E,G,I,K,M,O,Q,S,U,W**: Barplots of the relative abundance of the different Family-level bacteria selected by VIP coefficient between TWL group in subcutaneous and visceral fat pads of each individual patient. **F,H,J,L,N,P,R,T,V,X**: graphic representation of the correspondences of the relative abundance of the different Family-level bacteria selected by VIP coefficient between TWL group in subcutaneous and visceral fat pads of each individual patient.

**Online resource supplementary Figure 10**: **Graphic representation of the inferred metabolic pathway signatures in the subcutaneous and visceral adipose tissues predicting the maintenance of body weight loss groups.**

**(A,B)** Venn diagram analyses of the inferred metabolic pathways in subcutaneous common and discriminant between the upper (maintenance of body weight loss) and the lower (no maintenance of body weight loss) groups and Stacked Bar Chart of the most contributing Pathway in the subcutaneous **(A)** and visceral **(B)** adipose tissues. The stacked bar chart contribution in percentage is shown for in the overall cohort studied (Total) and according to the groups of patients who have maintained their body weight loss (low group) or regained weight (upper group) 5-10 years after bariatric surgery. **(C,D)** PCA in subcutaneous **(C)** and visceral **(D)** adipose tissue. **(E)** Clustered Image Maps of the sPLS-DA applied to pathway data in subcutaneous adipose tissue. **(F)** Sample plots from sPLS-DA performed on the pathways according to Lower and Upper groups in subcutaneous tissue, including 95% confidence ellipses. Samples are projected into the space spanned by the first and second components. **(G)** Correlation circle plots from the sPLS-DA applied to pathway data in subcutaneous tissue, with a cutoff of 0.6. **(H,I)** Variable Importance in Projection (VIP) coefficients for each predictor pathway and for each sPLS-DA component 1 **(H)** and component 2 **(I)** in subcutaneous tissue. **(J)** Pathways selected by VIP coefficients in different TWL groups in subcutaneous tissue.

**Online resource supplementary Figure 11**: **Graphic representation of the inferred metabolic pathway signatures in visceral adipose tissues predicting the maintenance of body weight loss groups.**

**(A)** Clustered Image Maps of the sPLS-DA applied to pathway data in visceral adipose tissue. **(B)** Sample plots from sPLS-DA performed on the pathways according to Lower and Upper groups in visceral adipose tissue, including 95% confidence ellipses. Samples are projected into the space spanned by the first and second components. **(C)** Correlation circle plots from the sPLS-DA applied to pathway data in visceral tissue, with a cutoff of 0.6. **(D,E)** Variable Importance in Projection (VIP) coefficients for each predictor pathway and for each sPLS-DA component 1 **(D)** and component 2 **(E)** in visceral tissue. **(F)** Pathways selected by VIP coefficients in different Upper and Lower groups in visceral tissue.

**Online resource supplementary Figure 12:**

**A,C,E,G,I,K,M,O,Q**: Barplots of the relative abundance of the different pathways selected by VIP coefficient between TWL group in subcutaneous fat pads of each individual patient.

**B,D,F,H,J,L,N,P,R**: graphic representation of the correspondences of the relative abundance of the different pathways selected by VIP coefficient between TWL group in subcutaneous fat pads of each individual patient.

**S:** Discriminant analysis ROC sPLS-DA of microbial metabolic pathways in subcutaneous adipose tissue.

**Online resource supplementary Figure 13:**

**A**: Discriminant analysis ROC sPLS-DA of microbial metabolic pathways in visceral adipose tissue.

**B,D,F,H,J**: Barplots of the relative abundance of the different pathways selected by VIP coefficient between TWL group in visceral fat pads of each individual patient.

**C,E,G,I,K**: graphic representation of the correspondences of the relative abundance of the different pathways selected by VIP coefficient between TWL group in Visceral fat pads of each individual patient.

| **Clinical features** | **All patients (n=63)** |
| --- | --- |
| AUC Ins mmol/L/120min | 13529.4 ± 8776.7 |
| 16SrRNA | 0.42 ± 0.19 |
| Cortisol µg/dL | 13.24 ± 5.15 |
| Creatinine mg/dL | 0.75 ± 0.15 |
| Ferritin ng/mL | 85.87 ± 113.60 |
| Free T4 ng/dL | 1.18 ± 0.19 |
| GOT U/L | 23.53 ± 17.29 |
| GPT U/L | 34.89 ± 22.03 |
| GGT U/L | 30.85 ± 18.93 |
| IVGTT Insulin mmol/L/60min | 5631.99 ± 3125.29 |
| IVGTT Glycemia mmol/L/60min | 1111.50 ± 1338.17 |
| mcrclamp % | 3.59 ± 2.18 |
| mscclamp mg/kg.min | 3.38 ± 2.24 |
| ogtt120 mg/dL | 133.94 ± 37.25 |
| ogtt30 mg/dL | 142.8077 ± 30.11 |
| ogtt60 mg/dL | 154.82 ± 32.42 |
| ogtt90 mg/dL | 149.83 ± 34.77 |
| ogtti120 mcUI/mL | 119.89 ± 82.84 |
| ogttinsb mcUI/mL | 30.29 ± 39.38 |
| sbp mmHg | 137.79 ± 18.90 |

**Online resource, Table 1: Clinical characteristics of the patients as means +/-standard error**
